# Supplementary material for: Aurora kinase A inhibition reverses the Warburg effect and elicits unique metabolic vulnerabilities in glioblastoma
Source: Nat Commun. 2021 Sep 1;12:5203. doi: 10.1038/s41467-021-25501-x (PMC8410792; doi:10.1038/s41467-021-25501-x)
Supplement: Supplementary file 1 — Supplementary Information [file 41467_2021_25501_MOESM1_ESM.pdf]

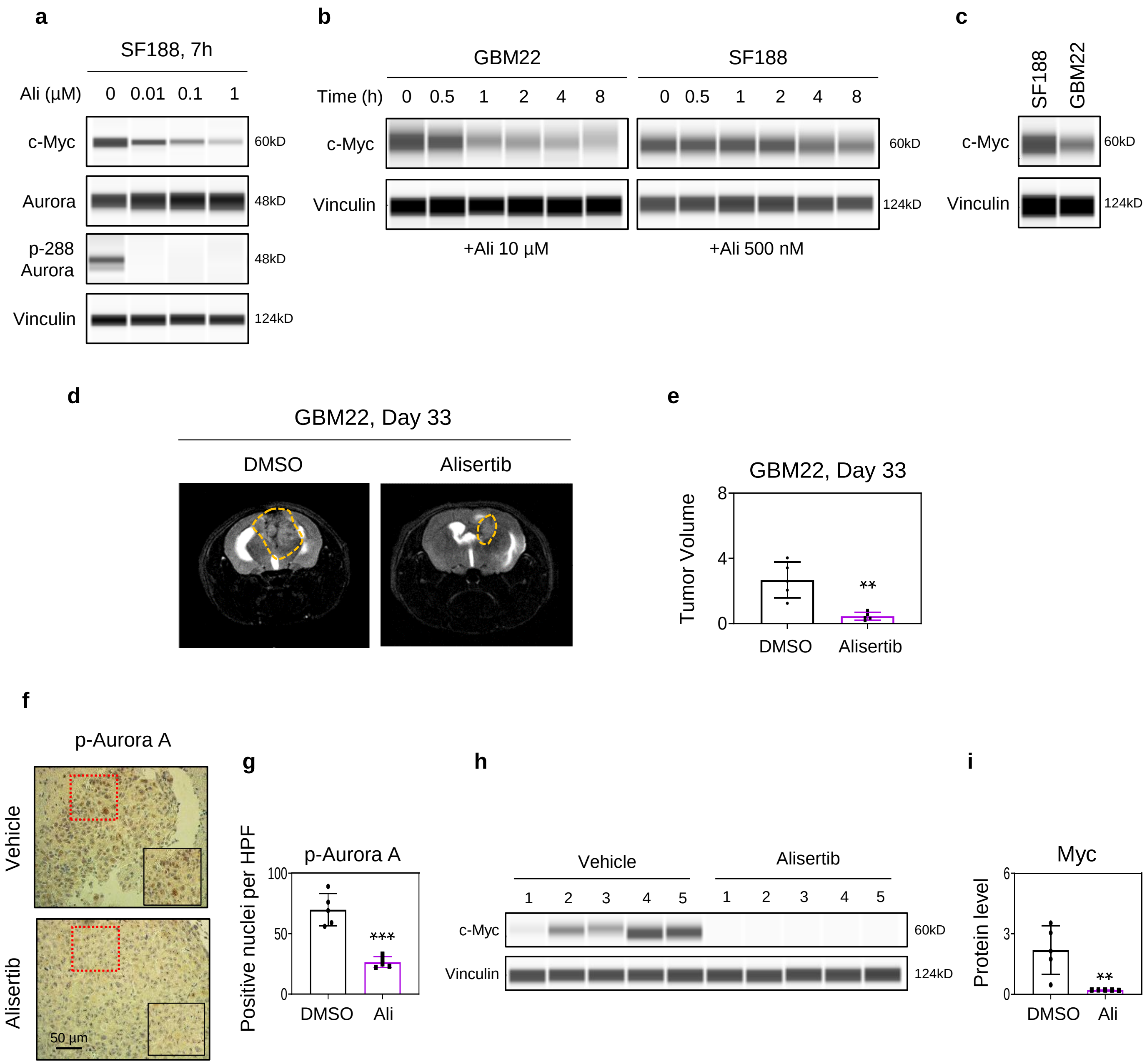
a
SF188, 7h
Ali (μM) 0 0.01 0.1 1
c-Myc
Aurora
p-288 Aurora
Vinculin
60kD
48kD
48kD
124kD
b
GBM22
SF188
Time (h) 0 0.5 1 2 4 8
c-Myc
Vinculin
60kD
124kD
+Ali 10 μM
+Ali 500 nM
c
SF188
GBM22
c-Myc
Vinculin
60kD
124kD
d
GBM22, Day 33
DMSO
Alisertib
e
GBM22, Day 33
Tumor Volume
DMSO
Alisertib
**
f
p-Aurora A
Vehicle
Alisertib
50 μm
g
p-Aurora A
Positive nuclei per HPF
DMSO
Ali
***
h
Vehicle
Alisertib
1 2 3 4 5
c-Myc
Vinculin
60kD
124kD
i
Myc
Protein level
DMSO
Ali
**

Supplementary Figure 1: Aurora kinase A regulates c-Myc protein levels by modulation of its stability. a Shown is a protein capillary electrophoresis of SF188 cells treated with DMSO or alisertib for 7h with indicated protein. Vinculin is used as a loading control. **b** Shown is the c-Myc protein level of SF188 and GBM22 cells treated with alisertib for 30 min, 1h, 2h, 4h, and 5h. Vinculin is used as a loading control. **c** Shown is the c-Myc protein level in SF188 and GBM22 cells. For **d, e** GBM22 cells were implanted in the right striatum of nude mice. Two groups were randomly assigned: vehicle and alisertib after seven days of the implantation. Mice were treated three times per week. Shown is the tumor size of representative MRI images using a Bruker BioSpecTM, 9.4 Tesla MR imager of vehicle and alisertib treated GBM22 tumors. The quantification is shown in **e** (n=5 independent tumors) (**p=0.0023). For **f, g** Brain tumors from the experiment in **d** were fixed and stained with p-Aurora A antibody. The quantification is shown in **g** (n=5 independent high-power field microscopy) (***p=0.0001). For **h, i** Shown is a protein capillary electrophoresis of brain tumors from the experiment in **d** with c-Myc protein level. The quantification is shown in **i** (n=5 independent tumors) (**p=0.0061). Scale bar: 50 µm. Statistical significance was assessed by two-tailed student's t-test. Data are shown as mean ± SD in **e, g, i**. Source data are provided as a Source Data file.

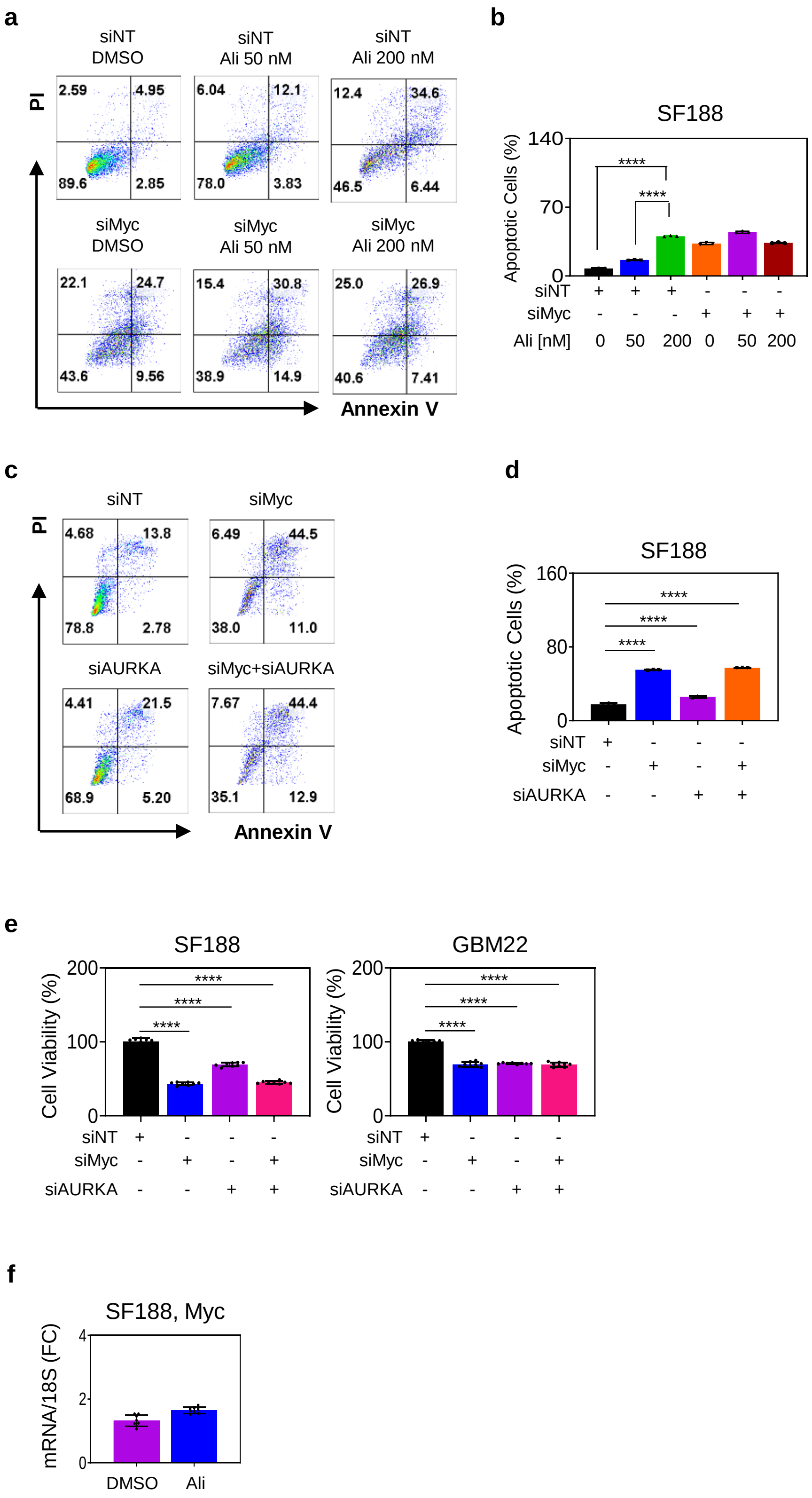
a
siNT DMSO
siNT Ali 50 nM
siNT Ali 200 nM
PI
2.59 4.95 89.6 2.85
6.04 12.1 78.0 3.83
12.4 34.6 46.5 6.44
siMyc DMSO
siMyc Ali 50 nM
siMyc Ali 200 nM
22.1 24.7 43.6 9.56
15.4 30.8 38.9 14.9
25.0 26.9 40.6 7.41
Annexin V
b
SF188
Apoptotic Cells (%)
140
70
0
****
****
siNT + + + - - -
siMyc - - - + + +
Ali [nM] 0 50 200 0 50 200
c
siNT
siMyc
PI
4.68 13.8 78.8 2.78
6.49 44.5 38.0 11.0
siAURKA
siMyc+siAURKA
4.41 21.5 68.9 5.20
7.67 44.4 35.1 12.9
Annexin V
d
SF188
Apoptotic Cells (%)
160
80
0
****
****
****
siNT + - - -
siMyc - + - +
siAURKA - - + +
e
SF188
GBM22
Cell Viability (%)
200
100
0
****
****
****
siNT + - - -
siMyc - + - +
siAURKA - - + +
f
SF188, Myc
mRNA/18S (FC)
4
2
0
DMSO
Ali

Supplementary Figure 2: Aurora kinase A regulates cell viability through c-Myc. For **a, b** SF188 cells were transfected with non-targeting or c-Myc specific siRNA and treated with increasing concentrations of alisertib for 48h, labeled with Annexin/PI dye and analyzed by flow cytometry. The quantification is shown in **b** (n=3 independent samples) (****p<0.0001). For **c, d** SF188 cells were transfected with non-targeting siRNA or specific siRNA targeting Myc, AURKA, or the combination of both, labeled with Annexin/PI dye and analyzed by flow cytometry. The quantification is shown in **d** (n=3 independent samples) (****p<0.0001). **e** GBM22 and SF188 cells were transfected with non-targeting siRNA or specific siRNA targeting c-Myc, AURKA, or combination of both and cellular viability was analyzed (n=8 independent samples) (****p<0.0001). **f** Real time PCR analysis of c-Myc mRNA levels of SF188 cells treated with 100 nM alisertib for 24h (n=8 independent samples). Statistical significance was determined by one-way ANOVA with Dunnett's multiple comparison test in **b, d, e**. Data are shown as mean ± SD in **b, d-f**. Source data are provided as a Source Data file.

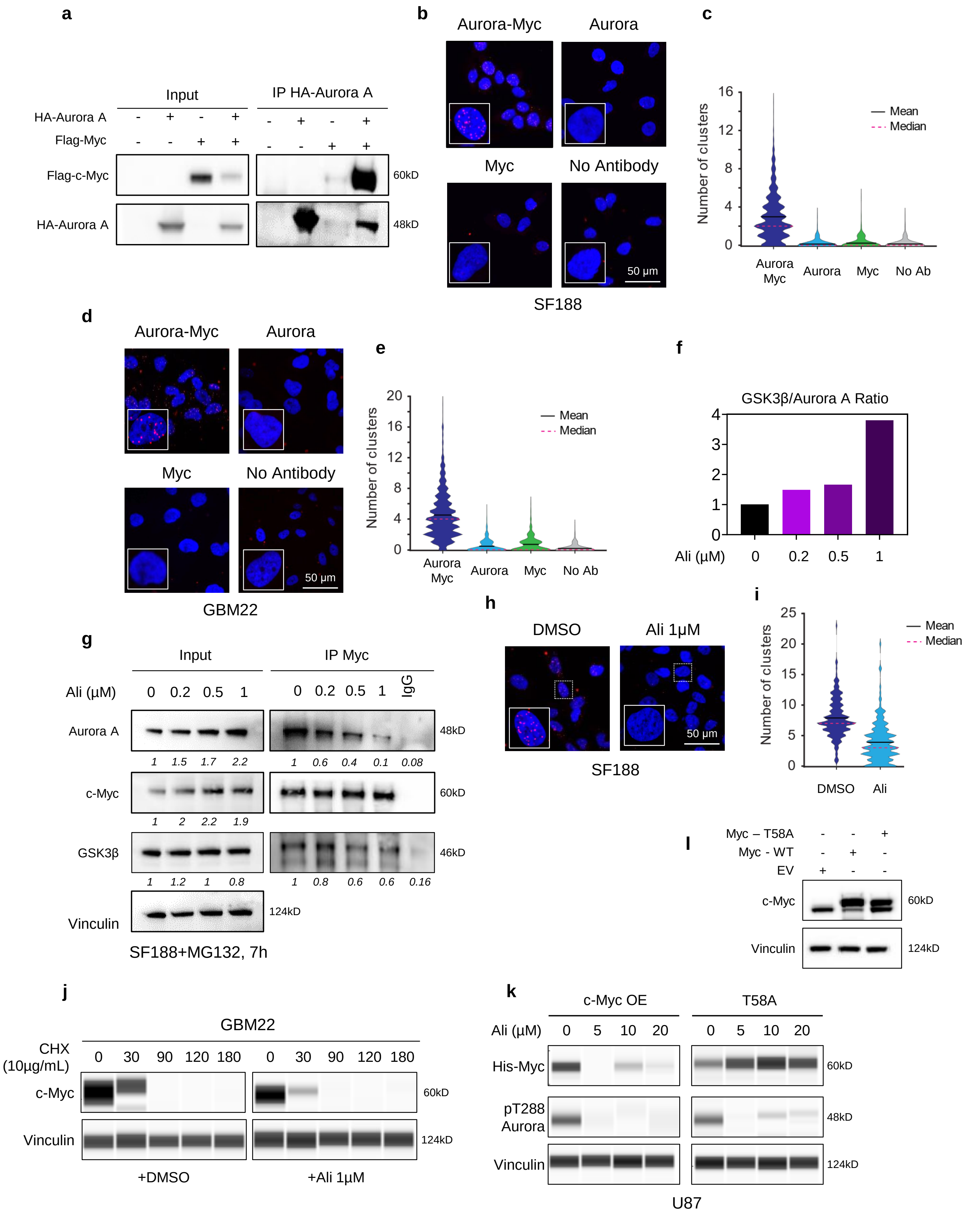
a
Input
IP HA-Aurora A
HA-Aurora A - + - + - + - +
Flag-Myc - - + + - - + +
Flag-c-Myc
HA-Aurora A
60kD
48kD
b
Aurora-Myc
Aurora
Myc
No Antibody
50 μm
SF188
c
Number of clusters
Mean
Median
Aurora Myc
Aurora
Myc
No Ab
d
Aurora-Myc
Aurora
Myc
No Antibody
50 μm
GBM22
e
Number of clusters
Mean
Median
Aurora Myc
Aurora
Myc
No Ab
f
GSK3β/Aurora A Ratio
Ali (μM) 0 0.2 0.5 1
g
Input
IP Myc
Ali (μM) 0 0.2 0.5 1 0 0.2 0.5 1 IgG
Aurora A
1 1.5 1.7 2.2 1 0.6 0.4 0.1 0.08
c-Myc
1 2 2.2 1.9
GSK3β
1 1.2 1 0.8 1 0.8 0.6 0.6 0.16
Vinculin
48kD
60kD
46kD
124kD
SF188+MG132, 7h
h
DMSO
Ali 1μM
50 μm
SF188
i
Number of clusters
Mean
Median
DMSO
Ali
l
Myc – T58A - - +
Myc - WT - + -
EV + - -
c-Myc
Vinculin
60kD
124kD
j
GBM22
CHX (10μg/mL) 0 30 90 120 180 0 30 90 120 180
c-Myc
Vinculin
60kD
124kD
+DMSO
+Ali 1μM
k
c-Myc OE
T58A
Ali (μM) 0 5 10 20 0 5 10 20
His-Myc
pT288 Aurora
Vinculin
60kD
48kD
124kD
U87

Supplementary Figure 3: Aurora kinase A binds to c-Myc protein. a Standard western blots of 293T cells transfected with HA-Aurora kinase A and Flag-c-Myc for 24h. Cells were lysed and lysates were immunoprecipitated with an anti-HA antibody immobilized on beads and probed with the indicated antibodies. For **b-e** Proximity ligation assays analyzing interaction between c-Myc and Aurora kinase A in SF188 and GBM22. Blue is DAPI staining and red is PCR amplification products indicating complex formation of c-Myc and Aurora kinase A. Quantification of c-Myc and Aurora kinase A interaction was shown in **c** and **e,** respectively. Scale bar: 50 µm. For **f, g** SF188 cells were treated with DMSO or alisertib in the presence or absence of 5 µM of MG132 for 7h. Subsequently, cells lysates were immunoprecipitated with IgG or c-Myc antibody and probed with indicated antibodies. The ratio between GSK3β and Aurora is shown in **f**. For **h, i** Proximity ligation assays analyzing interaction between c-Myc and Aurora kinase A in SF188 treated with DMSO or 1 µM alisertib. Quantification of c-Myc and Aurora kinase A interaction was shown in **i**. **j** GBM22 cells were treated with DMSO or alisertib in the presence or absence of 10 µg/mL cycloheximide (CHX) and the whole cell lysates were subjected to protein capillary electrophoresis and analyzed for the indicated proteins. **k** U87 cells were transfected with c-Myc-WT or c-Myc mutant (T58A), treated with the indicated concentrations of alisertib for 24h, and the whole cell lysates were subjected to protein capillary electrophoresis. **l** Standard western blot of c-Myc protein expression in GBM22 cells infected with EV, c-Myc-T58A, and c-Myc-WT. Source data are provided as a Source Data file.

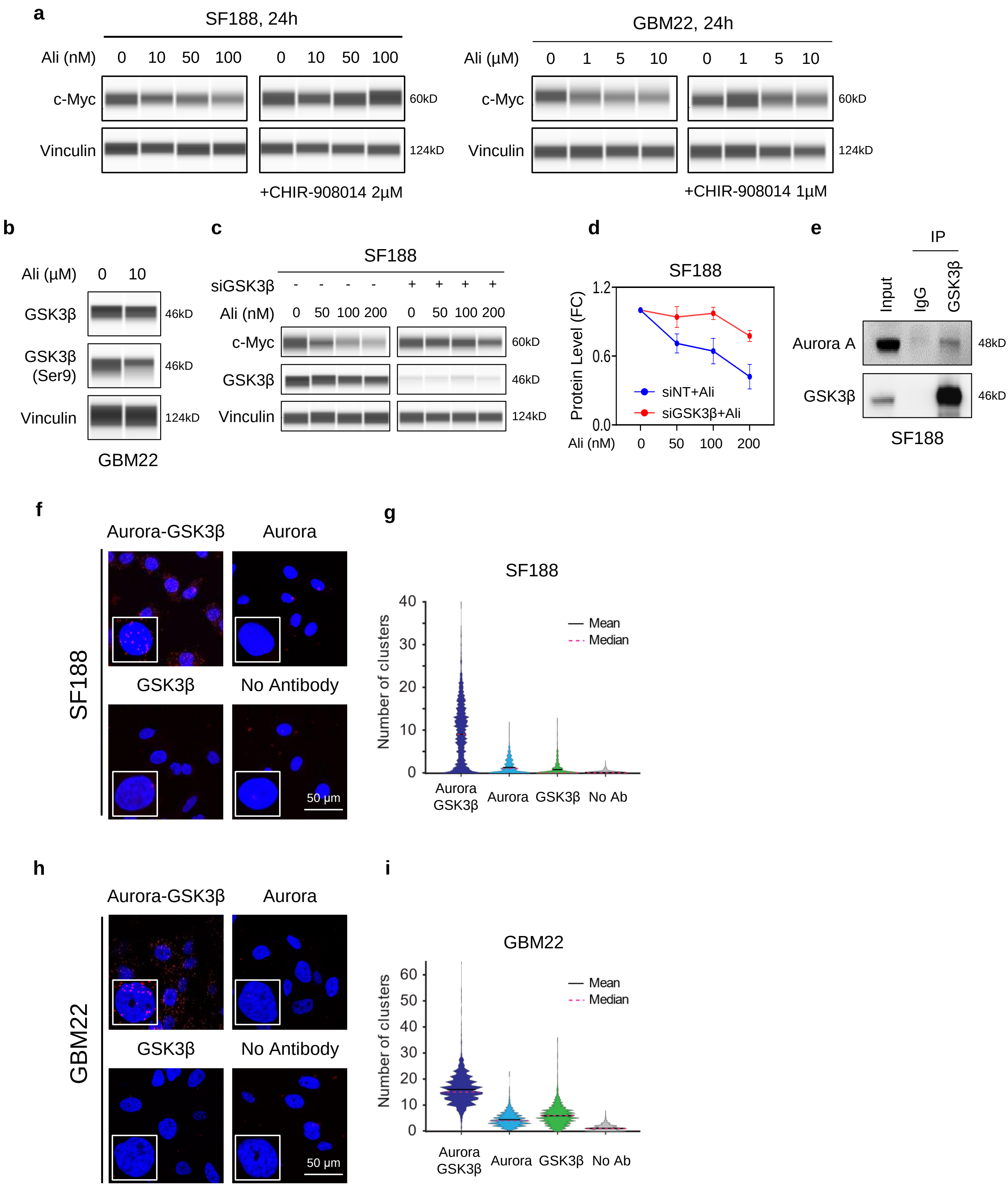
a
SF188, 24h
Ali (nM) 0 10 50 100 0 10 50 100
c-Myc
Vinculin
60kD
124kD
+CHIR-908014 2μM
GBM22, 24h
Ali (μM) 0 1 5 10 0 1 5 10
c-Myc
Vinculin
60kD
124kD
+CHIR-908014 1μM
b
Ali (μM) 0 10
GSK3β
GSK3β (Ser9)
Vinculin
46kD
46kD
124kD
GBM22
c
SF188
siGSK3β - - - - + + + +
Ali (nM) 0 50 100 200 0 50 100 200
c-Myc
GSK3β
Vinculin
60kD
46kD
124kD
d
SF188
Protein Level (FC)
1.2
0.6
0.0
siNT+Ali
siGSK3β+Ali
Ali (nM) 0 50 100 200
e
IP
Input
IgG
GSK3β
Aurora A
GSK3β
48kD
46kD
SF188
f
Aurora-GSK3β
Aurora
GSK3β
No Antibody
SF188
50 μm
g
SF188
Number of clusters
40
30
20
10
0
Mean
Median
Aurora GSK3β
Aurora
GSK3β
No Ab
h
Aurora-GSK3β
Aurora
GSK3β
No Antibody
GBM22
50 μm
i
GBM22
Number of clusters
60
50
40
30
20
10
0
Mean
Median
Aurora GSK3β
Aurora
GSK3β
No Ab

Supplementary Figure 4: Aurora kinase A binds to GSK3β protein. a SF188 and GBM22 cells were treated with increasing concentration of alisertib in the presence or absence of CHIR-908014 (GSK3β inhibitor) for 24h and were subjected to protein capillary electrophoresis. **b** GBM22 cells were treated with 10 µM alisertib for 24h and were subjected to protein capillary electrophoresis. For **c, d** SF188 cells were transfected with non-targeting siNT or siGSK3β, treated with increasing concentrations of alisertib for 24h, and were subjected to protein capillary electrophoresis for the indicated proteins. Quantification of c-Myc protein level was shown in **d** (n=3 independent samples). **e** SF188 cell lysates were immunoprecipitated with IgG or GSK3β antibody and analyzed by standard western blotting. Input: cell lysate loading control. IgG: negative control. For **f-i** Proximity ligation assays analyzing interaction between GSK3β and Aurora kinase A in SF188 and GBM22. Blue is DAPI staining and red is PCR amplification products indicating complex formation of GSK3β and Aurora kinase A. Quantification of GSK3β and Aurora interaction was shown in **g** and **i**, respectively. Scale bar: 50 µm. Data are shown as mean ± SD in **d**. Source data are provided as a Source Data file.

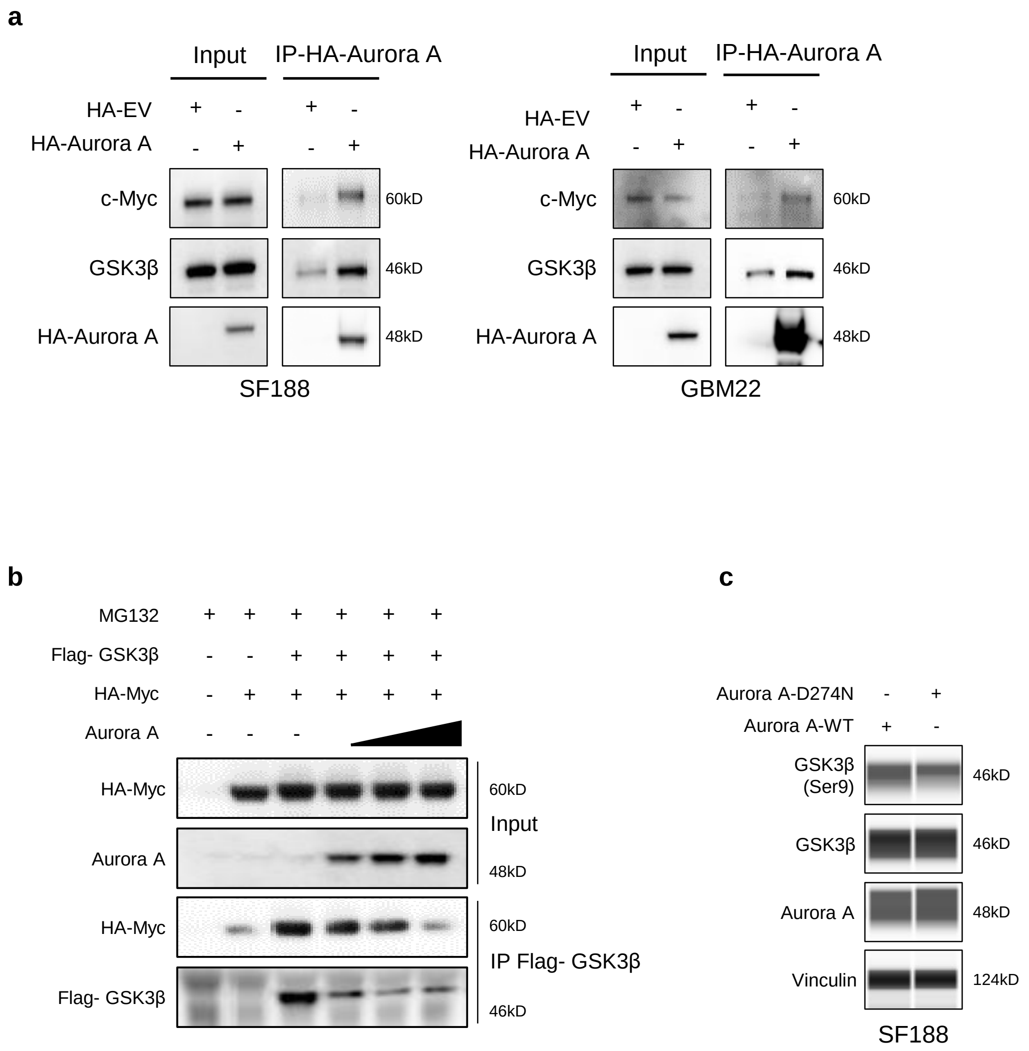
a
Input
IP-HA-Aurora A
HA-EV
HA-Aurora A
c-Myc
GSK3β
HA-Aurora A
60kD
46kD
48kD
SF188
GBM22
b
MG132
Flag- GSK3β
HA-Myc
Aurora A
HA-Myc
Aurora A
Flag- GSK3β
60kD
48kD
46kD
Input
IP Flag- GSK3β
c
Aurora A-D274N
Aurora A-WT
GSK3β (Ser9)
GSK3β
Aurora A
Vinculin
46kD
48kD
124kD
SF188

Supplementary Figure 5: Protein interaction between c-Myc and GSK3β in the presence of Aurora kinase A. a Standard western blot of SF188 and GBM22 cells transfected with HA-EV and HA-Aurora kinase A for 24h. Cells were lysed and cell lysates were immunoprecipitated with an anti-HA antibody immobilized on beads and probed with the indicated antibodies. **b** Standard western blot of 293T cells transfected with Flag-GSK3β, HA-c-Myc, and/ or increasing concentration Aurora kinase A (0.25, 0.5, and 1 μg) in the presence of 5 μM MG132 for 48h. Cells were lysed and cell lysates were immunoprecipitated with an anti-FLAG antibody immobilized on beads and probed with the indicated antibodies. **c** SF188 cells transfected with HA-Aurora kinase A-WT and HA-Aurora kinase A-D274N for 24h and were subjected to protein capillary electrophoresis for the indicated proteins.

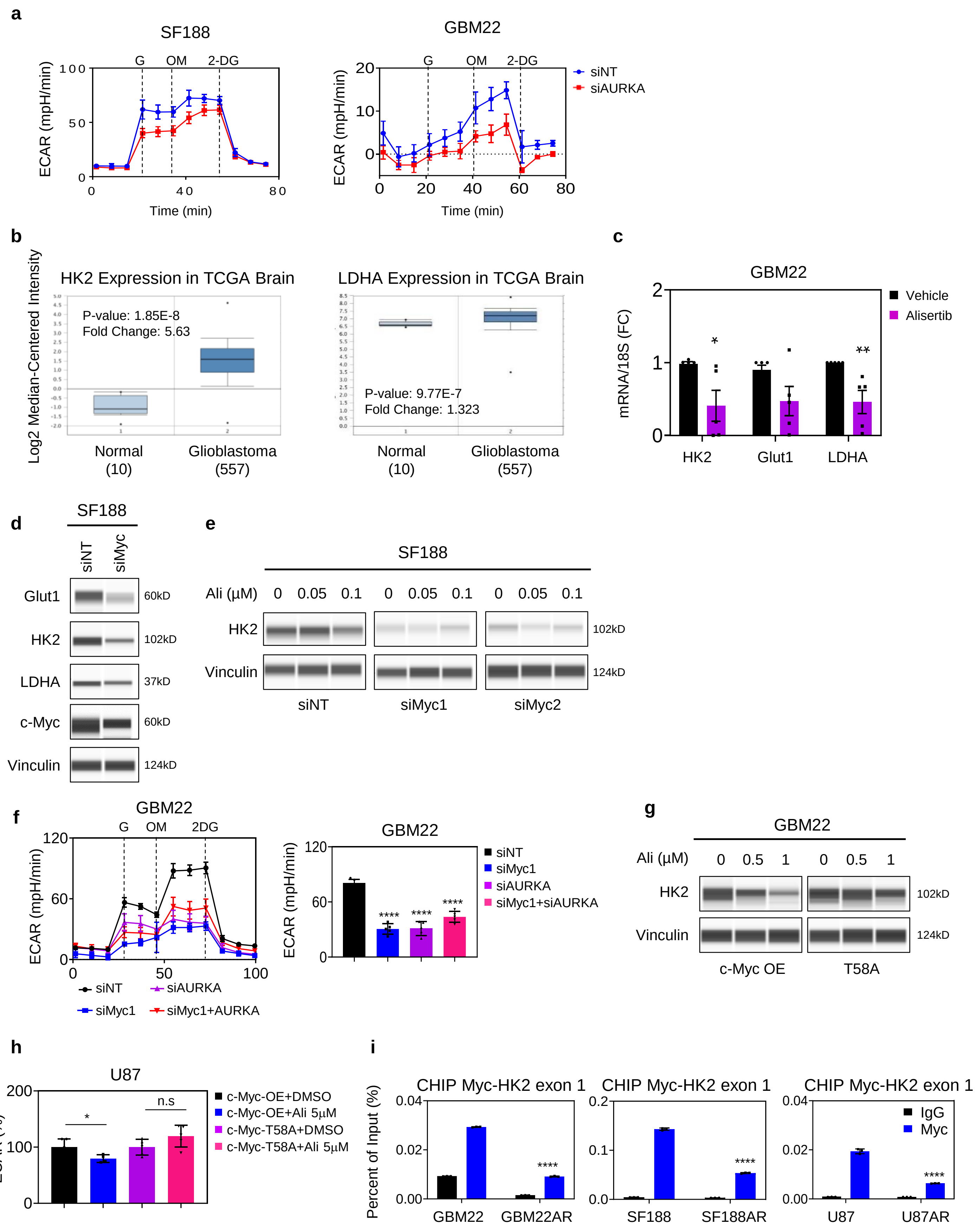

a
SF188
GBM22
G OM 2-DG
ECAR (mpH/min)
Time (min)
siNT
siAURKA
b
HK2 Expression in TCGA Brain
LDHA Expression in TCGA Brain
Log2 Median-Centered Intensity
P-value: 1.85E-8
Fold Change: 5.63
P-value: 9.77E-7
Fold Change: 1.323
Normal (10)
Glioblastoma (557)
c
GBM22
mRNA/18S (FC)
Vehicle
Alisertib
HK2 Glut1 LDHA
d
SF188
siNT siMyc
Glut1 60kD
HK2 102kD
LDHA 37kD
c-Myc 60kD
Vinculin 124kD
e
SF188
Ali (μM) 0 0.05 0.1 0 0.05 0.1 0 0.05 0.1
HK2 102kD
Vinculin 124kD
siNT siMyc1 siMyc2
f
GBM22
G OM 2DG
ECAR (mpH/min)
siNT
siAURKA
siMyc1
siMyc1+AURKA
siMyc1+siAURKA
g
GBM22
Ali (μM) 0 0.5 1 0 0.5 1
HK2 102kD
Vinculin 124kD
c-Myc OE T58A
h
U87
ECAR (%)
n.s
c-Myc-OE+DMSO
c-Myc-OE+Ali 5μM
c-Myc-T58A+DMSO
c-Myc-T58A+Ali 5μM
i
CHIP Myc-HK2 exon 1
Percent of Input (%)
IgG
Myc
GBM22 GBM22AR
SF188 SF188AR
U87 U87AR

Supplementary Figure 6: Aurora kinase A regulates glycolysis at least in part through c-Myc. a SF188 and GBM22 cells were transfected with specific siRNA targeting AURKA and analyzed in the context of a glycolysis stress assay. Extracellular acidification rate (ECAR) is recorded at baseline and following injection of Glucose (G), Oligomycin (OM), and 2-DG. **b** Shown are the mRNA levels of HK2 and LDHA in the TCGA glioblastoma database. Data was extracted from oncomine.org. Middle lines in boxplot: median; box ranges: upper: 75th percentile; lower: 25th percentile; error bars: 10th and 90th percentile. **c** Real time PCR analysis of glycolysis mRNA levels in GBM22 brain tumor treated with vehicle or alisertib (n=5 independent samples) (*p=0.0288, **p=0.0093). **d** SF188 cells were transfected with a specific siMyc and the whole cell lysates were subjected to protein capillary electrophoresis. **e** SF188 cells were transfected with two specific siRNA targeting Myc, treated with increasing concentration of alisertib for 24h, and the whole cell lysates were subjected to protein capillary electrophoresis with indicated antibodies. **f** GBM22 cells were transfected with specific siRNA targeting Myc, AURKA, or the combination of both, and were analyzed in the context of a glycolysis stress assay on a Seahorse XFe24 extracellular flux analyzer. The graph on the right shows the quantification of glycolytic activity (n=5 independent samples) (****p<0.0001). **g** GBM22 cells were transfected with c-Myc-WT and c-Myc-T58A, treated with increasing concentration of alisertib, and the whole cell lysates were subjected to protein capillary electrophoresis. **h** U87 cells were transfected with cMyc-WT and c-Myc-T58A, treated with 5 µM alisertib for 24h, and analyzed in the context of a glycolysis stress assay on a Seahorse XFe24 extracellular flux analyzer. Shown is the glycolytic level (n=5 independent samples) (*p=0.0369, n.s: not significant). **i** Parental or chronically alisertib treated GBM22, SF188, and U87 cells were subjected to CHIP with an IgG as a negative control or a Myc specific antibody. The HK2 region was amplified by PCR (n=3 independent samples) (****p<0.0001). Statistical significance was assessed by two-tailed student's t-test. Data are shown as mean ± SD in **a, c, f, h-i**. Source data are provided as a Source Data file.

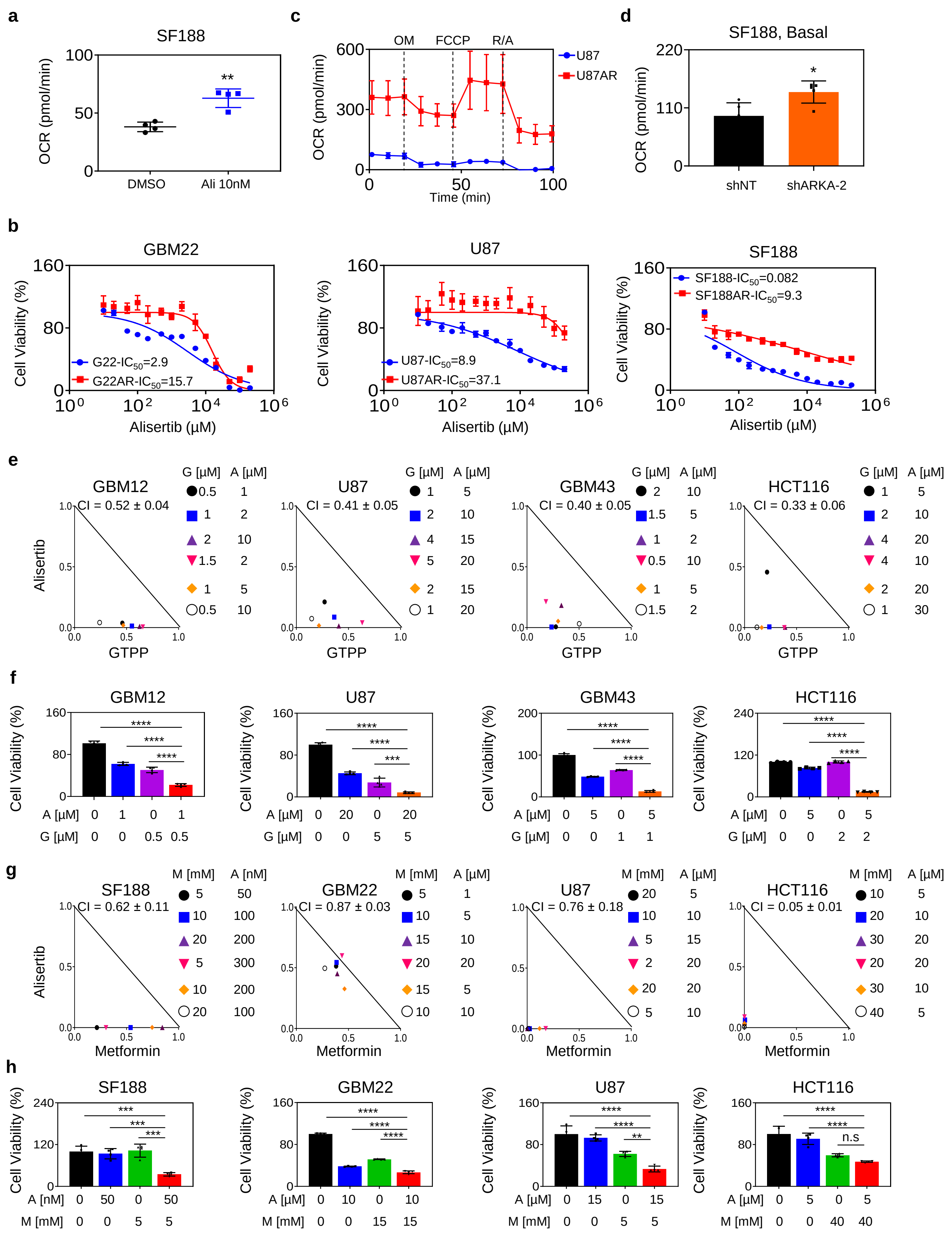
a
SF188
OCR (pmol/min)
100
50
0
**
DMSO
Ali 10nM
c
OM
FCCP
R/A
600
300
0
OCR (pmol/min)
0
50
100
Time (min)
U87
U87AR
d
SF188, Basal
220
110
0
OCR (pmol/min)
*
shNT
shARKA-2
b
GBM22
160
80
0
Cell Viability (%)
G22-IC50=2.9
G22AR-IC50=15.7
Alisertib (μM)
U87
U87-IC50=8.9
U87AR-IC50=37.1
Alisertib (μM)
SF188
SF188-IC50=0.082
SF188AR-IC50=9.3
Alisertib (μM)
e
GBM12
CI = 0.52 ± 0.04
G [μM] A [μM]
0.5 1
1 2
2 10
1.5 2
1 5
0.5 10
Alisertib
GTPP
U87
CI = 0.41 ± 0.05
G [μM] A [μM]
1 5
2 10
4 15
5 20
2 15
1 20
GTPP
GBM43
CI = 0.40 ± 0.05
G [μM] A [μM]
2 10
1.5 5
1 2
0.5 10
1 5
1.5 2
GTPP
HCT116
CI = 0.33 ± 0.06
G [μM] A [μM]
1 5
2 10
4 20
4 10
2 20
1 30
GTPP
f
GBM12
Cell Viability (%)
****
****
****
A [μM] 0 1 0 1
G [μM] 0 0 0.5 0.5
U87
****
****
***
A [μM] 0 20 0 20
G [μM] 0 0 5 5
GBM43
****
****
****
A [μM] 0 5 0 5
G [μM] 0 0 1 1
HCT116
****
****
****
A [μM] 0 5 0 5
G [μM] 0 0 2 2
g
SF188
CI = 0.62 ± 0.11
M [mM] A [nM]
5 50
10 100
20 200
5 300
10 200
20 100
Alisertib
Metformin
GBM22
CI = 0.87 ± 0.03
M [mM] A [μM]
5 1
10 5
15 10
20 20
15 5
10 10
Metformin
U87
CI = 0.76 ± 0.18
M [mM] A [μM]
20 5
10 10
5 15
2 20
20 20
5 10
Metformin
HCT116
CI = 0.05 ± 0.01
M [mM] A [μM]
10 5
20 10
30 20
20 20
30 10
40 5
Metformin
h
SF188
Cell Viability (%)
***
***
***
A [nM] 0 50 0 50
M [mM] 0 0 5 5
GBM22
****
****
****
A [μM] 0 10 0 10
M [mM] 0 0 15 15
U87
****
****
**
A [μM] 0 15 0 15
M [mM] 0 0 5 5
HCT116
****
****
n.s
A [μM] 0 5 0 5
M [mM] 0 0 40 40

Supplementary Figure 7: Aurora kinase A inhibition elicits the evolvement of a hyper-oxidative phenotype that can be targeted through inhibition of mitochondrial metabolism. **a** SF188 cells were treated with 10 nM alisertib for 24h and analyzed for oxygen consumption rate (OCR) on a Seahorse XFe24 device. Shown is the OCR level (n=4 independent samples) (**p=0.0016). **b** Parental or chronically alisertib exposed GBM22, U87, and SF188 cells were treated with increasing concentration of alisertib for 72h, and cellular viability was analyzed (n=4 independent samples). IC_{50} in µM range. **c** Parental or chronically alisertib treated U87 cells were analyzed for the oxygen consumption rate (OCR) on a Seahorse XFe24 device. **d** SF188 cells were transduced with a scrambled or AURKA shRNA and analyzed for the oxygen consumption rate (OCR) on a Seahorse XFe24 device. Shown is the OCR level (n=5 independent samples) (*p=0.0144). For **e, f** GBM12, U87, GBM43, and HCT116 cells were treated with the indicated concentrations of alisertib and GTPP for 72h and cellular viability was analyzed. Shown are the isobolograms. A quantification is shown in **f** (n=4 independent samples) (***p=0.0005, ****p<0.0001). For **g, h** SF188, GBM22, U87, and HCT116 cells were treated with the indicated concentrations of alisertib and metformin for 72h and cellular viability was performed. Shown are the isobolograms. Quantification is shown in **h** (n=4 independent samples) (SF188: DMSO vs Combination: ***p=0.0002, Alisertib vs Combination: ***p=0.0004, Metformin vs Combination: ***p=0.0001, **p=0.0038, n.s: not significant). Statistical significance was determined by two-tailed student's t-test in **a, d** or by one-way ANOVA with Dunnett's multiple comparison test in **f, h**. Data are shown as mean ± SD in **a-d, f, h**. Source data are provided as a Source Data file.

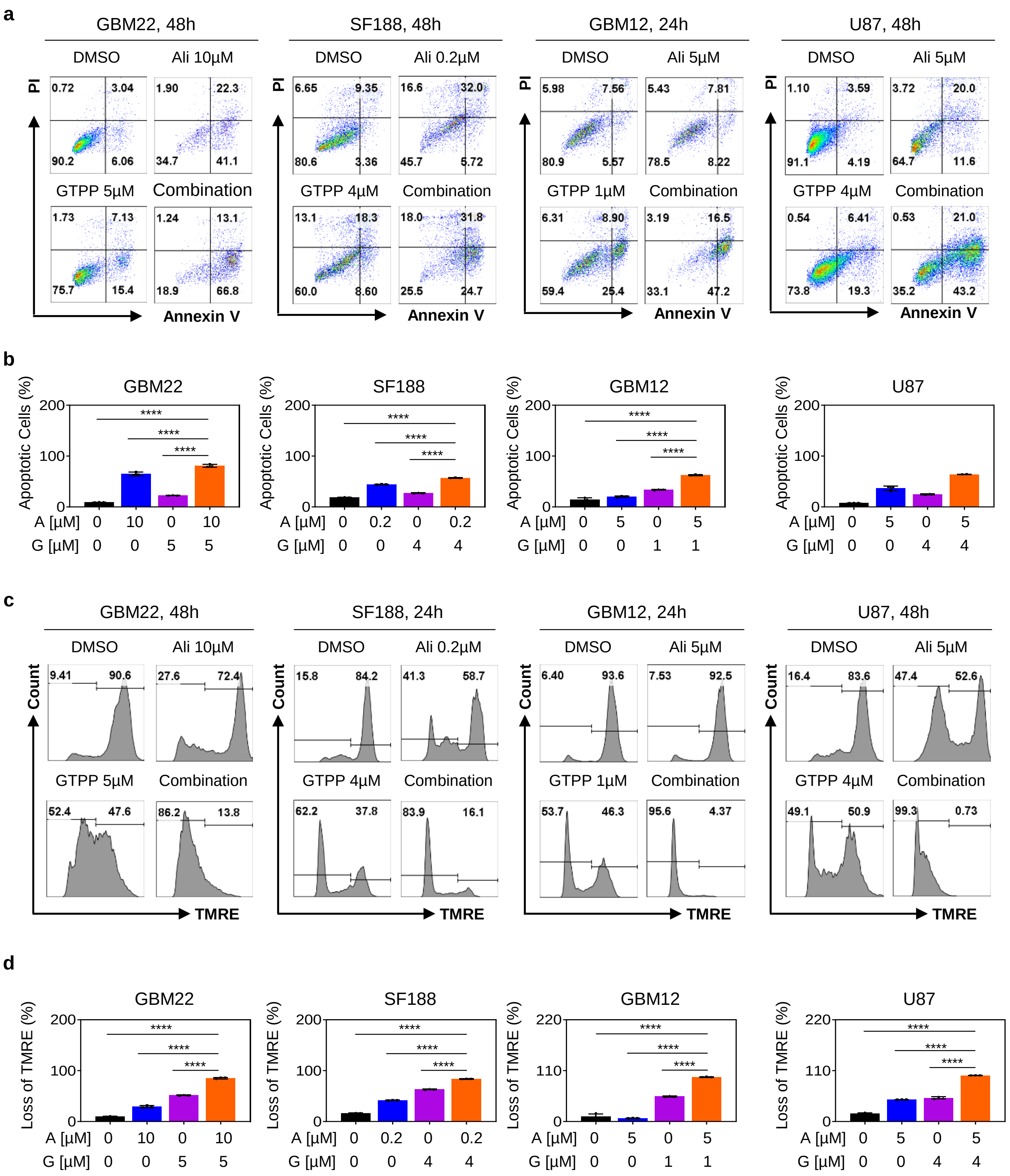
a
GBM22, 48h
DMSO
Ali 10μM
GTPP 5μM
Combination
PI
Annexin V
0.72 3.04 90.2 6.06
1.90 22.3 34.7 41.1
1.73 7.13 75.7 15.4
1.24 13.1 18.9 66.8
SF188, 48h
DMSO
Ali 0.2μM
GTPP 4μM
Combination
6.65 9.35 80.6 3.36
16.6 32.0 45.7 5.72
13.1 18.3 60.0 8.60
18.0 31.8 25.5 24.7
GBM12, 24h
DMSO
Ali 5μM
GTPP 1μM
Combination
5.98 7.56 80.9 5.57
5.43 7.81 78.5 8.22
6.31 8.90 59.4 25.4
3.19 16.5 33.1 47.2
U87, 48h
DMSO
Ali 5μM
GTPP 4μM
Combination
1.10 3.59 91.1 4.19
3.72 20.0 64.7 11.6
0.54 6.41 73.8 19.3
0.53 21.0 35.2 43.2
b
GBM22
SF188
GBM12
U87
Apoptotic Cells (%)
200
100
0
****
A [μM] 0 10 0 10
G [μM] 0 0 5 5
A [μM] 0 0.2 0 0.2
G [μM] 0 0 4 4
A [μM] 0 5 0 5
G [μM] 0 0 1 1
A [μM] 0 5 0 5
G [μM] 0 0 4 4
c
GBM22, 48h
SF188, 24h
GBM12, 24h
U87, 48h
DMSO
Ali 10μM
Ali 0.2μM
Ali 5μM
GTPP 5μM
GTPP 4μM
GTPP 1μM
Combination
Count
TMRE
9.41 90.6
27.6 72.4
52.4 47.6
86.2 13.8
15.8 84.2
41.3 58.7
62.2 37.8
83.9 16.1
6.40 93.6
7.53 92.5
53.7 46.3
95.6 4.37
16.4 83.6
47.4 52.6
49.1 50.9
99.3 0.73
d
GBM22
SF188
GBM12
U87
Loss of TMRE (%)
200
100
0
220
110
****
A [μM] 0 10 0 10
G [μM] 0 0 5 5
A [μM] 0 0.2 0 0.2
G [μM] 0 0 4 4
A [μM] 0 5 0 5
G [μM] 0 0 1 1
A [μM] 0 5 0 5
G [μM] 0 0 4 4

Supplementary Figure 8: The combination treatment of gamitrinib and alisertib enhances induction of a cell death with apoptotic features. For **a, b** GBM22, SF188, GBM12, and U87 cells were treated with alisertib, GTPP or combination of both, labeled with Annexin/PI dye and analyzed by flow cytometry. A quantification is shown in **b** (n=3 in GBM22, SF188, and GBM12; U87: n=2 in GTPP and combination, n=3 in DMSO and alisertib independent samples) (****p<0.0001). For **c, d** GBM22, SF188, GBM12, and U87 cells were treated with alisertib, GTPP or combination of both, labeled with TMRE dye and analyzed by flow cytometry. A quantification is shown in **d** (n=3 independent samples) (****p<0.0001). Statistical significance was determined by one-way ANOVA with Dunnett's multiple comparison test. Data are shown as mean ± SD in **b, d**. Source data are provided as a Source Data file.

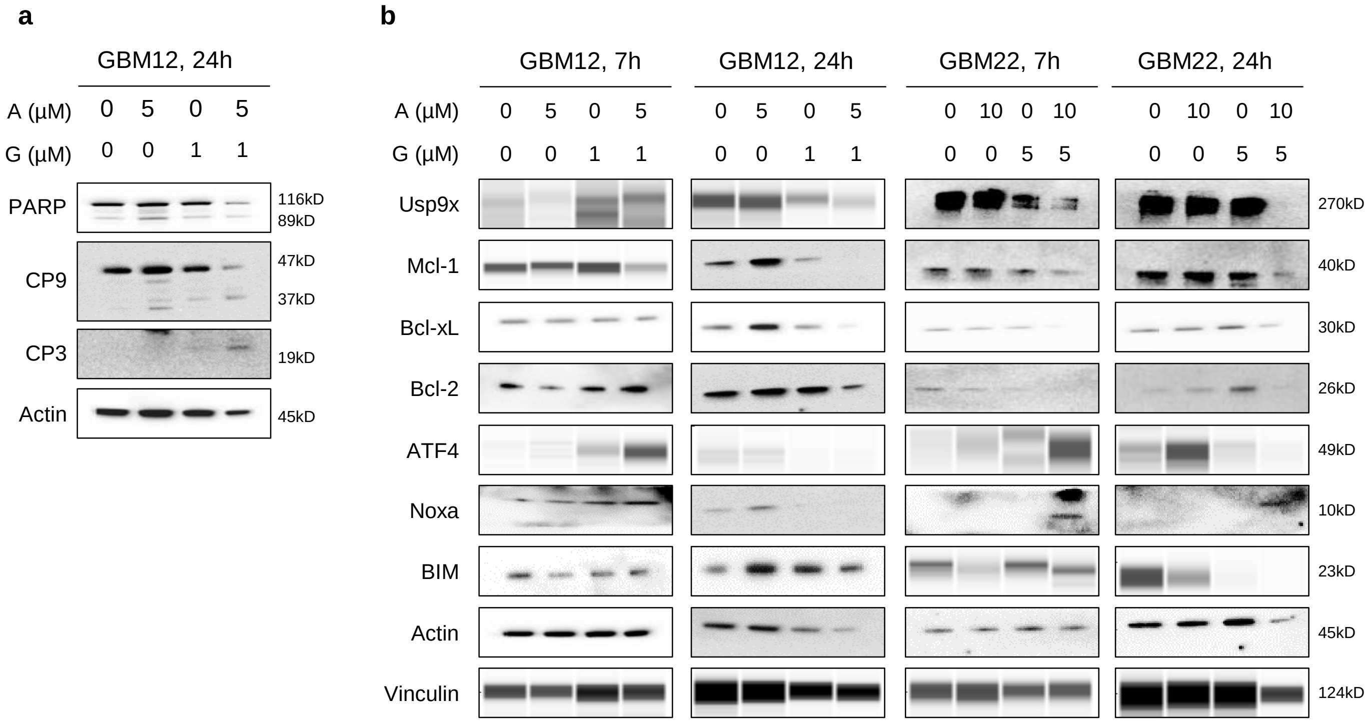

a
GBM12, 24h
A (µM) 0 5 0 5
G (µM) 0 0 1 1
PARP
CP9
CP3
Actin
116kD
89kD
47kD
37kD
19kD
45kD
b
GBM12, 7h
GBM12, 24h
GBM22, 7h
GBM22, 24h
A (µM) 0 5 0 5 0 5 0 5 0 10 0 10 0 10 0 10
G (µM) 0 0 1 1 0 0 1 1 0 0 5 5 0 0 5 5
Usp9x
Mcl-1
Bcl-xL
Bcl-2
ATF4
Noxa
BIM
Actin
Vinculin
270kD
40kD
30kD
26kD
49kD
10kD
23kD
45kD
124kD

Supplementary Figure 9: The combination treatment of gamitrinib and alisertib elicits an integrated stress response and modulates the expression of Bcl-2 family members. a Standard western blots of GBM12 cell lysates treated with alisertib, GTPP or combination of both for 24h for the indicated proteins. **b** Standard western blots or protein capillary electrophoresis of GBM12 and GBM22 cell lysates treated with alisertib, GTPP or combination of both for 7h and 24h for the indicated proteins.

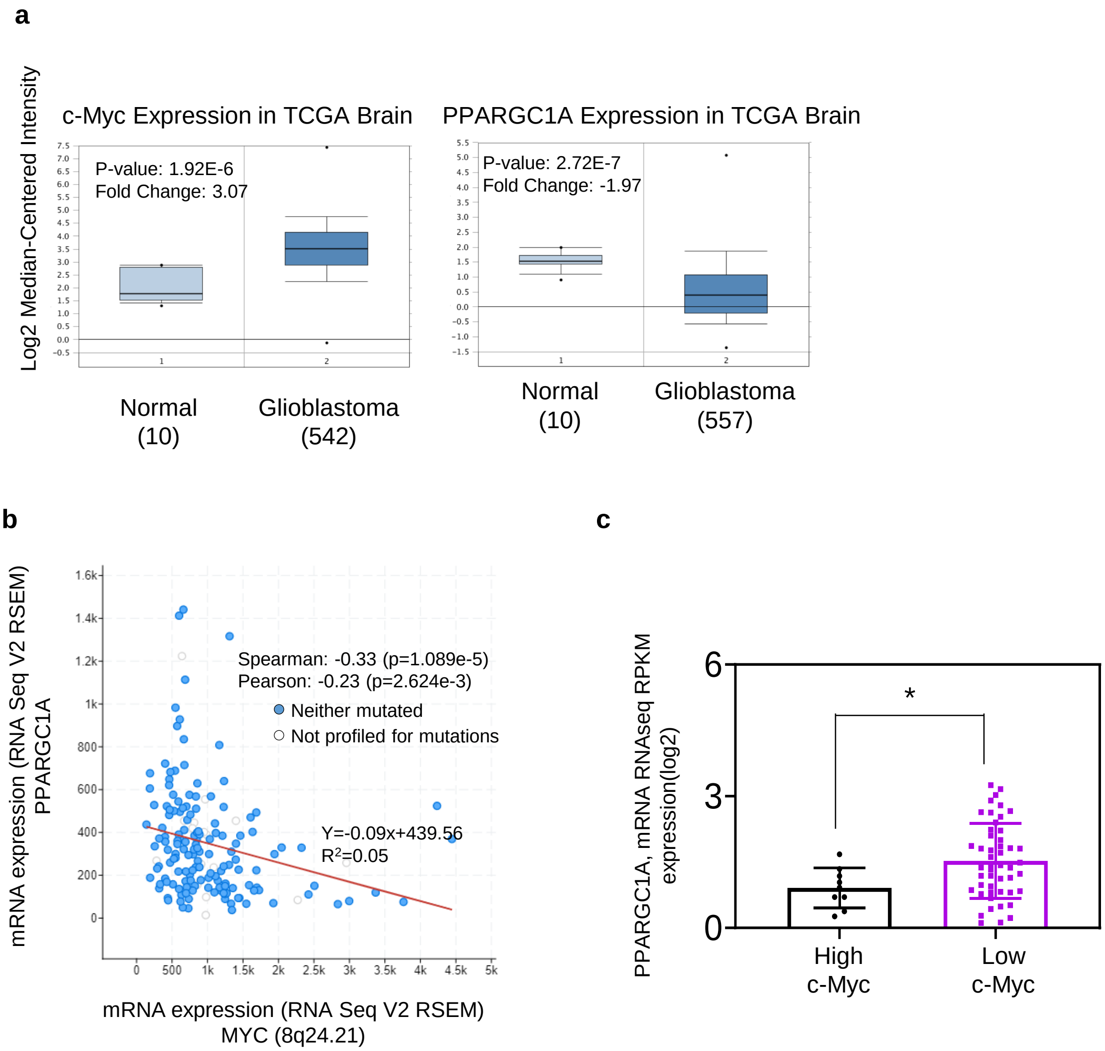

a
Log2 Median-Centered Intensity
c-Myc Expression in TCGA Brain
P-value: 1.92E-6
Fold Change: 3.07
Normal
(10)
Glioblastoma
(542)
PPARGC1A Expression in TCGA Brain
P-value: 2.72E-7
Fold Change: -1.97
Normal
(10)
Glioblastoma
(557)
b
mRNA expression (RNA Seq V2 RSEM)
PPARGC1A
Spearman: -0.33 (p=1.089e-5)
Pearson: -0.23 (p=2.624e-3)
Neither mutated
Not profiled for mutations
Y=-0.09x+439.56
R2=0.05
mRNA expression (RNA Seq V2 RSEM)
MYC (8q24.21)
c
PPARGC1A, mRNA RNAseq RPKM
expression(log2)
*
High
c-Myc
Low
c-Myc

Supplementary Figure 10: The correlation of mRNA levels between c-Myc and PPARGC1A expression in glioblastoma datasets. a Shown are the mRNA levels of c-Myc and PPARGC1A expression in the TCGA glioblastoma database (normal brain tissue vs. glioblastoma). Data was extracted from oncomine.org. Middle lines in boxplot: median; box ranges: upper: 75th percentile; lower: 25th percentile; error bars: 10th and 90th percentile; number of samples: normal n = 10, glioblastoma n=557. **b** Shown are the correlation of mRNA levels of c-Myc vs PPARGC1A expression in glioblastoma. Data extracted from cBioPortal (https://www.cbioportal.org). Statistical analysis was determined by p-value (two-tailed) Spearman and Pearson's rank correlation. **c** Shown are the correlation of mRNA levels of c-Myc vs PPARGC1A expression in the PDX GBM Mayo Clinic database (n= 9 in high c-Myc group and n = 49 in low c-Myc group) (*p=0.0396). Statistical significance was determined by two-tailed student's t-test in **c.** Data are shown as mean ± SD. Data extracted from cBioPortal (https://www.cbioportal.org). Source data are provided as a Source Data file.

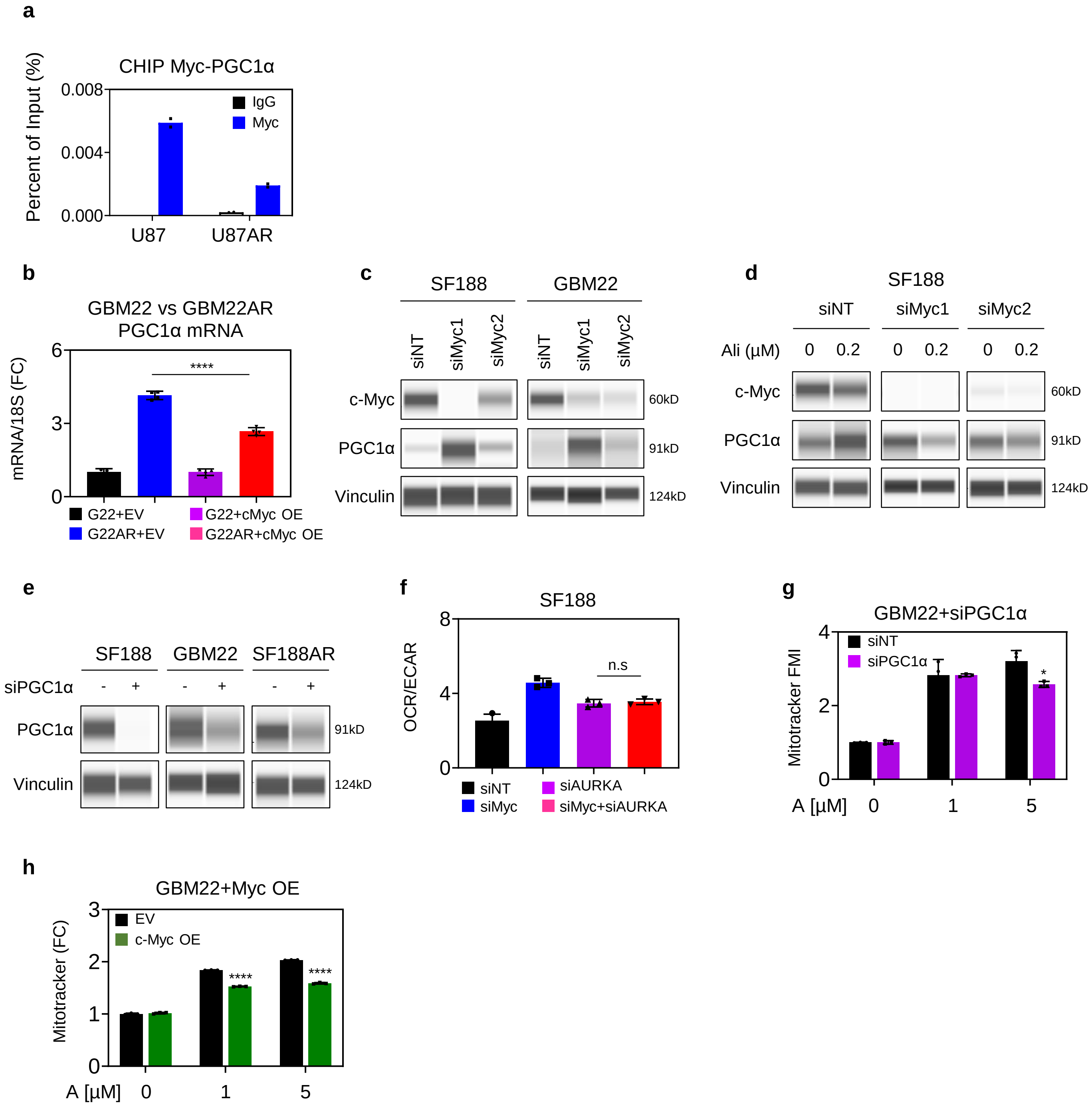
a
CHIP Myc-PGC1α
Percent of Input (%)
0.008
0.004
0.000
IgG
Myc
U87
U87AR
b
GBM22 vs GBM22AR
PGC1α mRNA
mRNA/18S (FC)
6
3
0
****
G22+EV
G22AR+EV
G22+cMyc OE
G22AR+cMyc OE
c
SF188
GBM22
siNT
siMyc1
siMyc2
c-Myc
PGC1α
Vinculin
60kD
91kD
124kD
d
SF188
siNT
siMyc1
siMyc2
Ali (µM)
0
0.2
c-Myc
PGC1α
Vinculin
60kD
91kD
124kD
e
SF188
GBM22
SF188AR
siPGC1α
-
+
PGC1α
Vinculin
91kD
124kD
f
SF188
OCR/ECAR
8
4
0
n.s
siNT
siMyc
siAURKA
siMyc+siAURKA
g
GBM22+siPGC1α
Mitotracker FMI
4
2
0
siNT
siPGC1α
*
A [µM]
0
1
5
h
GBM22+Myc OE
Mitotracker (FC)
3
2
1
0
EV
c-Myc OE
****
****
A [µM]
0
1
5

Supplementary Figure 11: Aurora kinase A inhibition affects c-Myc and PGC1A protein levels. a Parental or chronically alisertib treated U87 cells were subjected to CHIP with an IgG as a negative control or a c-Myc specific antibody. The PGC1α region was amplified (n=2 independent samples). **b** Real time PCR analysis of PGC1α mRNA level of GBM22 and GBM22AR cells infected with empty vector or c-Myc adenovirus (n=3 in GBM22-EV, n=4 in GBM22AR-EV, GBM22-cMyc OE, and GBM22AR-cMyc OE independent samples) (****p<0.0001). **c** SF188 and GBM22 cells were transfected with non-targeting siRNA or two specific siRNAs targeting c-Myc and the whole cell lysates were subjected to protein capillary electrophoresis. **d** SF188 cells were transfected with non-targeting siRNA or two specific siRNAs targeting c-Myc, treated with the indicated concentration of alisertib for 24h, and the whole cell lysates were subjected to protein capillary electrophoresis. **e** Shown are protein capillary electrophoresis of SF188, GBM22, and SF188AR cells transfected with non-targeting siRNA or specific siRNA targeting PGC1α. **f** SF188 cells were transfected with non-targeting siRNA or specific siRNAs targeting c-Myc, AURKA, or the combination of both and were analyzed in the context of a glycolysis stress assay on a Seahorse XFe24 extracellular flux analyzer. Extracellular acidification rate (ECAR) is recorded at baseline, after injection of Glucose (G), Oligomycin (OM), and 2-DG. The graphs show OCR/ECAR level (n=3 independent samples) (n.s: not significant). **g** GBM22 cells were transfected with non-targeting siRNA or specific siRNA targeting PGC1α, treated with increasing concentration of alisertib for 48h, labeled with mitotracker, and analyzed by flow cytometry. Shown is the quantification (n=3 independent samples) (*p=0.0218). **h** GBM22 cells were infected with empty vector or c-Myc adenovirus, treated with increasing concentration of alisertib for 48h, labeled with mitotracker, and analyzed by flow cytometry. Shown is the quantification (n=3 independent samples) (****p<0.0001). Statistical significance was assessed by two-tailed student's t-test. Data are shown as mean ± SD in **b, f-h**. Source data are provided as a Source Data file.

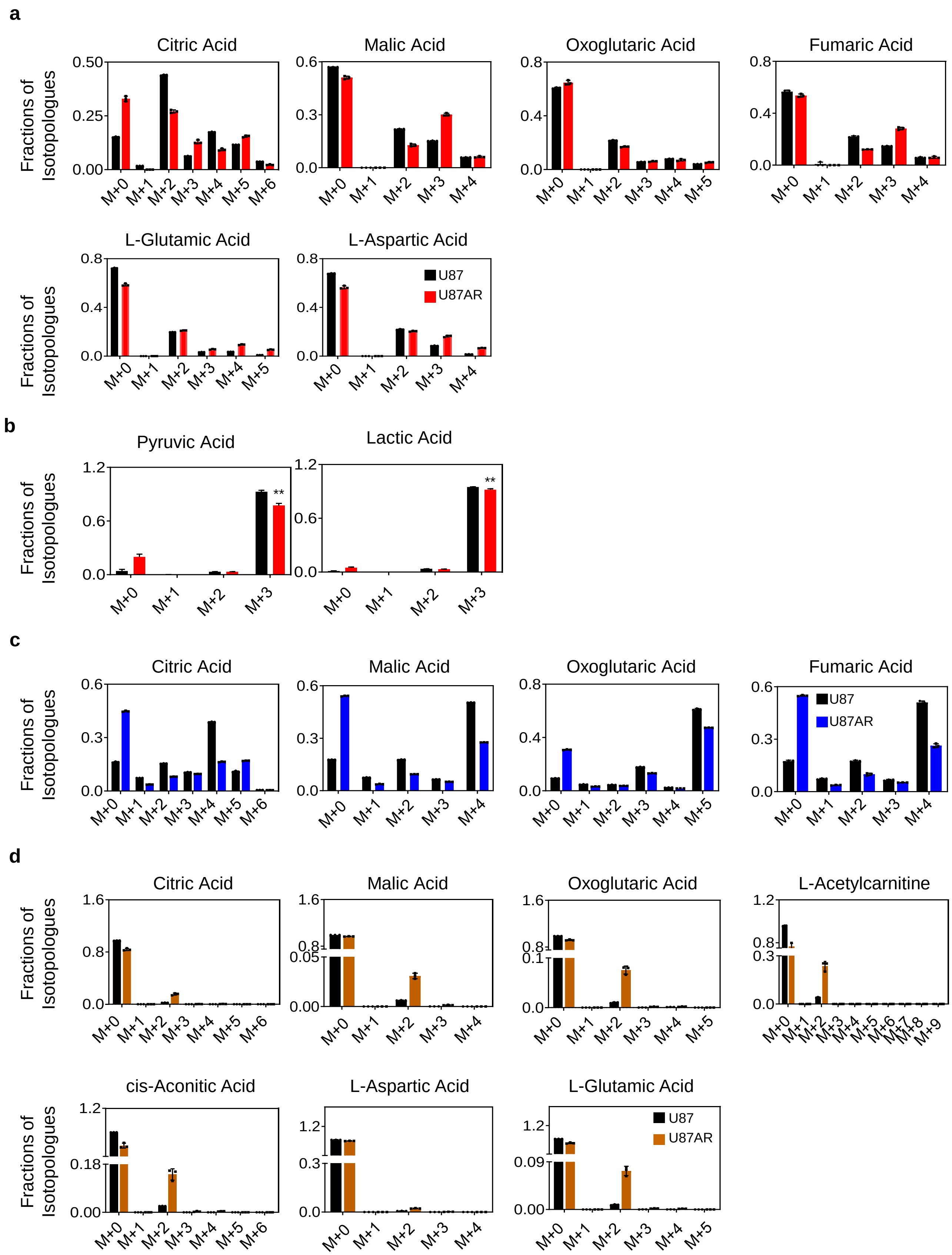
a
Citric Acid
Malic Acid
Oxoglutaric Acid
Fumaric Acid
L-Glutamic Acid
L-Aspartic Acid
U87
U87AR
Fractions of Isotopologues
b
Pyruvic Acid
Lactic Acid
**
c
Citric Acid
Malic Acid
Oxoglutaric Acid
Fumaric Acid
U87
U87AR
d
Citric Acid
Malic Acid
Oxoglutaric Acid
L-Acetylcarnitine
cis-Aconitic Acid
L-Aspartic Acid
L-Glutamic Acid
U87
U87AR

Supplementary Figure 12: AURKA inhibition impacts central carbon metabolism resulting in enhanced labeling of TCA cycle metabolites by long chain fatty acids. For **a, b** Parental or chronically alisertib treated U87 cells were cultured in DMEM media containing 25 mM U-^{13}C glucose, 4mM glutamine, and 10% dialyzed FBS, and subjected to LC/MS analysis. Shown are relative percentages of the isotopologues for each metabolite (n=3 independent samples) (Lactic Acid: **p=0.0066, Pyruvic Acid: **p=0.001). **c** Parental or chronically alisertib treated U87 cells were cultured in DMEM media containing 25 mM glucose, 4mM U-^{13}C glutamine and 10% dialyzed FBS and subjected to LC/MS analysis. Shown are relative percentages of the isotopologues for each metabolite (n=3 independent samples). **d** Parental or chronically alisertib treated U87 cells were cultured in DMEM media containing 5 mM glucose, 1mM glutamine, 100 μM U-^{13}C palmitic acid, and 10% dialyzed FBS, and subjected to LC/MS analysis. Shown are relative percentages of the isotopologues for each metabolite (n=3 independent samples). Data are shown as mean ± SD in **a-d**. Source data are provided as a Source Data file.

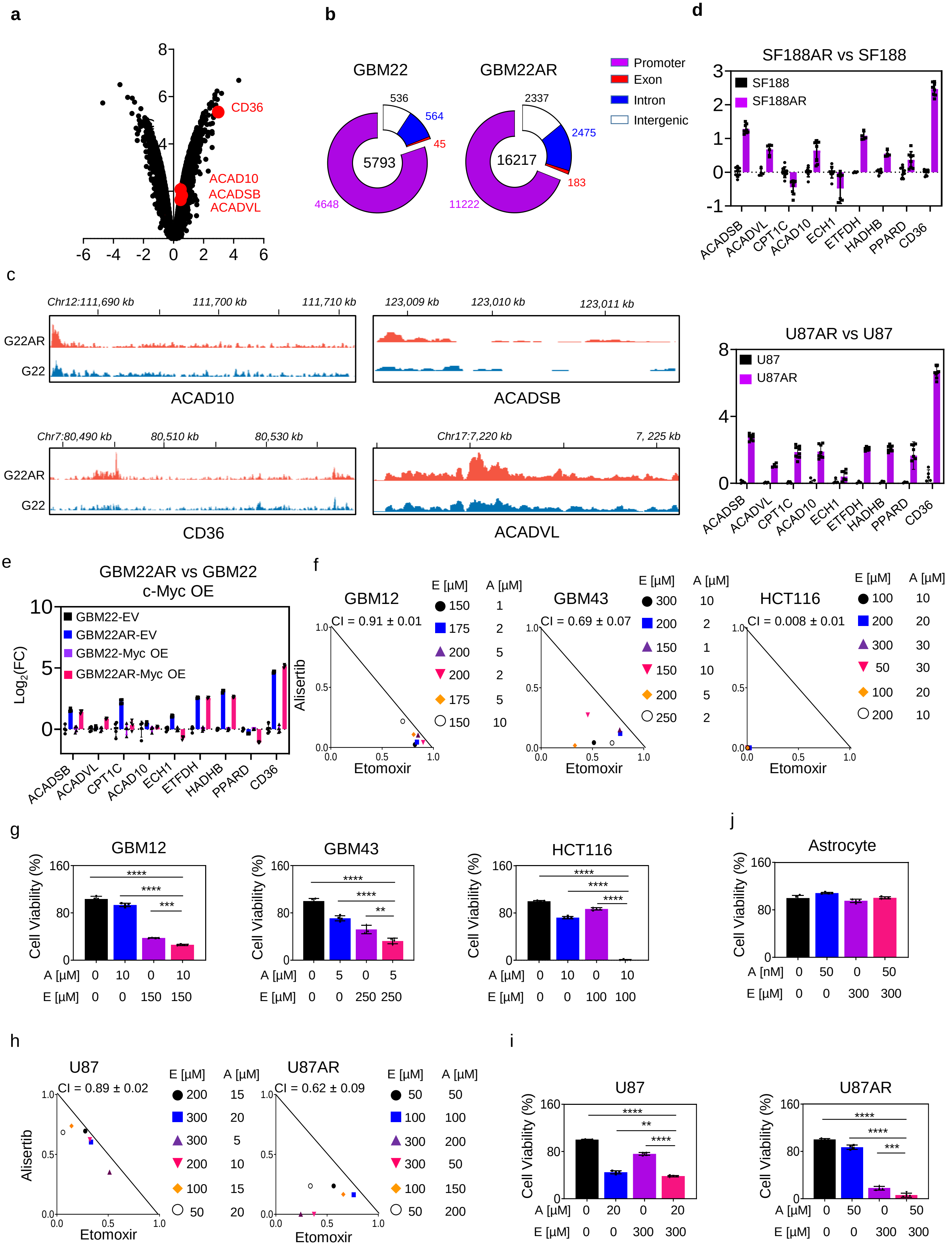
a
CD36
ACAD10
ACADSB
ACADVL
b
GBM22
GBM22AR
536
564
45
5793
4648
2337
2475
16217
183
11222
Promoter
Exon
Intron
Intergenic
c
Chr12:111,690 kb
111,700 kb
111,710 kb
G22AR
G22
ACAD10
123,009 kb
123,010 kb
123,011 kb
ACADSB
Chr7:80,490 kb
80,510 kb
80,530 kb
CD36
Chr17:7,220 kb
7, 225 kb
ACADVL
d
SF188AR vs SF188
SF188
SF188AR
U87AR vs U87
U87
U87AR
ACADSB
ACADVL
CPT1C
ACAD10
ECH1
ETFDH
HADHB
PPARD
CD36
e
GBM22AR vs GBM22
c-Myc OE
GBM22-EV
GBM22AR-EV
GBM22-Myc OE
GBM22AR-Myc OE
Log2(FC)
f
GBM12
CI = 0.91 ± 0.01
GBM43
CI = 0.69 ± 0.07
HCT116
CI = 0.008 ± 0.01
Alisertib
Etomoxir
E [µM]
A [µM]
g
GBM12
GBM43
HCT116
Cell Viability (%)
j
Astrocyte
A [nM]
h
U87
CI = 0.89 ± 0.02
U87AR
CI = 0.62 ± 0.09
i
U87
U87AR

Supplementary Figure 13: Aurora kinase A inhibition regulates lipid metabolism. a Shown is a volcano plot (derived from a transcriptome analysis) of chronically alisertib treated GBM22 cells with its parental cells. Highlighted is the mRNA level of fatty acid (CD36, ACAD10, ACADSB, ACADVL). FC: fold change, p: p-value (n=2 independent samples). For **b, c** ChIP-sequencing (H3K27ac) was performed in parental or chronically alisertib treated GBM22 cells. Shown is annotated peak analysis of GBM22 parental and GBM22 AR cells. Shown are the respective tracks around the ACAD10, ACADSB, CD36, and ACADVL locus in **c**. **d** Real time PCR analysis of genes related to fatty acid oxidation mRNA levels of parental or chronically alisertib treated SF188 and U87 cells (n=4 independent samples). **e** Real time PCR analysis of genes related to fatty acid oxidation mRNA levels of parental or chronically alisertib treated GBM22 cells infected with empty vector or c-Myc adenovirus (n=4 independent samples). For **f, g** GBM12, GBM43, and HCT116 cells were treated with alisertib, etomoxir or the combination of both for 72h, and cellular viability was analyzed. Shown are the isobolograms. The quantification is shown in **g** (n=4 in GBM12 and HCT116, GBM43: n=3 in etomoxir, n=4 in DMSO, alisertib, and combination independent samples) (**p=0.0011, ***p=0.0002, ****p<0.0001). For **h, i** Parental or chronically alisertib treated U87 cells were treated with alisertib, etomoxir or the combination of both for 72h, and cellular viability was analyzed. Shown are the isobolograms. The quantification is shown in **i** (n=4 independent samples) (**p=0.001, ***p=0.0006, ****p<0.0001). **j** Shown is the quantification of astrocyte cells treated with alisertib, etomoxir or the combination of both for 72h, and cellular viability was performed (n=4 independent samples). Statistical significance was determined by one-way ANOVA with Dunnett's multiple comparison test. Data are shown as mean ± SD in **d, e, g, I, j**. Source data are provided as a Source Data file.

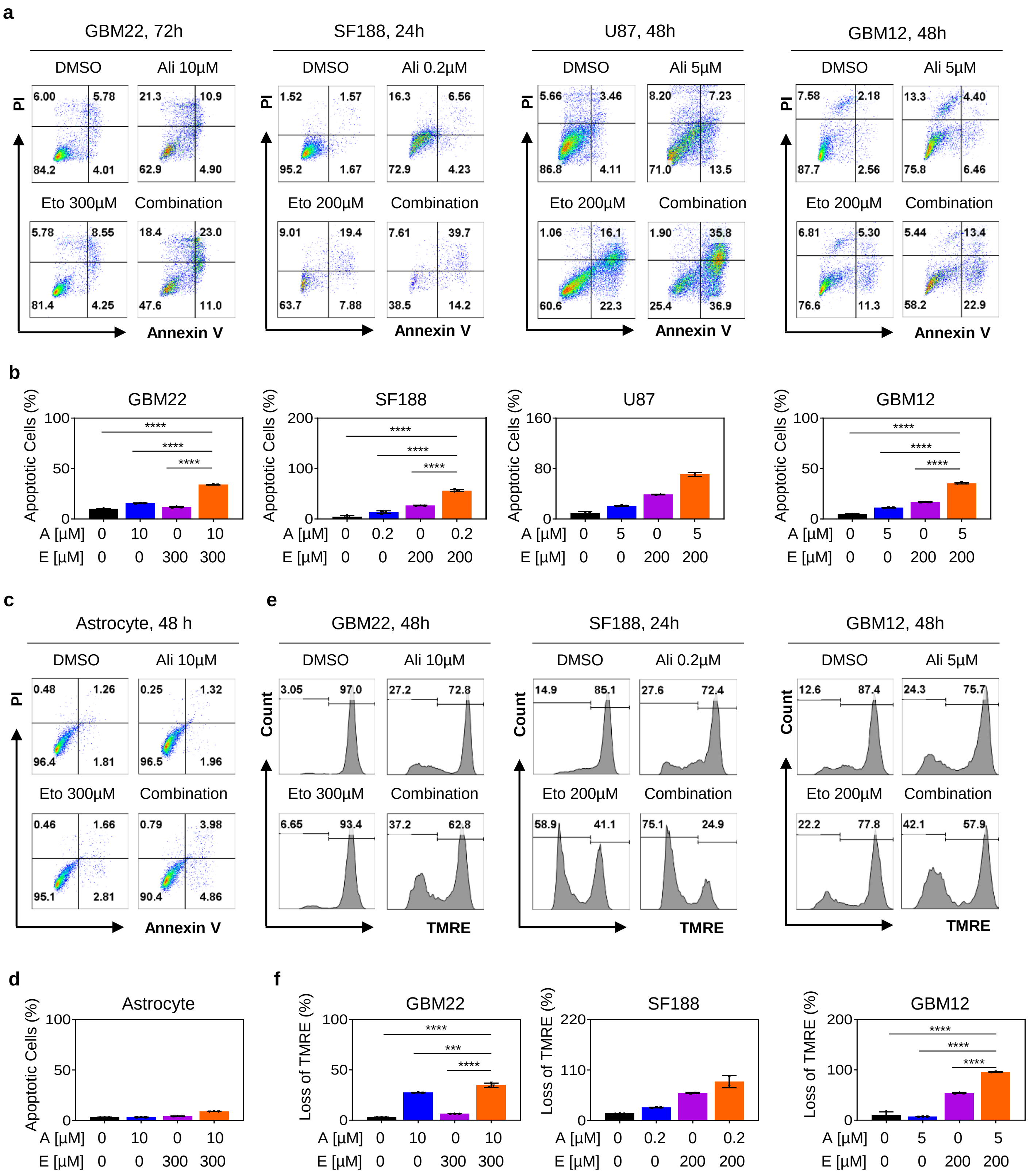
a
GBM22, 72h
SF188, 24h
U87, 48h
GBM12, 48h
DMSO
Ali 10µM
Ali 0.2µM
Ali 5µM
Eto 300µM
Eto 200µM
Combination
PI
Annexin V
6.00 5.78 84.2 4.01
21.3 10.9 62.9 4.90
5.78 8.55 81.4 4.25
18.4 23.0 47.6 11.0
1.52 1.57 95.2 1.67
16.3 6.56 72.9 4.23
9.01 19.4 63.7 7.88
7.61 39.7 38.5 14.2
5.66 3.46 86.8 4.11
8.20 7.23 71.0 13.5
1.06 16.1 60.6 22.3
1.90 35.8 25.4 36.9
7.58 2.18 87.7 2.56
13.3 4.40 75.8 6.46
6.81 5.30 76.6 11.3
5.44 13.4 58.2 22.9
b
GBM22
SF188
U87
GBM12
Apoptotic Cells (%)
A [µM]
E [µM]
****
c
Astrocyte, 48 h
0.48 1.26 96.4 1.81
0.25 1.32 96.5 1.96
0.46 1.66 95.1 2.81
0.79 3.98 90.4 4.86
e
GBM22, 48h
SF188, 24h
GBM12, 48h
Count
TMRE
3.05 97.0
27.2 72.8
6.65 93.4
37.2 62.8
14.9 85.1
27.6 72.4
58.9 41.1
75.1 24.9
12.6 87.4
24.3 75.7
22.2 77.8
42.1 57.9
d
Astrocyte
f
Loss of TMRE (%)
***

Supplementary Figure 14: The combination treatment of etomoxir and alisertib facilitates an enhanced cell death with apoptotic features. For **a, b** GBM22, SF188, U87, and GBM12 cells were treated with alisertib, etomoxir or combination of both, labeled with Annexin/PI dye, and analyzed by flow cytometry. Quantification is shown in **b** (n=3 in GBM22, SF188, and GBM12; U87: n=2 in etomoxir and combination, n=3 in DMSO and alisertib independent samples) (****p<0.0001). For **c, d** Astrocyte cells were treated with alisertib, etomoxir or combination of both, labeled with Annexin/PI dye, and analyzed by flow cytometry. Quantification is shown in **d** (n=3 independent samples). For **e, f** GBM22, SF188, and GBM12 cells were treated with alisertib, etomoxir or combination of both, labeled with TMRE dye, and analyzed by flow cytometry. Quantification is shown in **f** (n=3 in GBM22 and GBM12; SF188: n=2 in etomoxir and combination, n=3 in DMSO and alisertib independent samples) (***p=0.0002, ****p<0.0001). Statistical significance was determined by one-way ANOVA with Dunnett's multiple comparison test. Data are shown as mean ± SD in **b, d, f**. Source data are provided as a Source Data file.

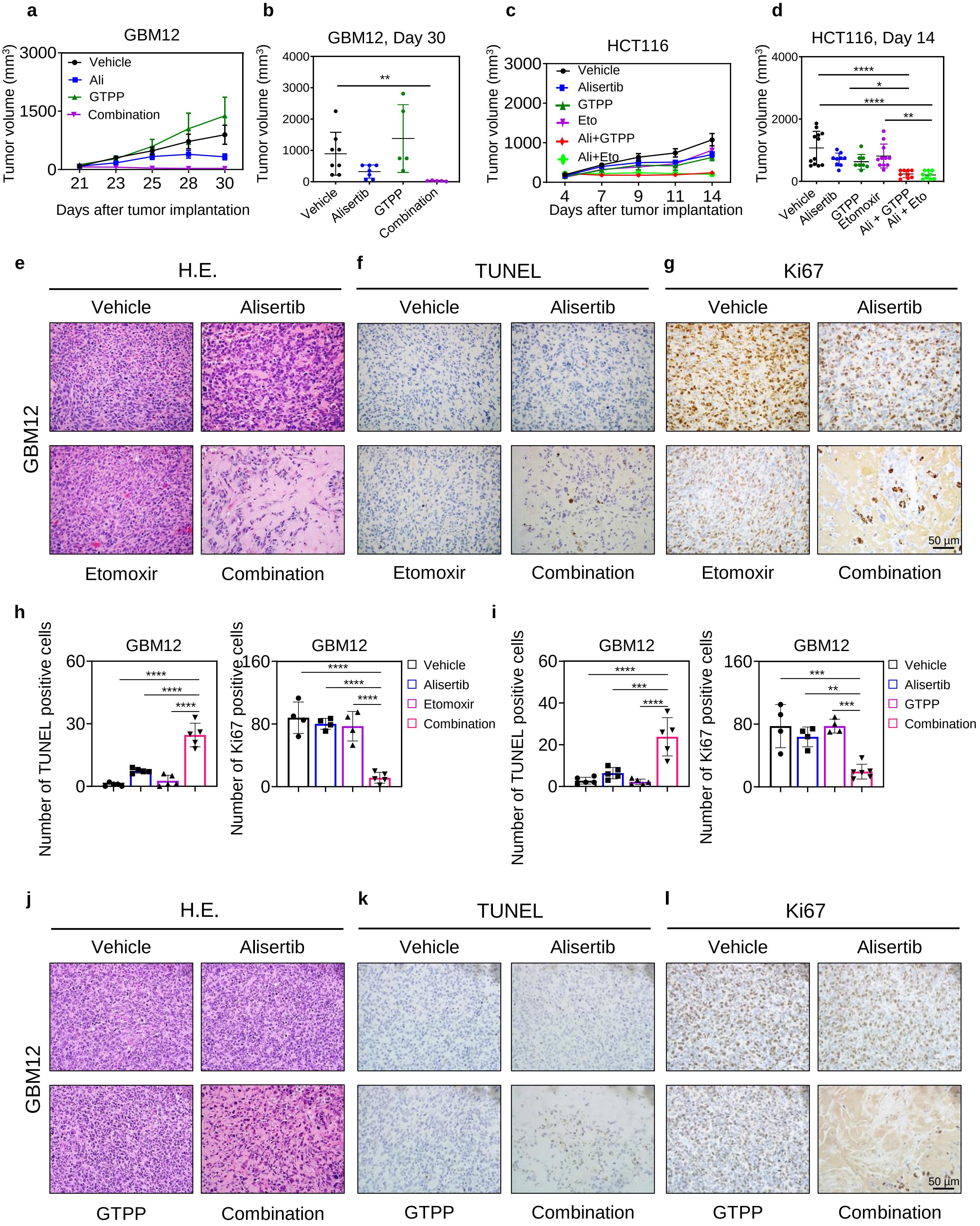
a
GBM12
Tumor volume (mm3)
3000
1500
0
Vehicle
Ali
GTPP
Combination
21
23
25
28
30
Days after tumor implantation
b
GBM12, Day 30
Tumor volume (mm3)
4000
3000
2000
1000
0
**
Vehicle
Alisertib
GTPP
Combination
c
HCT116
Tumor volume (mm3)
3000
2000
1000
0
Vehicle
Alisertib
GTPP
Eto
Ali+GTPP
Ali+Eto
4
7
9
11
14
Days after tumor implantation
d
HCT116, Day 14
Tumor volume (mm3)
4000
2000
0
****
*
****
**
Vehicle
Alisertib
GTPP
Etomoxir
Ali + GTPP
Ali + Eto
e
H.E.
Vehicle
Alisertib
GBM12
Etomoxir
Combination
f
TUNEL
Vehicle
Alisertib
Etomoxir
Combination
g
Ki67
Vehicle
Alisertib
50 µm
Etomoxir
Combination
h
GBM12
Number of TUNEL positive cells
60
30
0
****
****
****
GBM12
Number of Ki67 positive cells
160
80
0
****
****
****
Vehicle
Alisertib
Etomoxir
Combination
i
GBM12
Number of TUNEL positive cells
60
40
20
0
****
***
****
GBM12
Number of Ki67 positive cells
160
80
0
***
**
***
Vehicle
Alisertib
GTPP
Combination
j
H.E.
Vehicle
Alisertib
GBM12
GTPP
Combination
k
TUNEL
Vehicle
Alisertib
GTPP
Combination
l
Ki67
Vehicle
Alisertib
50 µm
GTPP
Combination

Supplementary Figure 15: The combination treatment of inhibitors of mitochondrial metabolism and alisertib results in an enhanced reduction of tumor growth. a GBM12 cells were implanted into the subcutis of immunocompromised Nu/Nu mice. After the tumors were established, randomization occurred to form four treatment groups: vehicle, alisertib (30 mg/kg), GTPP (5 mg/kg), and combination treatment of both. Mice were treated three times per week. Shown are the tumor volumes over time. **b** The graph shows tumor volume on the last day of the experiment in **a** (n=5 in GTPP and combination, n=7 in alisertib, n=8 in vehicle independent tumors) (**p=0.0093). **c** HCT116 cells were implanted into the subcutis of immunocompromised Nu/Nu mice. After the tumors were established, randomization occurred to form six treatment groups: vehicle, alisertib (30 mg/kg), etomoxir (20 mg/kg), GTPP (5 mg/kg), alisertib+etomoxir or alisertib+GTPP. Shown are the tumor volumes over time. **d** The graph shows the body weights of six treatment groups in **c** (n=8 in GTPP, n=9 in alisertib, alisertib+GTPP, alisertib+etomoxir, n=11 in etomoxir, n=12 in vehicle independent tumors) (*p=0.0455, **p=0.0019, ****p<0.0001). For **e-h** H&E, TUNEL, and Ki67 stainings of the vehicle, alisertib, etomoxir, or the combination treatment in experiment in Fig. 7**a**. Quantification of TUNEL and Ki67 positive cells is shown in **h** (n TUNEL: n=5; Ki67: n=4 in Vehicle, alisertib, etomoxir, n=5 in combination independent high-power field microscopy) (****p<0.0001)**.** For **i-l** H&E, TUNEL, and Ki67 stainings of the vehicle, alisertib, GTPP, or the combination treatment in experiment in Fig. 7**c**. Quantification of TUNEL and Ki67 positive cells is shown in **i** (TUNEL: n=5; Ki67: n=4 in Vehicle, alisertib, GTPP, n=6 in combination independent high-power field microscopy) (**p= 0.0018, TUNEL: ***p=0.0001, Ki67:***p=0.0002, ****p<0.0001). Scale bar: 50 µm. Statistical significance was determined by one-way ANOVA with Dunnett's multiple comparison test. Data are shown as mean ± SEM in **a, c** and as mean ± SD in **b, d, h, i**. Source data are provided as a Source Data file.

a

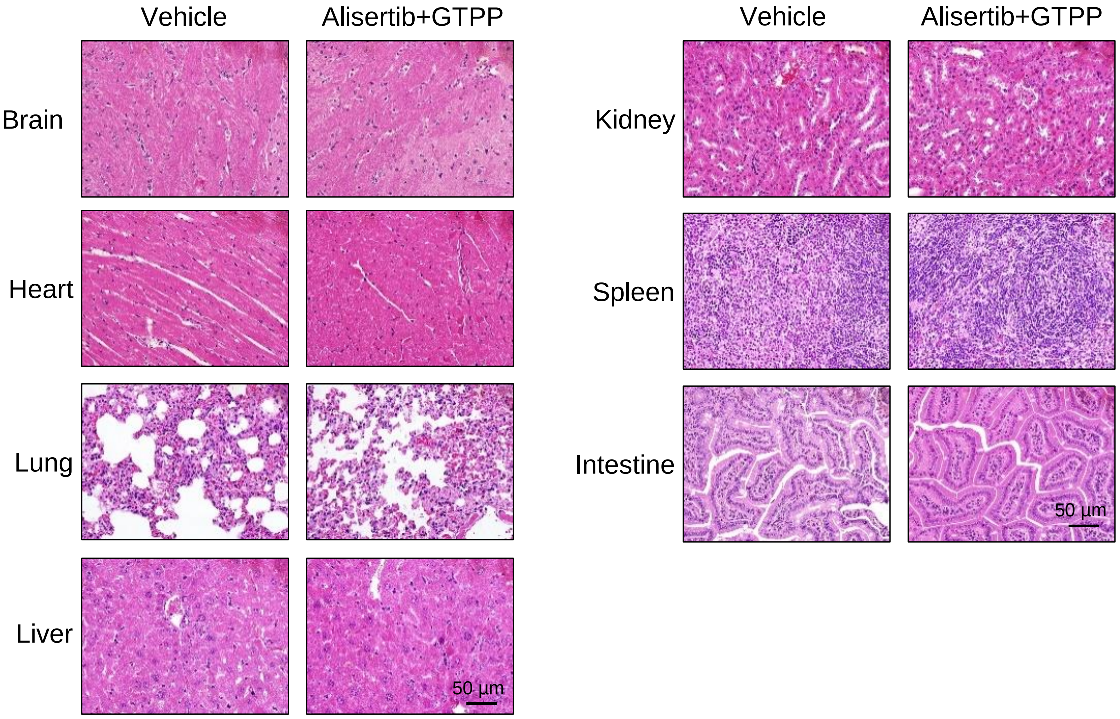

Vehicle
Alisertib+GTPP
Brain
Heart
Lung
Liver
50 µm
Vehicle
Alisertib+GTPP
Kidney
Spleen
Intestine
50 µm

b

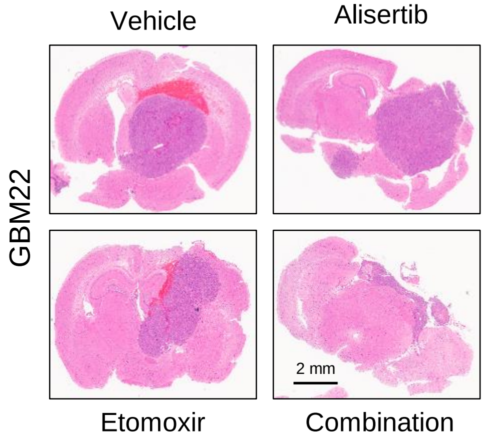

Vehicle
Alisertib
GBM22
2 mm
Etomoxir
Combination

Supplementary Figure 16: The combination treatment of alisertib and gamitrinib (GTPP) does not induce organ toxicity. a H&E staining of the vehicle, alisertib, GTPP, or combination treatment in different organ systems related to the GBM12 xenografts in Fig. S15**a**. Scale bar: 50 µm. **b** The brain tumors from the experiment in Fig. 7**h** were fixed and stained with H&E. Scale bar: 2 mm.

a

PI/Annexin V Gating Strategy

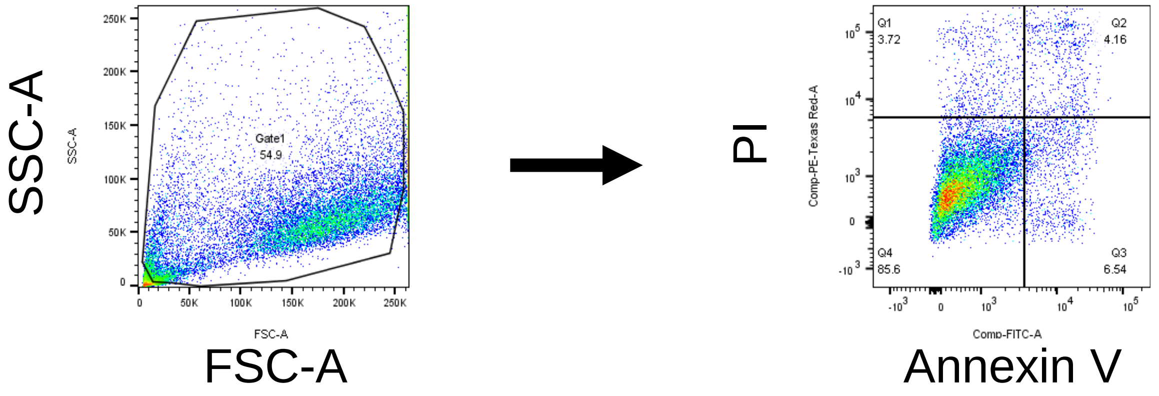

b

TMRE Gating Strategy

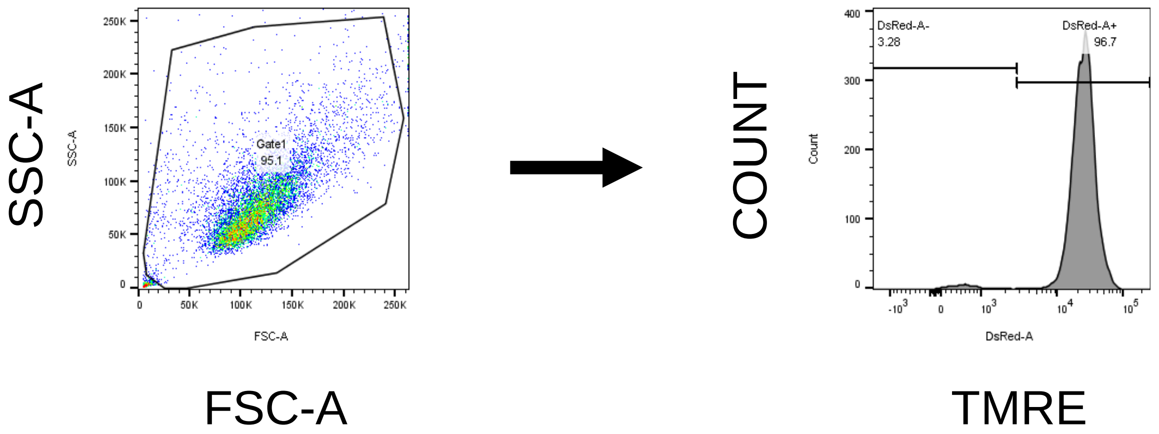

Supplementary Figure 17: Sequential gating strategies used for flow cytometry. **a**. Gating strategy to analyse PI/Annexin V in Supplementary Figs. 2a, 2c, 8a, 14a, 14c. **b**. Gating strategy to analyse TMRE in Supplementary Figs. 8c, 14e.

Supplementary Table 1: Primer sequences for real time PCR and chromatin immunoprecipitation qPCR

Name	Sequence
qPCR primer hGLUT1_F	TTGCAGGCTTCTCCAACTGGAC
qPCR primer hGLUT1_R	CAGAACCAGGAGCACAGTGAAG
qPCR primer hHK2_F	CGGCCGTGCTACAATAGG
qPCR primer hHK2_R	CTCGGGATCATGTGAGGG
qPCR primer: hLDHA_F	GGATCTCCAACATGGCAGCCTT
qPCR primer: hLDHA_R	AGACGGCTTTCTCCCTCTTGCT
qPCR primer: hMyc_F	CCTGGTGCTCCATGAGGAGAC
qPCR primer: hMyc_R	CAGACTCTGACCTTTTGCCAGG
qPCR primer: hPPARGC1A (PGC1α)_F	CCAAAGGATGCGCTCTCGTTCA
qPCR primer: hPPARGC1A (PGC1α)_R	CGGTGTCTGTAGTGGCTTGACT
qPCR primer: hPDK4_F	AGGTGGAGCATTTCTCGCGCTA
qPCR primer: hPDK4_R	GAATGTTGGCGAGTCTCACAGG
qPCR primer: hACADSB_F	GCCACCTATTTGCCTCAGCTCA
qPCR primer: hACADSB_R	GCTCAGCACTGCTGATCCACAT
qPCR primer: hACADVL_F	TAGGAGAGGCAGGCAAACAGCT
qPCR primer: hACADVL_R	CACAGTGGCAAACTGCTCCAGA
qPCR primer: hCPT1C_F	TGCCATGTCGTTCCATTCTCCC
qPCR primer: hCPT1C_R	GCCGACTCATAAGTCAGGCAGA
qPCR primer: hACAD10_F	GCCTCTTCAGATGCCACCAACA
qPCR primer: hACAD10_R	CACAGAGTTGGCAACGAGGATC
qPCR primer: hECH1_F	CGATACCAGGAGACCTTCAACG
qPCR primer: hECH1_R	GGAAGAAAGCATCCTGGGCACA
qPCR primer: hETFDH_F	GGAAACACCATCCTAGCATTCGG
qPCR primer: hETFDH_R	CCACCAGGAAAGGTGAGTTTTGG
qPCR primer: hHADHB_F	CACAGTCTAGCCAAGAAGGCAC
qPCR primer: hHADHB_R	CATCTGCTCCAGTGAGGAAGGA
qPCR primer: hPPARD_F	GGCTTCCACTACGGTGTTCATG
qPCR primer: hPPARD_R	CTGGCACTTGTTGCGGTTCTTC
qPCR primer: hCD36_F	CAGGTCAACCTATTGGTCAAGCC
qPCR primer: hCD36_R	GCCTTCTCATCACCAATGGTCC
ChIP qPCR primer: HK2 promoter region_F	GATTGCCTCGCATCTGC
ChIP qPCR primer: HK2 promoter region_R	TTTGCCAGAGCCCAGC
ChIP qPCR primer: HK2 exon 1_F	CACATTGTTGCATGAAACTCC
ChIP qPCR primer: HK2 exon 1_R	GACCTCTCCGATTCACAGG
ChIP qPCR primer: PGC1A promoter_F	CTGGGTGTGCGTCTGTTTG
ChIP qPCR primer: PGC1A promoter_R	CGGCGTGGTCTGATTTAGTG

HA-Aurora-D274N_F	TACTGACCACCCAAAATTTGCAAT TTTAAGCTCTCCAGCT
HA-Aurora-D274N_R	AGCTGGAGAGCTTAAAATTGCAA A TTTTGGGTGGTCAGTA
