# Supplementary material for: Aurora kinase A inhibition reverses the Warburg effect and elicits unique metabolic vulnerabilities in glioblastoma
Source: Nat Commun. 2021 Sep 1;12:5203. doi: 10.1038/s41467-021-25501-x (PMC8410792; doi:10.1038/s41467-021-25501-x)
Supplement: Supplementary file 3 — Reporting summary [file 41467_2021_25501_MOESM3_ESM.pdf]

nature portfolio

Corresponding author(s): Markus Siegelin

Last updated by author(s): Jul 27, 2021

Reporting Summary

Nature Portfolio wishes to improve the reproducibility of the work that we publish. This form provides structure for consistency and transparency in reporting. For further information on Nature Portfolio policies, see our Editorial Policies and the Editorial Policy Checklist.

Statistics

For all statistical analyses, confirm that the following items are present in the figure legend, table legend, main text, or Methods section.

n/a	Confirmed	
☐	☒	The exact sample size (*n*) for each experimental group/condition, given as a discrete number and unit of measurement
☐	☒	A statement on whether measurements were taken from distinct samples or whether the same sample was measured repeatedly
☐	☒	The statistical test(s) used AND whether they are one- or two-sided<br>*Only common tests should be described solely by name; describe more complex techniques in the Methods section.*
☒	☐	A description of all covariates tested
☒	☐	A description of any assumptions or corrections, such as tests of normality and adjustment for multiple comparisons
☐	☒	A full description of the statistical parameters including central tendency (e.g. means) or other basic estimates (e.g. regression coefficient) AND variation (e.g. standard deviation) or associated estimates of uncertainty (e.g. confidence intervals)
☐	☒	For null hypothesis testing, the test statistic (e.g. *F*, *t*, *r*) with confidence intervals, effect sizes, degrees of freedom and *P* value noted<br>*Give P values as exact values whenever suitable.*
☒	☐	For Bayesian analysis, information on the choice of priors and Markov chain Monte Carlo settings
☒	☐	For hierarchical and complex designs, identification of the appropriate level for tests and full reporting of outcomes
☐	☒	Estimates of effect sizes (e.g. Cohen's *d*, Pearson's *r*), indicating how they were calculated

Our web collection on statistics for biologists contains articles on many of the points above.

Software and code

Policy information about availability of computer code

Data collection	BD-LSRII was used to analyze flow cytometry; Illumina HiSeQ 4000instrument was used for CHIP-seq and ATAC-seq; Azure (C300) imaging system and Wes simple (protein capillary eletrophoresis instrument) (ProteinSimple, Inc.) was used for protein expression analysis.
Data analysis	FlowJo software version 10.7 was used for flow cytometric analysis of cell death, ImageJ/FIJI 1.53c was used for blot quantification and PLA experiment. Graphpad prism 8 was used for statistical analysis and graphical representation of the results. CHIP-seq and ATAC-seq analyses was performed by using useGalaxy flatform, utilizing bowtie2 (Version 2.4.2), macs2 (Version 2.1.1.20160309) and ucsc-wigtobigwig (Version 357), Integrated Genome Browser 9.1.8 and basepair software. ComboSyn, Inc was used to determine drug synergism.

For manuscripts utilizing custom algorithms or software that are central to the research but not yet described in published literature, software must be made available to editors and reviewers. We strongly encourage code deposition in a community repository (e.g. GitHub). See the Nature Portfolio guidelines for submitting code & software for further information.

Data

Policy information about availability of data

All manuscripts must include a data availability statement. This statement should provide the following information, where applicable:

- Accession codes, unique identifiers, or web links for publicly available datasets
- A description of any restrictions on data availability
- For clinical datasets or third party data, please ensure that the statement adheres to our policy

The raw and processed ATAC-sequencing, CHIP-sequencing, and microarray data generated in this study have been deposited in the Gene Expression Omnibus (GEO), https://www.ncbi.nlm.nih.gov/geo/, under accession code GSE161572, GSE161573, and GSE152612 respectively. Source data are provided with this paper.

Field-specific reporting

Please select the one below that is the best fit for your research. If you are not sure, read the appropriate sections before making your selection.

☒ Life sciences ☐ Behavioural & social sciences ☐ Ecological, evolutionary & environmental sciences

For a reference copy of the document with all sections, see nature.com/documents/nr-reporting-summary-flat.pdf

Life sciences study design

All studies must disclose on these points even when the disclosure is negative.

Sample size	A sample size of n=3-5 (in vitro) or n= 5-8 (in vivo) were used for statistical analysis. Sample sizes were determined based on minimum number of n required to reach statistical power.
Data exclusions	We have not excluded data from our statistical analysis.
Replication	All experiments were reproducible. The figure and table legends indicated the nature of the samples, whether they consitute technical or biological replicates. All in vitro experiments were performed independently at least twice with similar results.
Randomization	In vitro and in-vivo experiments the samples were randomly assigned to different groups (control vs. treated).
Blinding	For both in vitro as well as in vivo experiments blinding was not performed because the experiments were carried out by the same researchers.

Reporting for specific materials, systems and methods

We require information from authors about some types of materials, experimental systems and methods used in many studies. Here, indicate whether each material, system or method listed is relevant to your study. If you are not sure if a list item applies to your research, read the appropriate section before selecting a response.

Materials & experimental systems

n/a	Involved in the study	
☐	☒	Antibodies
☐	☒	Eukaryotic cell lines
☒	☐	Palaeontology and archaeology
☐	☒	Animals and other organisms
☒	☐	Human research participants
☒	☐	Clinical data
☒	☐	Dual use research of concern

Methods

n/a	Involved in the study	
☐	☒	ChIP-seq
☐	☒	Flow cytometry
☐	☒	MRI-based neuroimaging

Antibodies

Antibodies used	For standard western blot the primary antibodies used: rabbit anti-PARP (Cell Signaling Technology (CST) 9532; 1:500); rabbit anti-cCP9 (CST 7237; 1:500); rabbit anti-cCP3 (CST 9665; 1:500); rabbit anti-USP9x (CST 5751; 1:500); rabbit anti-Bcl-xL (CST 2764; 1:500); rabbit anti-Bcl-2 (CST 4223; 1:500); rabbit anti-Mcl-1 (CST 5453; 1:500); rabbit anti-CPT1A (CST 12252; 1:500); mouse anti-Noxa (Calbiochem OP180, clone 114C307; 1:500); rabbit anti-BIM (CST 2933; 1:500); mouse anti-β-actin (Sigma Aldrich A1978, clone AC15; 1:8,000); rabbit anti-Aurora A/AIK (1G4) (CST 4718; 1:500); rabbit anti-p-Aurora A (Thr288) (CST 3079; 1:500); rabbit anti-Myc (CST 13987; 1:500); and rabbit anti-GSK-3β (27C10) (CST 9315; 1:500). The secondary antibodies were used: anti-rabbit IgG (H+L) secondary antibody, HRP (Thermo Fisher 31460; 1: 3000) and anti-mouse IgG (H+L) secondary antibody, HRP(Thermo Fisher 31460; 1: 3000). For protein capillary electrophoresis the primary antibodies used rabbit anti-HK2 (CST 2106, 1:25); rabbit anti-Glut1 (CST 12939; 1:25); rabbit anti-LDHA (CST 3582, 1:400); rabbit anti-PGC1α (Novus Biologicals NBP1-04676, 1:25), rabbit anti-c-Myc (CST 13987, 1:25); mouse anti-Bcl-2 (R&D System MAB827, 1:25); rabbit anti-ATF4 (CST 11815; 1:25); rabbit anti-Vinculin (Abcam ab129002, 1:500); rabbit anti-Aurora A/AIK (1G4) (CST 4718; 1:500); rabbit anti-GSK-3β (27C10) (CST 9315; 1:25), and rabbit anti-phospho-GSK-3β (Ser9) (CST 5558S; 1:25). The secondary antibodies were used with the manufacturer's instructions: Anti-Rabbit Secondary HRP Antibody (ProteinSimple 042-206) and Anti-Mouse Secondary HRP Antibody (ProteinSimple 042-205).
Validation	All antibodies were described in the literature before and validated. The vendors providing each antibody performed the validation.

Eukaryotic cell lines

Policy information about cell lines

Cell line source(s)	GBM22, GBM12, GBM43, U87, HCT116, and SF188 cells
Authentication	GBM22, GBM12, and GBM43 cells were obtained from Dr. Jann Sarkaria (Mayo Clinic, Rochester, MN) between 2014 and 2020. U87 and HCT116 cell lines were obtained from the American Type Culture Collection (Manassas, VA). SF188 cells were obtained from UCSF (CA) Astrocyte cells were obtained from ScienceCell Research Laboratories (Carlsbad, CA). This was done by STR profiling.
Mycoplasma contamination	Mycoplasma contamination was excluded by the original source. Only low passages and short term cultures of the cells were used before discarding them and using another fresh vial.
Commonly misidentified lines (See ICLAC register)	No Commonly misidentified lines were used in this study.

Animals and other organisms

Policy information about studies involving animals; ARRIVE guidelines recommended for reporting animal research

Laboratory animals	All animals were housed at room temperature with a 12-light/12-dark cycle in the Association for the Assessment and Accreditation of Laboratory Animal Care-accredited animal facility of the University of Pennsylvania. For tumor induction experiments, both female and male eight-week-old mice were used. For the in vivo experiments, 6- to 8-week-old female CrTac:NCr-Foxn1nu were purchased from Taconic Biosciences. The mice were kept under controlled temperature in a 12-h (light)/12-h (dark) cycle, provided with water and food ad libitum. The animals were maintained in small groups (3–5 animals per cage).
Wild animals	No wild animals were used in the study.
Field-collected samples	No field collected samples were used in the study.
Ethics oversight	All procedures were done in accordance with Animal Welfare Regulations and approved by the Institutional Animal Care and Use Committee at the Columbia University Medical Center (AC-AABC6505 and AC-AAAV7451).

Note that full information on the approval of the study protocol must also be provided in the manuscript.

ChIP-seq

Data deposition

☒ Confirm that both raw and final processed data have been deposited in a public database such as GEO.

☒ Confirm that you have deposited or provided access to graph files (e.g. BED files) for the called peaks.

Data access links *May remain private before publication.*	The raw and processed ATAC-sequencing and CHIP-sequencingdata generated in this study have been deposited in the Gene Expression Omnibus (GEO), https://www.ncbi.nlm.nih.gov/geo/, under accession code GSE161572 (https://www.ncbi.nlm.nih.gov/geo/query/acc.cgi?acc=GSE161572) and GSE161573 (https://www.ncbi.nlm.nih.gov/geo/query/acc.cgi?acc=GSE161573) respectively.
Files in database submission	CHIP-seq: GBM22_R1,GBM22AR_R1 (raw and processed bigwig); ATAC-seq: GBM22-1, GBM22-2, GBM22AR-1, GBM22AR-2
Genome browser session (e.g. UCSC)	UCSC

Methodology

Replicates	ATAC-seq with two replicates for each condition and ChIP-seq with one replicate for each condition.
Sequencing depth	single read 50 bp for CHIP-seq and paired end reading for ATAC-seq
Antibodies	H3K27ac antibody (CST 4535, 10 µL/sample), Myc antibody (CST 13987, 10 µL/sample), or Rabbit IgG antibody (CST 2729, 1 µL/sample).
Peak calling parameters	For peak calling, raw sequencing reads were aligned to the human genome HG38 using BWA MEM. PCR duplicates were removed using Picard MarkDuplicates (Picard Tools 1.90). Peaks were called against inputs using Macs2. Normalized bedgraph tracks were generated using the SPMR flag and converted to bigWig using UCSC tool bedGraphToBigWig.
Data quality	Peaks with a score >5 were retained
Software	CHIP-seq and ATAC-seq analyses was performed by using useGalaxy flatform, utilizing bowtie2 (Version 2.4.2), macs2 (Version 2.1.1.20160309) and ucsc-wigtobigwig (Version 357), Integrated Genome Browser 9.1.8 and basepair software.

Flow Cytometry

Plots

Confirm that:

- [x] The axis labels state the marker and fluorochrome used (e.g. CD4-FITC).
- [x] The axis scales are clearly visible. Include numbers along axes only for bottom left plot of group (a 'group' is an analysis of identical markers).
- [x] All plots are contour plots with outliers or pseudocolor plots.
- [x] A numerical value for number of cells or percentage (with statistics) is provided.

Methodology

Sample preparation	Cells were seeded in a 12 well plate with density 3x104 cells per well and allowed to attach overnight. To detect the apoptosis and necrosis, cells were incubated with FITC Annexin V/ propidium iodide according to the manufacturer's instructions (BD Biosciences, 556420). To detect DNA, PI staining was performed according to the manufacturer's instructions (Cell Signaling Technology (CST), 4087S). To detect intrinsic apoptosis staining and loss of mitochondrial membrane potential, TMRE (tetramethylrhodamine ethyl ester perchlorate) staining was performed according to the manufacturer's instructions (CST, 13296S).
Instrument	BD-LSRII
Software	FlowJo software version 10.7
Cell population abundance	10,000 cells were read for each sample.
Gating strategy	The cells were gated through forward scatter (FSC) and side scatter (SSC) and then analyzed for Annexin V and PI fluorescence. Alternatively, cells were analyzed for TMRE fluorescence or for PI staining.

- [x] Tick this box to confirm that a figure exemplifying the gating strategy is provided in the Supplementary Information.

Magnetic resonance imaging

Experimental design

Design type	MRI was performed in the resting state with the animals being anesthetized.
Design specifications	Not applicable
Behavioral performance measures	Not applicable

Acquisition

Imaging type(s)	structural
Field strength	All MRI experiments were performed on a 9.4Tesla MRI system operating on Paravison (PV 6.0.1) software platform (Bruker, Billerica, USA)
Sequence & imaging parameters	A 2D multislice T2w fast spin echo " Rapid Acquisition with Refocused Echoes" (RARE) sequence was used with the following parameters: TR=3200 ms, effective TE=60ms, in-plane resolution=74 µm, slice thickness=0.7mm, number of average = 6, total scan time ~8 minutes.
Area of acquisition	whole brain scan was used.

Diffusion MRI ☐ Used ☒ Not used

Preprocessing

Preprocessing software	*Provide detail on software version and revision number and on specific parameters (model/functions, brain extraction, segmentation, smoothing kernel size, etc.).*
Normalization	*If data were normalized/standardized, describe the approach(es): specify linear or non-linear and define image types used for transformation OR indicate that data were not normalized and explain rationale for lack of normalization.*
Normalization template	*Describe the template used for normalization/transformation, specifying subject space or group standardized space (e.g. original Talairach, MNI305, ICBM152) OR indicate that the data were not normalized.*
Noise and artifact removal	*Describe your procedure(s) for artifact and structured noise removal, specifying motion parameters, tissue signals and physiological signals (heart rate, respiration).*

Volume censoring | 3D Slicer software (www.slicer.org) was used for tumor volume measurement. Semi-automatic thresholding method was used to segment the tumor and a polygon function was used to manually define the tumor border. Tumor volumes were calculated before and after the treatment.

Statistical modeling & inference

Model type and settings

Specify type (mass univariate, multivariate, RSA, predictive, etc.) and describe essential details of the model at the first and second levels (e.g. fixed, random or mixed effects; drift or auto-correlation).

Effect(s) tested

Define precise effect in terms of the task or stimulus conditions instead of psychological concepts and indicate whether ANOVA or factorial designs were used.

Specify type of analysis: ☒ Whole brain ☐ ROI-based ☐ Both

Statistic type for inference
(See Eklund et al. 2016)

Specify voxel-wise or cluster-wise and report all relevant parameters for cluster-wise methods.

Correction

Describe the type of correction and how it is obtained for multiple comparisons (e.g. FWE, FDR, permutation or Monte Carlo).

Models & analysis

n/a	Involved in the study
☒	☐ Functional and/or effective connectivity
☒	☐ Graph analysis
☒	☐ Multivariate modeling or predictive analysis
