# Supplementary material for: Aurora kinase A inhibition reverses the Warburg effect and elicits unique metabolic vulnerabilities in glioblastoma
Source: Nat Commun. 2021 Sep 1;12:5203. doi: 10.1038/s41467-021-25501-x (PMC8410792; doi:10.1038/s41467-021-25501-x)
Supplement: Supplementary file 4 — Source Data [file 41467_2021_25501_MOESM4_ESM.zip › Source Data/Uncropped Western blot Images.pdf]

Figure 1b

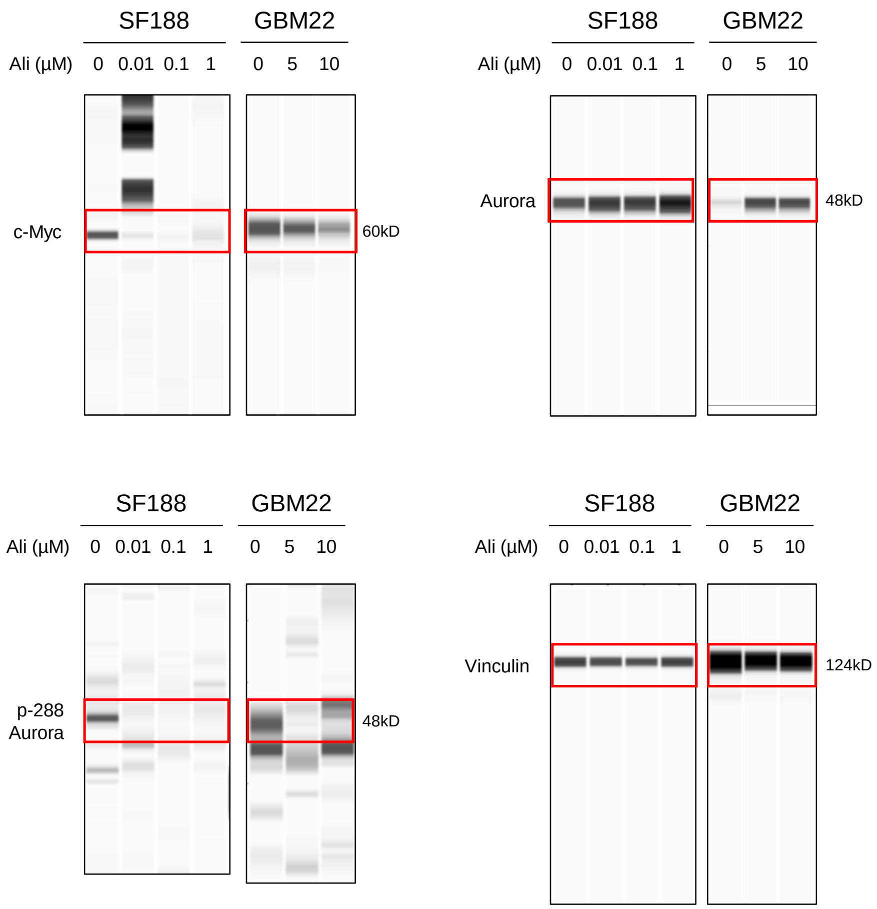

SF188
GBM22
Ali (µM)
0 0.01 0.1 1
0 5 10
c-Myc
60kD
SF188
GBM22
Ali (µM)
0 0.01 0.1 1
0 5 10
Aurora
48kD
SF188
GBM22
Ali (µM)
0 0.01 0.1 1
0 5 10
p-288 Aurora
48kD
SF188
GBM22
Ali (µM)
0 0.01 0.1 1
0 5 10
Vinculin
124kD

Figure 1c

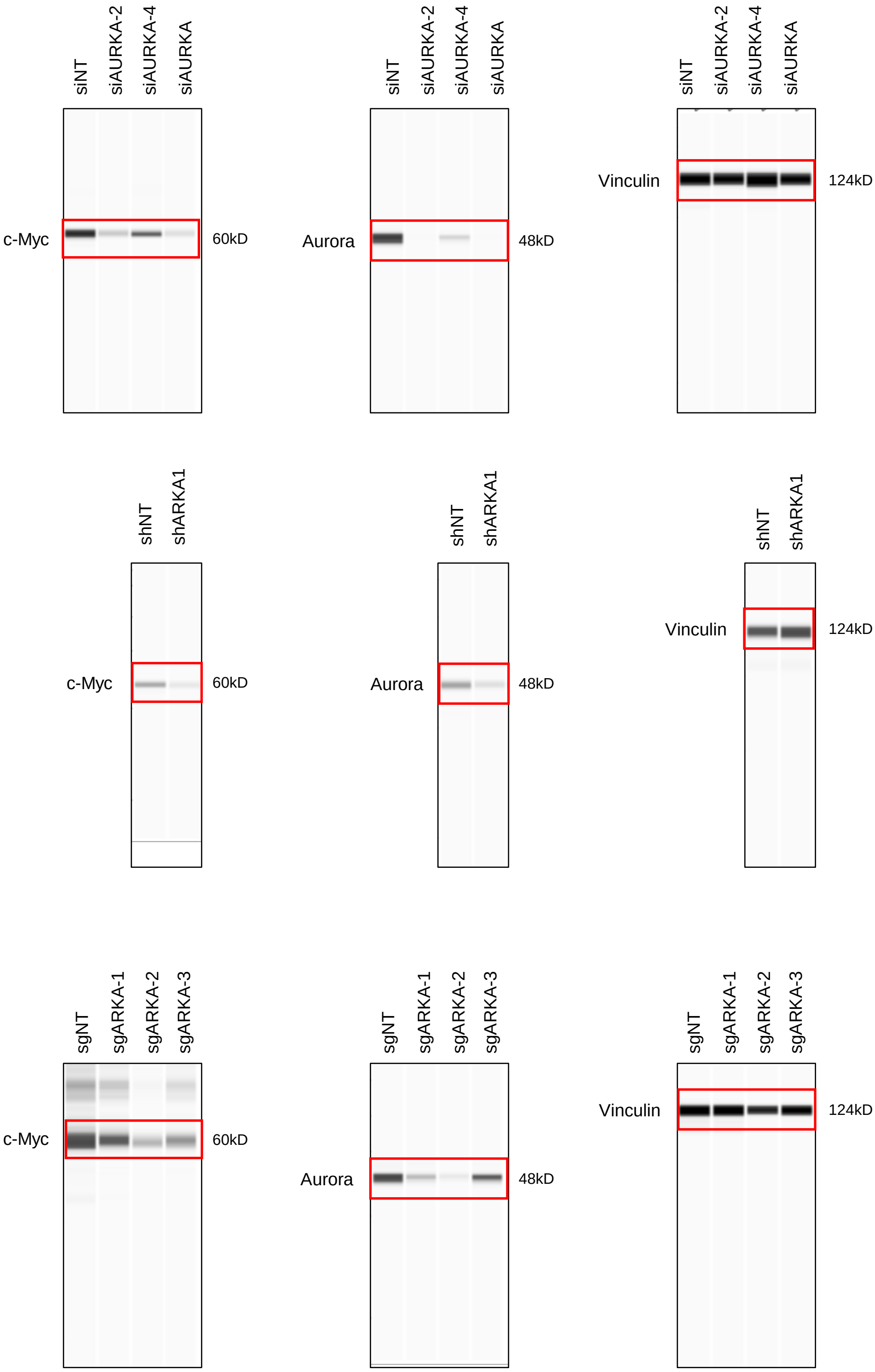

siNT
siAURKA-2
siAURKA-4
siAURKA
c-Myc
60kD
Aurora
48kD
Vinculin
124kD
shNT
shARKA1
c-Myc
60kD
Aurora
48kD
Vinculin
124kD
sgNT
sgARKA-1
sgARKA-2
sgARKA-3
c-Myc
60kD
Aurora
48kD
Vinculin
124kD

Figure 1f

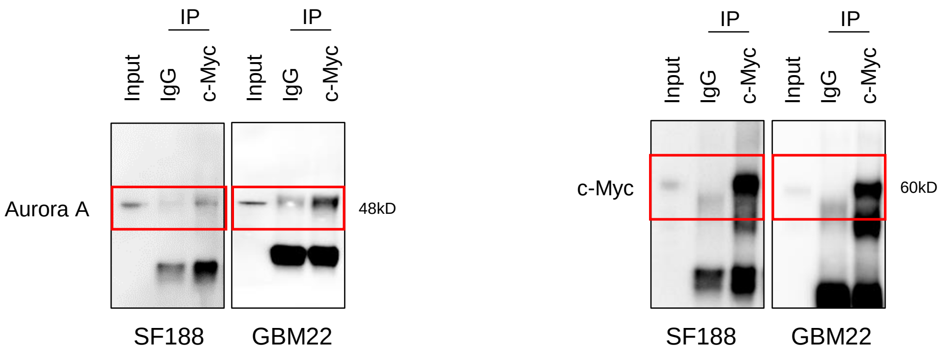

Figure 1g

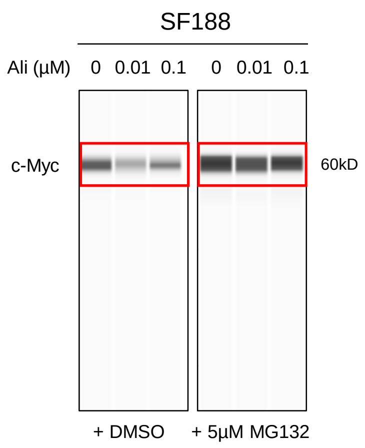

SF188
Ali (µM) 0 0.01 0.1 0 0.01 0.1
c-Myc
60kD
+ DMSO
+ 5µM MG132

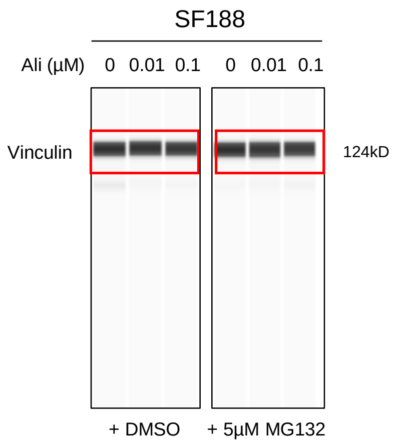

SF188
Ali (µM) 0 0.01 0.1 0 0.01 0.1
Vinculin
124kD
+ DMSO
+ 5µM MG132

Figure 1h

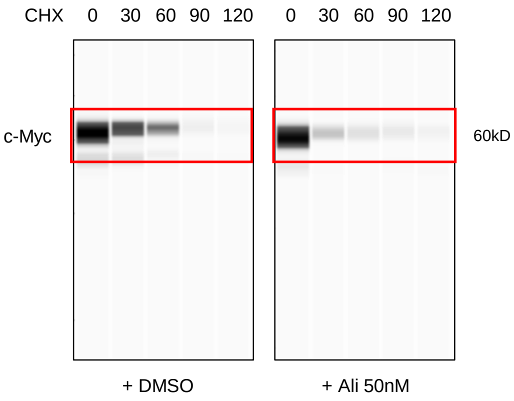
CHX 0 30 60 90 120 0 30 60 90 120
c-Myc
60kD
+ DMSO
+ Ali 50nM

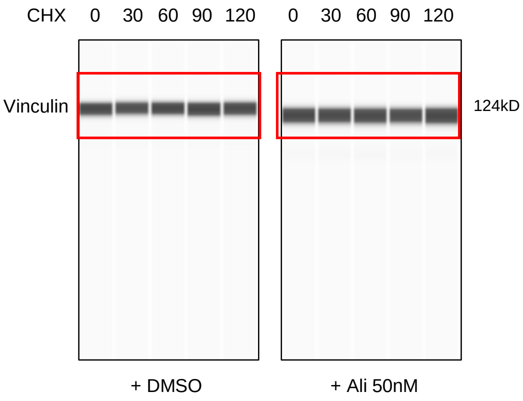
CHX 0 30 60 90 120 0 30 60 90 120
Vinculin
124kD
+ DMSO
+ Ali 50nM

Figure 1j

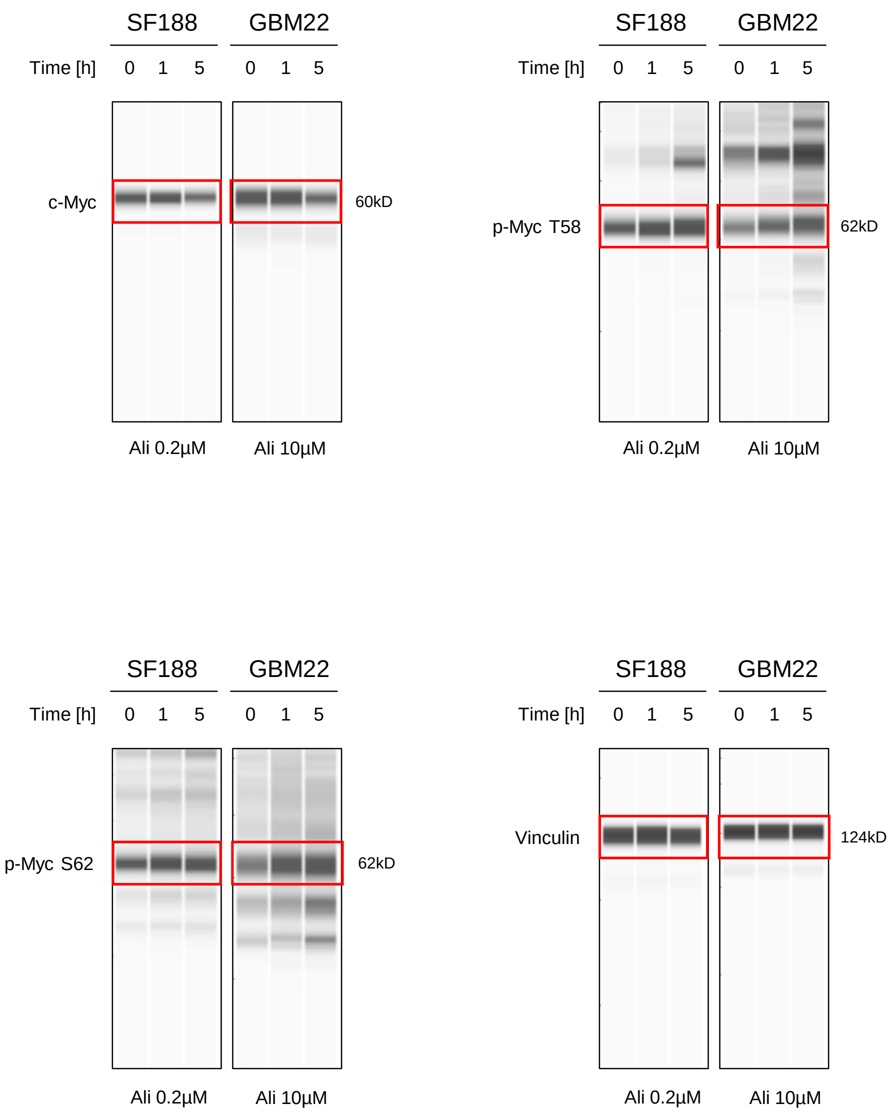
SF188
GBM22
Time [h]
0 1 5
0 1 5
c-Myc
60kD
Ali 0.2µM
Ali 10µM
SF188
GBM22
Time [h]
0 1 5
0 1 5
p-Myc T58
62kD
Ali 0.2µM
Ali 10µM
SF188
GBM22
Time [h]
0 1 5
0 1 5
p-Myc S62
62kD
Ali 0.2µM
Ali 10µM
SF188
GBM22
Time [h]
0 1 5
0 1 5
Vinculin
124kD
Ali 0.2µM
Ali 10µM

Figure 1l

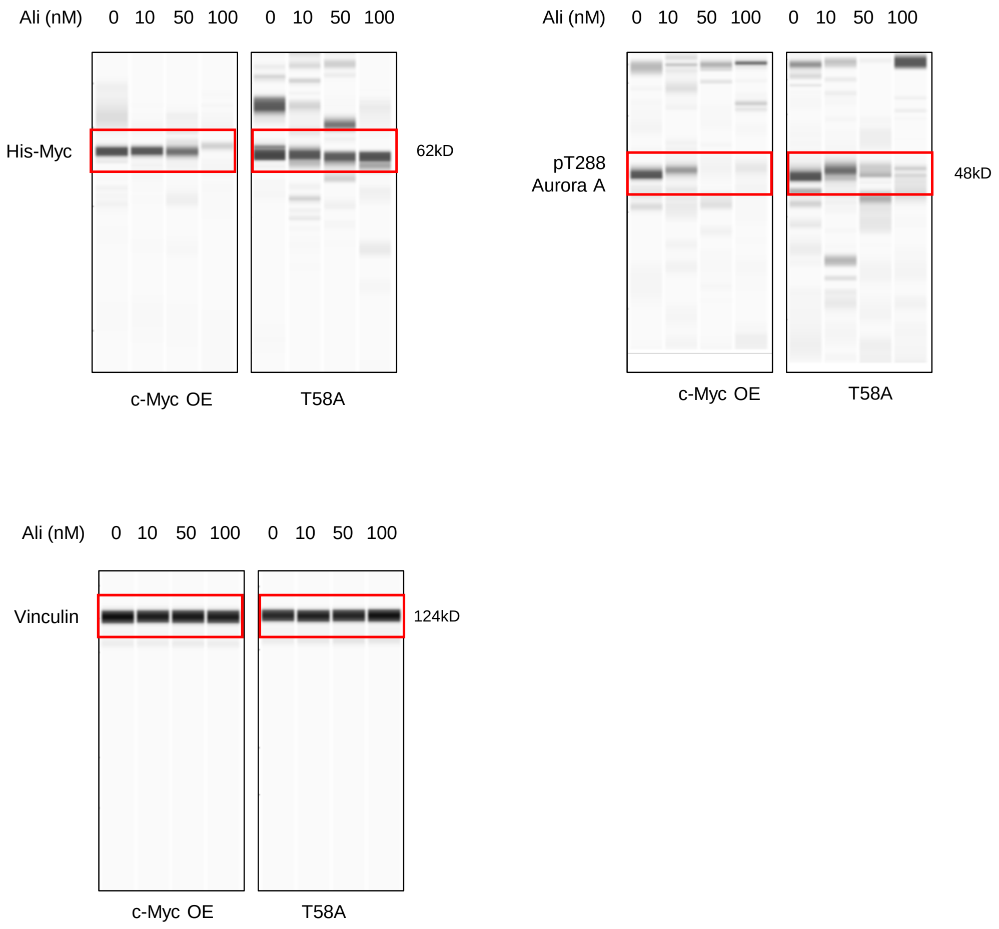
Ali (nM)
0 10 50 100 0 10 50 100
His-Myc
62kD
c-Myc OE
T58A
Ali (nM)
0 10 50 100 0 10 50 100
pT288
Aurora A
48kD
c-Myc OE
T58A
Ali (nM)
0 10 50 100 0 10 50 100
Vinculin
124kD
c-Myc OE
T58A

Figure 1n

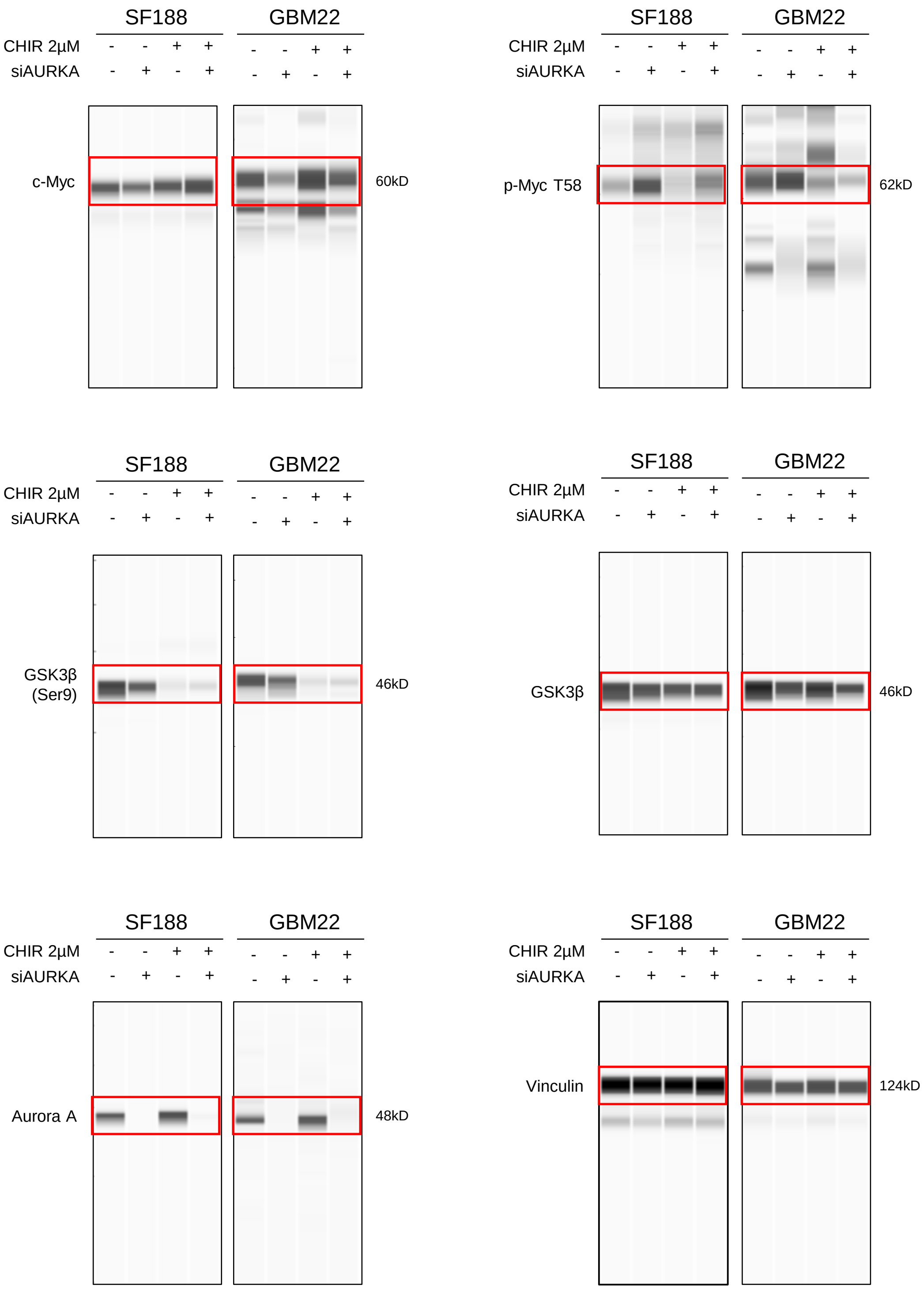
SF188
GBM22
CHIR 2μM
siAURKA
- - + +
- + - +
c-Myc
60kD
p-Myc T58
62kD
GSK3β (Ser9)
46kD
GSK3β
46kD
Aurora A
48kD
Vinculin
124kD

Figure 1o

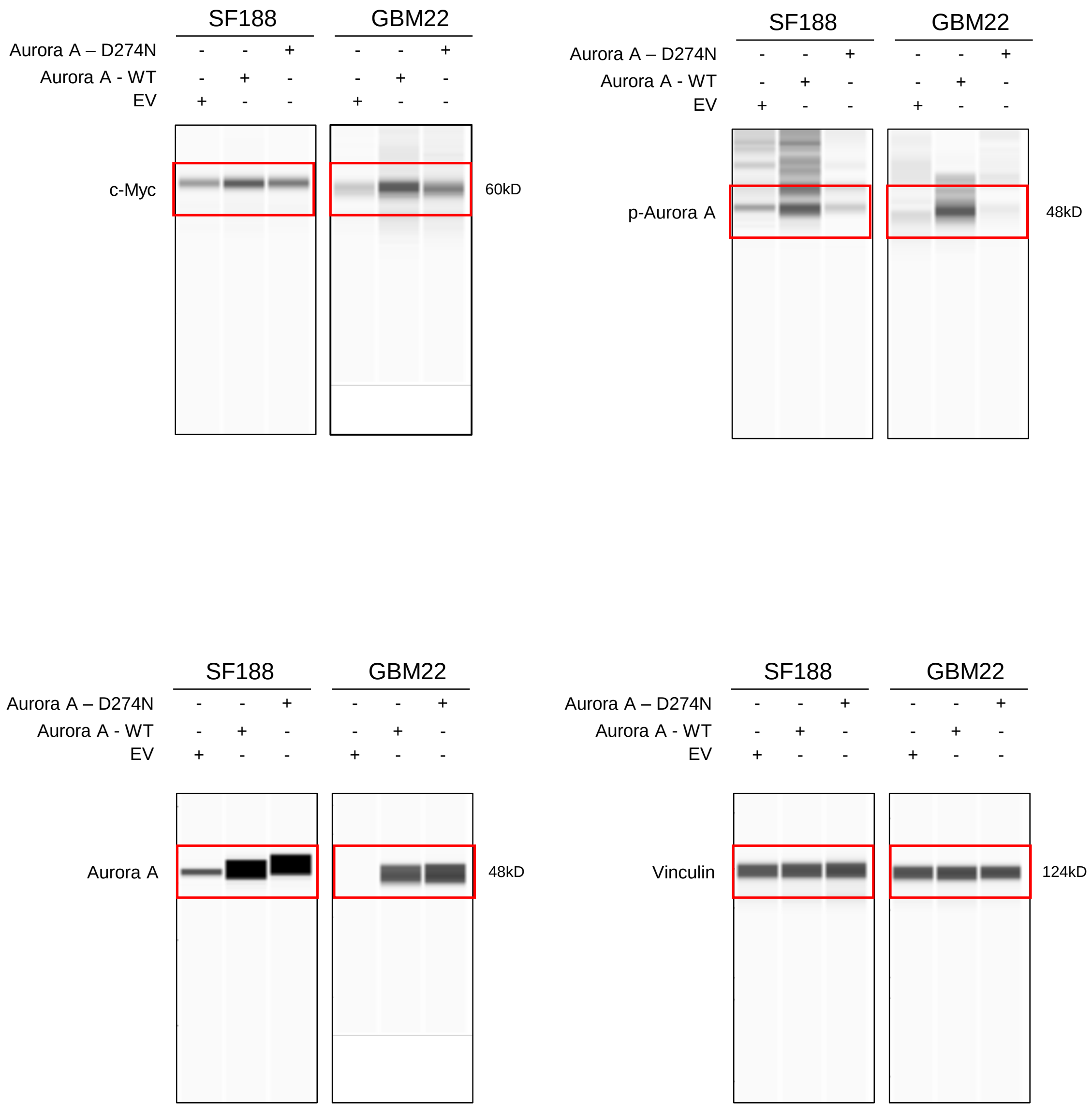
SF188
GBM22
Aurora A – D274N
Aurora A - WT
EV
c-Myc
60kD
p-Aurora A
48kD
Aurora A
48kD
Vinculin
124kD
+
-

Figure 2f

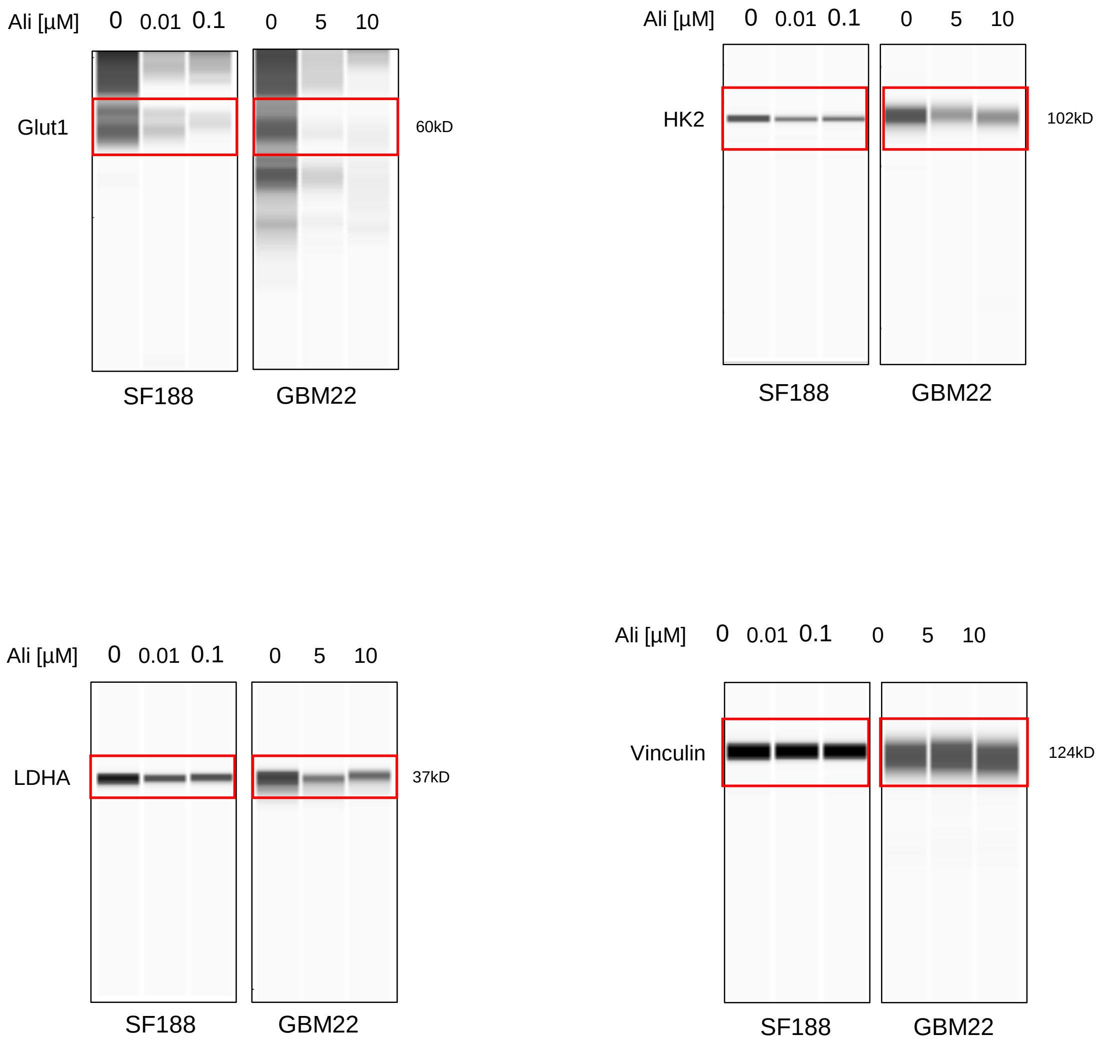
Ali [µM]
0
0.01
0.1
0
5
10
Glut1
60kD
SF188
GBM22
Ali [µM]
0
0.01
0.1
0
5
10
HK2
102kD
SF188
GBM22
Ali [µM]
0
0.01
0.1
0
5
10
LDHA
37kD
SF188
GBM22
Ali [µM]
0
0.01
0.1
0
5
10
Vinculin
124kD
SF188
GBM22

Figure 2g

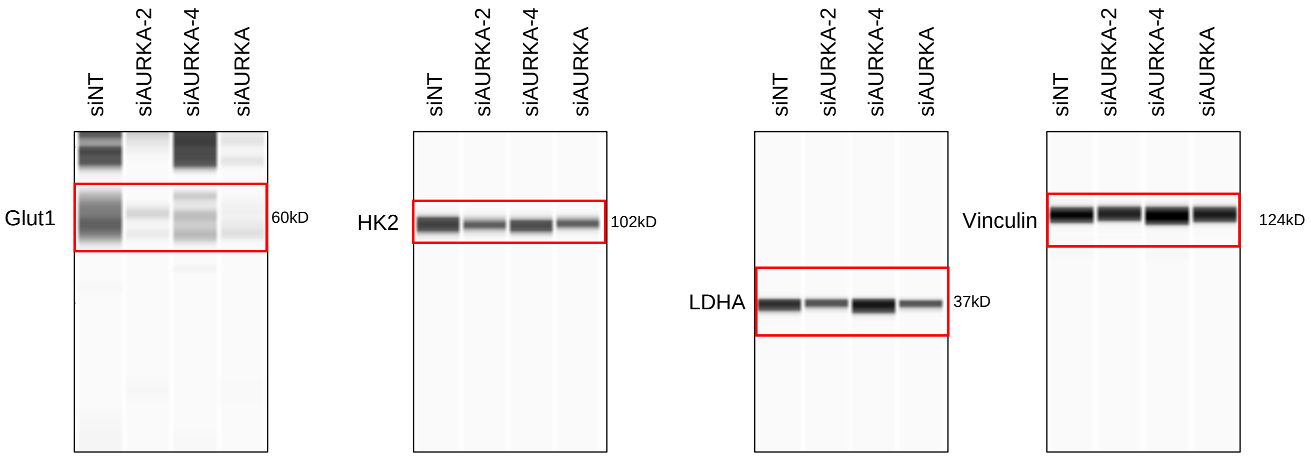
siNT
siAURKA-2
siAURKA-4
siAURKA
Glut1
60kD
HK2
102kD
LDHA
37kD
Vinculin
124kD

Figure 2I

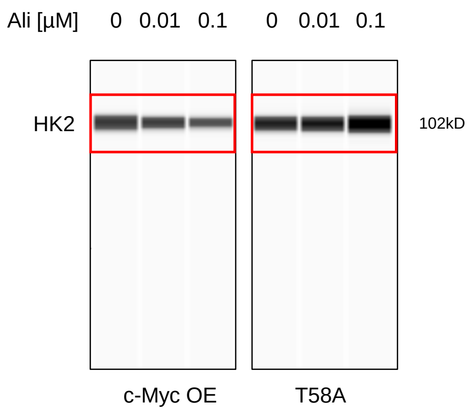
Ali [µM]
0 0.01 0.1 0 0.01 0.1
HK2
102kD
c-Myc OE
T58A

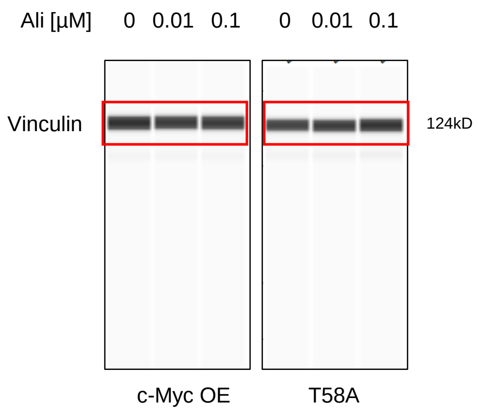
Ali [µM]
0 0.01 0.1 0 0.01 0.1
Vinculin
124kD
c-Myc OE
T58A

Figure 4e

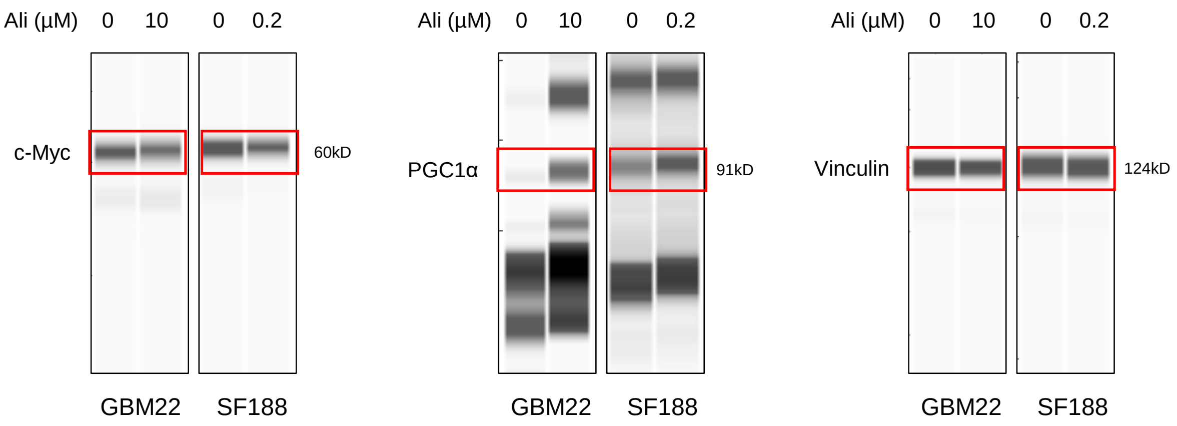
Ali (µM) 0 10 0 0.2
c-Myc
60kD
GBM22 SF188
Ali (µM) 0 10 0 0.2
PGC1α
91kD
GBM22 SF188
Ali (µM) 0 10 0 0.2
Vinculin
124kD
GBM22 SF188

Figure 4f

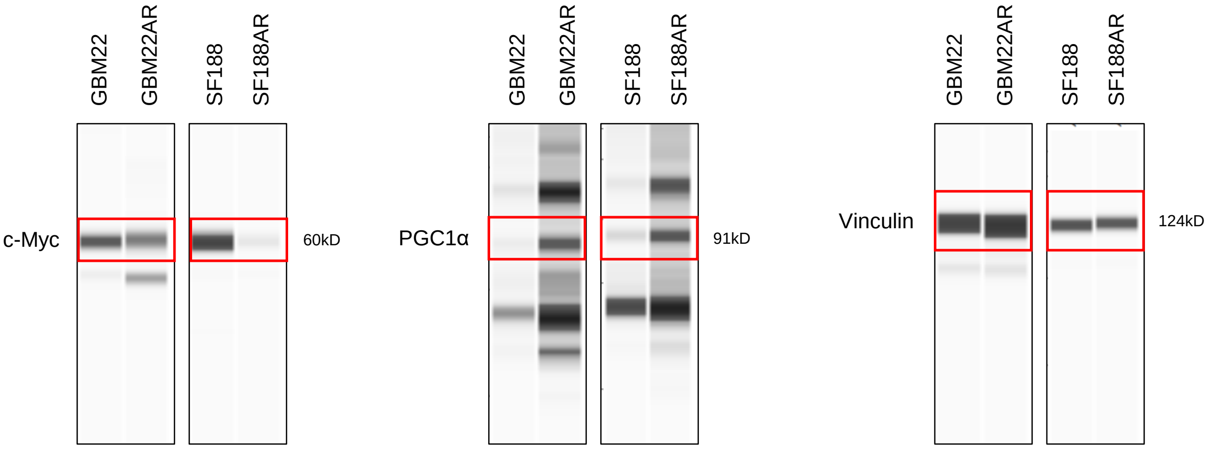
GBM22
GBM22AR
SF188
SF188AR
c-Myc
60kD
PGC1α
91kD
Vinculin
124kD

Figure 4g

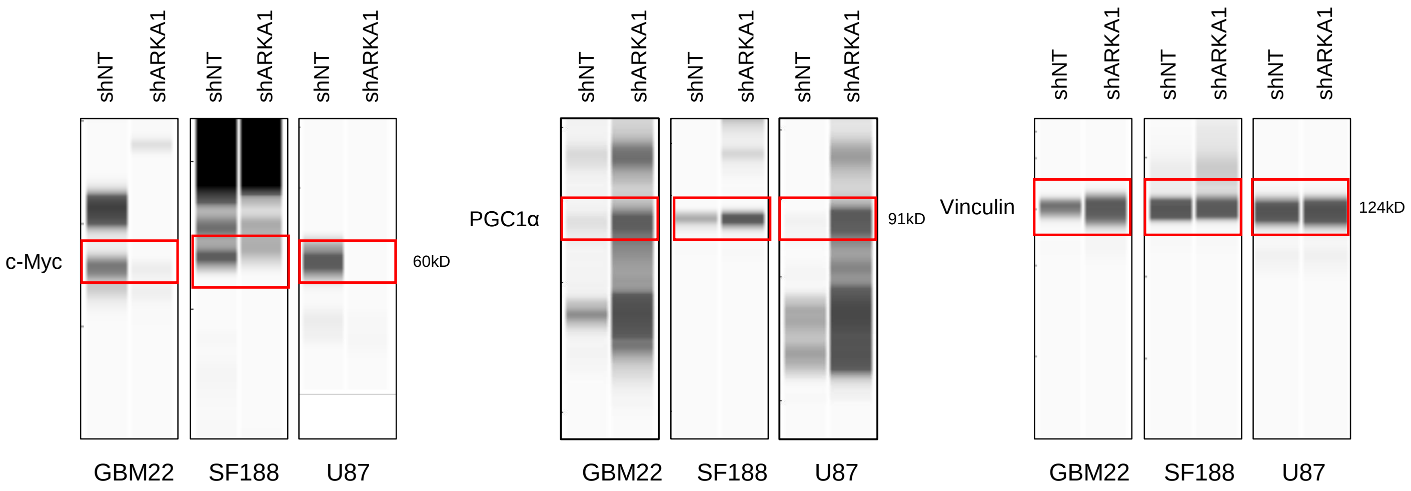
shNT
shARKA1
shNT
shARKA1
shNT
shARKA1
c-Myc
60kD
GBM22
SF188
U87
shNT
shARKA1
shNT
shARKA1
shNT
shARKA1
PGC1α
91kD
GBM22
SF188
U87
shNT
shARKA1
shNT
shARKA1
shNT
shARKA1
Vinculin
124kD
GBM22
SF188
U87

Figure 4h

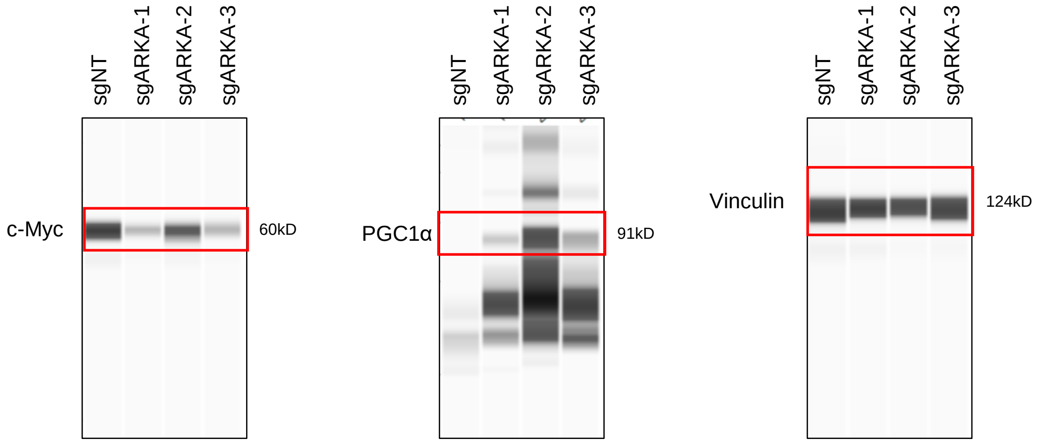
sgNT
sgARKA-1
sgARKA-2
sgARKA-3
c-Myc
60kD
PGC1α
91kD
Vinculin
124kD

Figure 4m

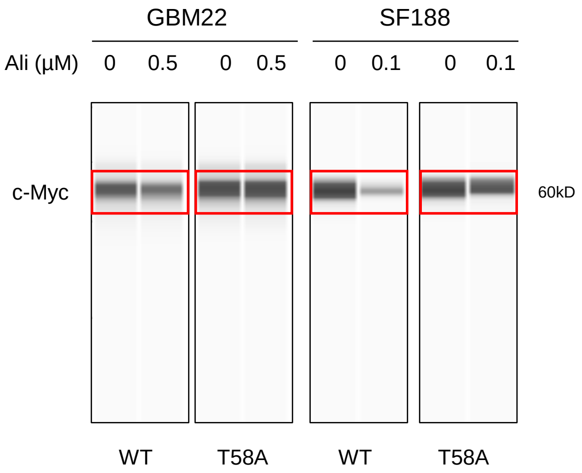
GBM22
SF188
Ali (µM)
0
0.5
0
0.5
0
0.1
0
0.1
c-Myc
60kD
WT
T58A
WT
T58A

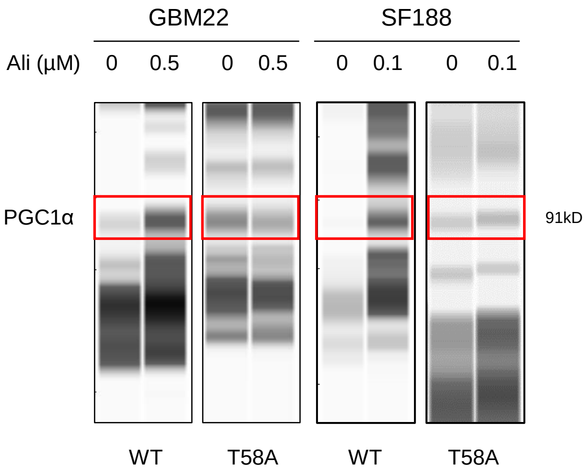
GBM22
SF188
Ali (µM)
0
0.5
0
0.5
0
0.1
0
0.1
PGC1α
91kD
WT
T58A
WT
T58A

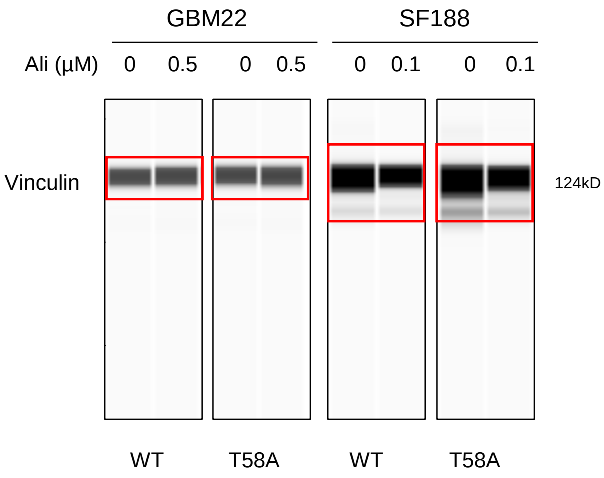
GBM22
SF188
Ali (µM)
0
0.5
0
0.5
0
0.1
0
0.1
Vinculin
124kD
WT
T58A
WT
T58A

Figure 4n

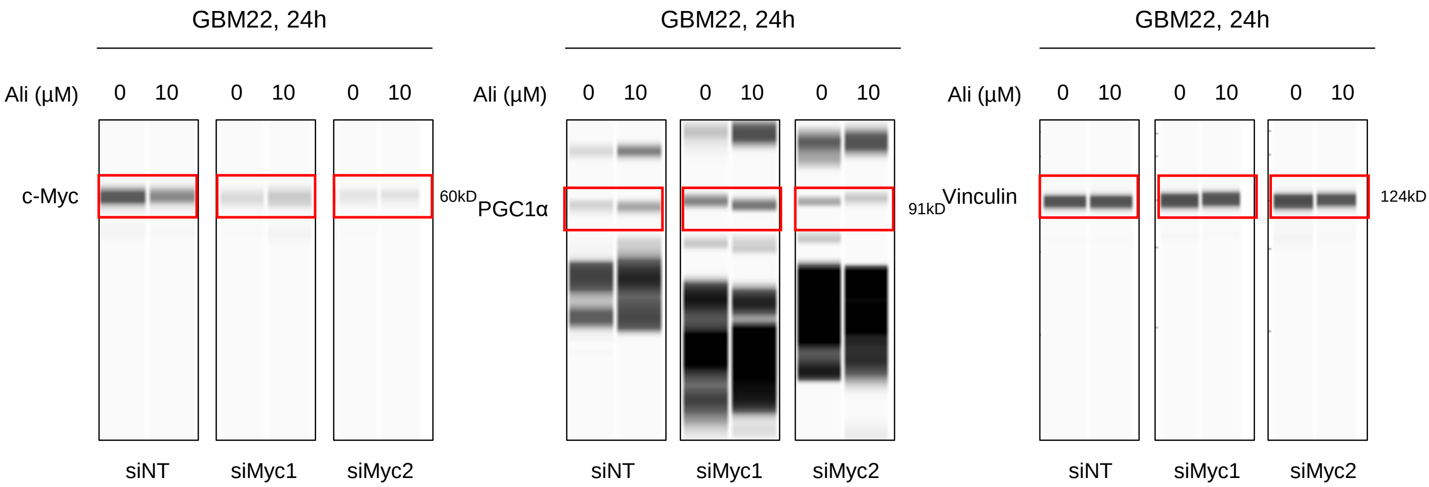

GBM22, 24h
Ali (µM)
0
10
0
10
0
10
c-Myc
60kD
siNT
siMyc1
siMyc2
GBM22, 24h
Ali (µM)
0
10
0
10
0
10
PGC1α
91kD
siNT
siMyc1
siMyc2
GBM22, 24h
Ali (µM)
0
10
0
10
0
10
Vinculin
124kD
siNT
siMyc1
siMyc2

Figure 6I

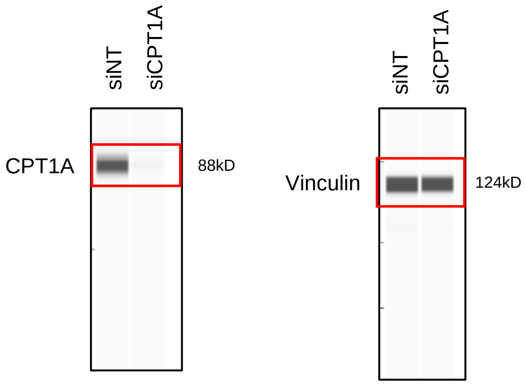

Figure S1a

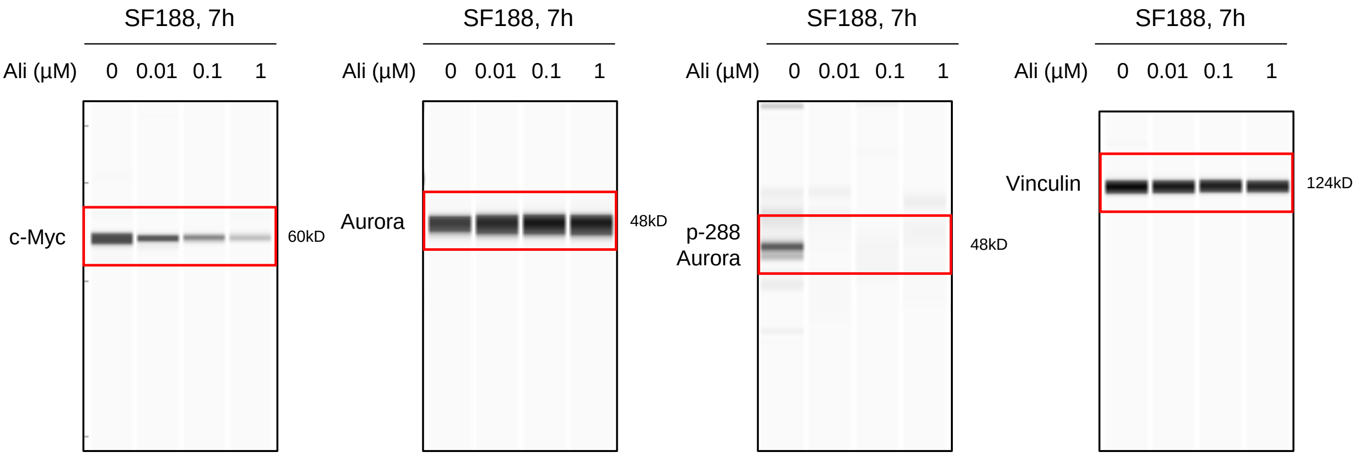
SF188, 7h
Ali (µM) 0 0.01 0.1 1
c-Myc
60kD
SF188, 7h
Ali (µM) 0 0.01 0.1 1
Aurora
48kD
SF188, 7h
Ali (µM) 0 0.01 0.1 1
p-288
Aurora
48kD
SF188, 7h
Ali (µM) 0 0.01 0.1 1
Vinculin
124kD

Figure S1b

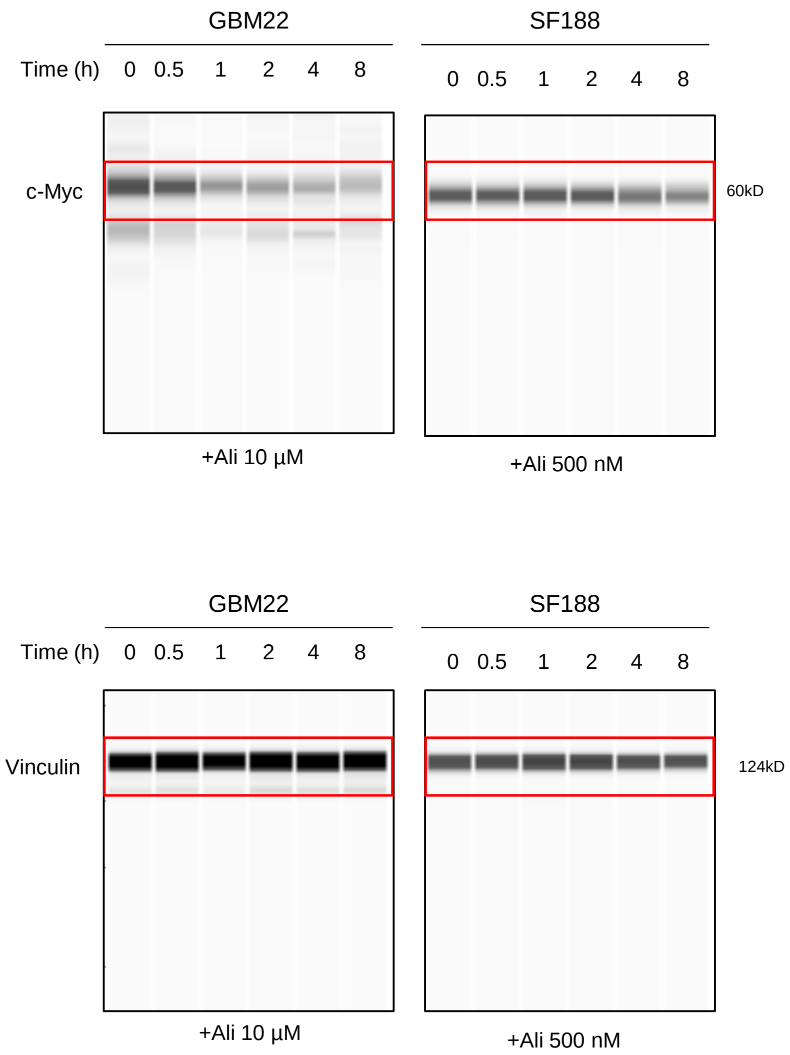
GBM22
SF188
Time (h)
0 0.5 1 2 4 8
0 0.5 1 2 4 8
c-Myc
60kD
+Ali 10 µM
+Ali 500 nM
GBM22
SF188
Time (h)
0 0.5 1 2 4 8
0 0.5 1 2 4 8
Vinculin
124kD
+Ali 10 µM
+Ali 500 nM

Figure S1c

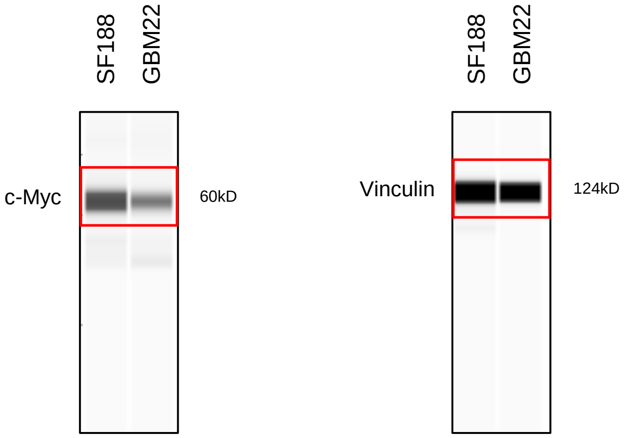

Figure S1h

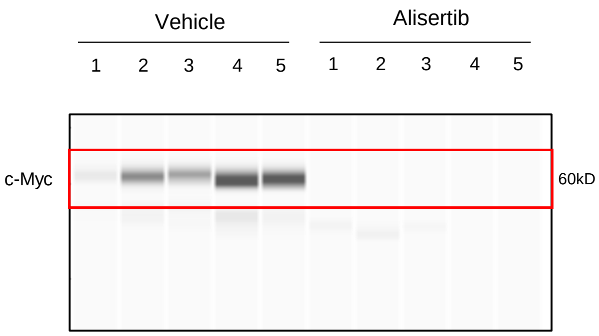
Vehicle
Alisertib
1 2 3 4 5 1 2 3 4 5
c-Myc
60kD

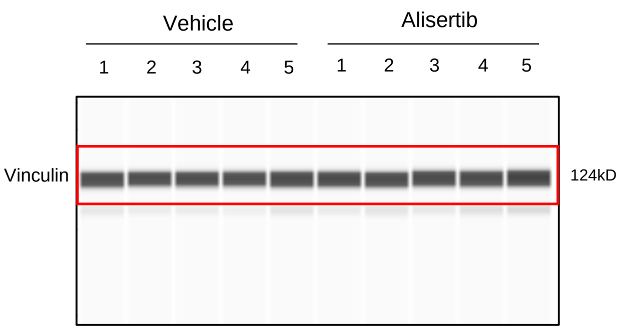
Vehicle
Alisertib
1 2 3 4 5 1 2 3 4 5
Vinculin
124kD

Figure S3a

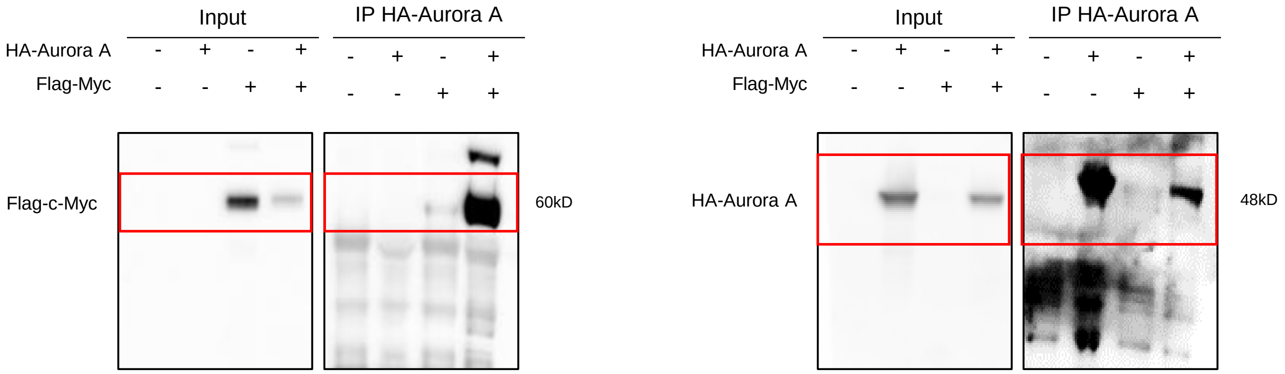
Input
IP HA-Aurora A
HA-Aurora A - + - + - + - +
Flag-Myc - - + + - - + +
Flag-c-Myc
60kD
Input
IP HA-Aurora A
HA-Aurora A - + - + - + - +
Flag-Myc - - + + - - + +
HA-Aurora A
48kD

Figure S3g

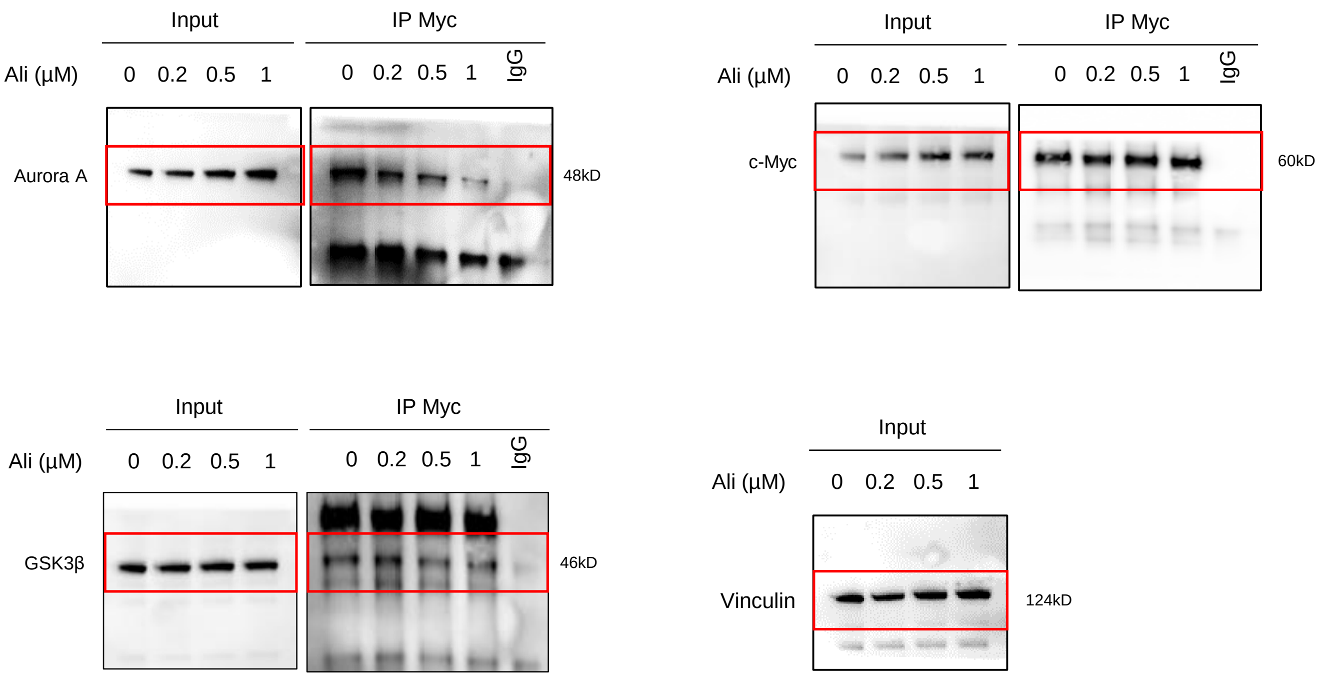
Input
IP Myc
Ali (µM)
0
0.2
0.5
1
IgG
Aurora A
48kD
c-Myc
60kD
GSK3β
46kD
Vinculin
124kD

Figure S3j

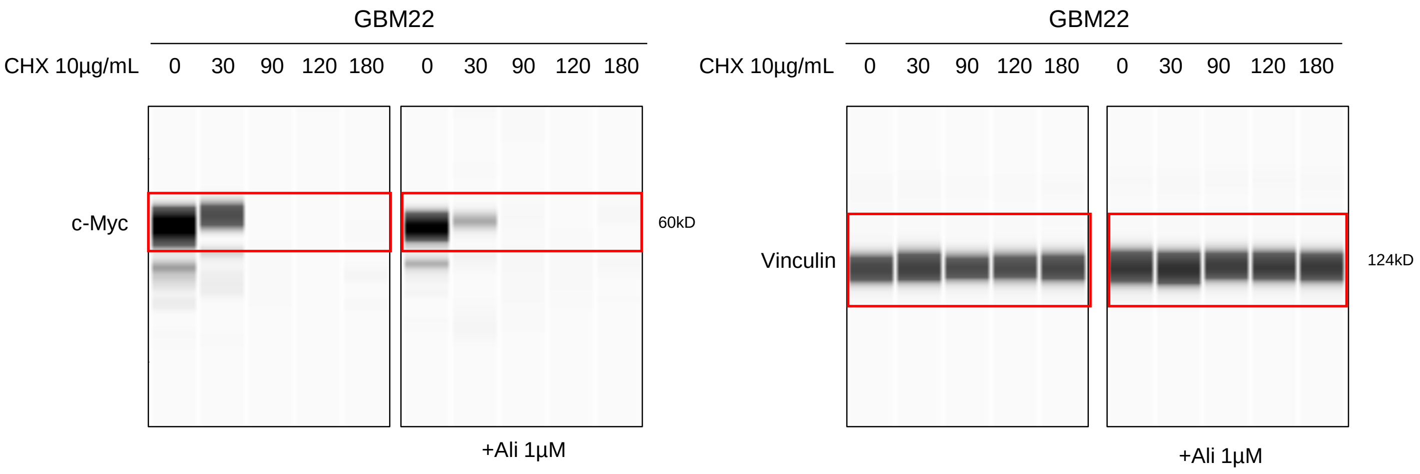
GBM22
CHX 10µg/mL 0 30 90 120 180 0 30 90 120 180
c-Myc
60kD
+Ali 1µM
GBM22
CHX 10µg/mL 0 30 90 120 180 0 30 90 120 180
Vinculin
124kD
+Ali 1µM

Figure S3k

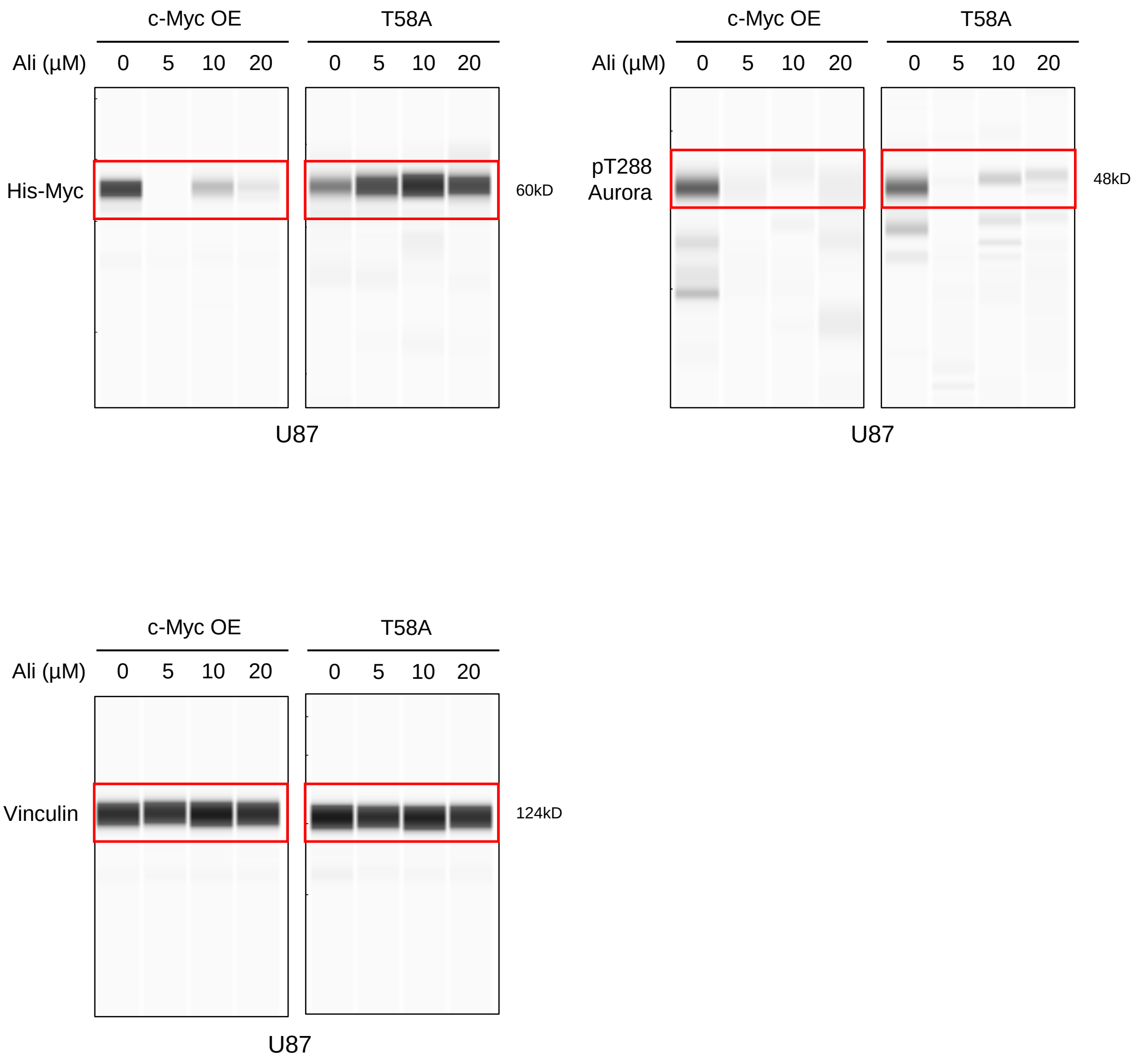
c-Myc OE
T58A
Ali (μM)
0 5 10 20
0 5 10 20
His-Myc
60kD
U87
c-Myc OE
T58A
Ali (μM)
0 5 10 20
0 5 10 20
pT288
Aurora
48kD
U87
c-Myc OE
T58A
Ali (μM)
0 5 10 20
0 5 10 20
Vinculin
124kD
U87

Figure S3I

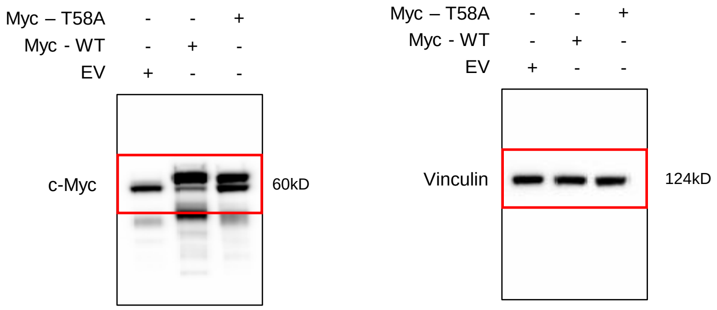

Figure S4a

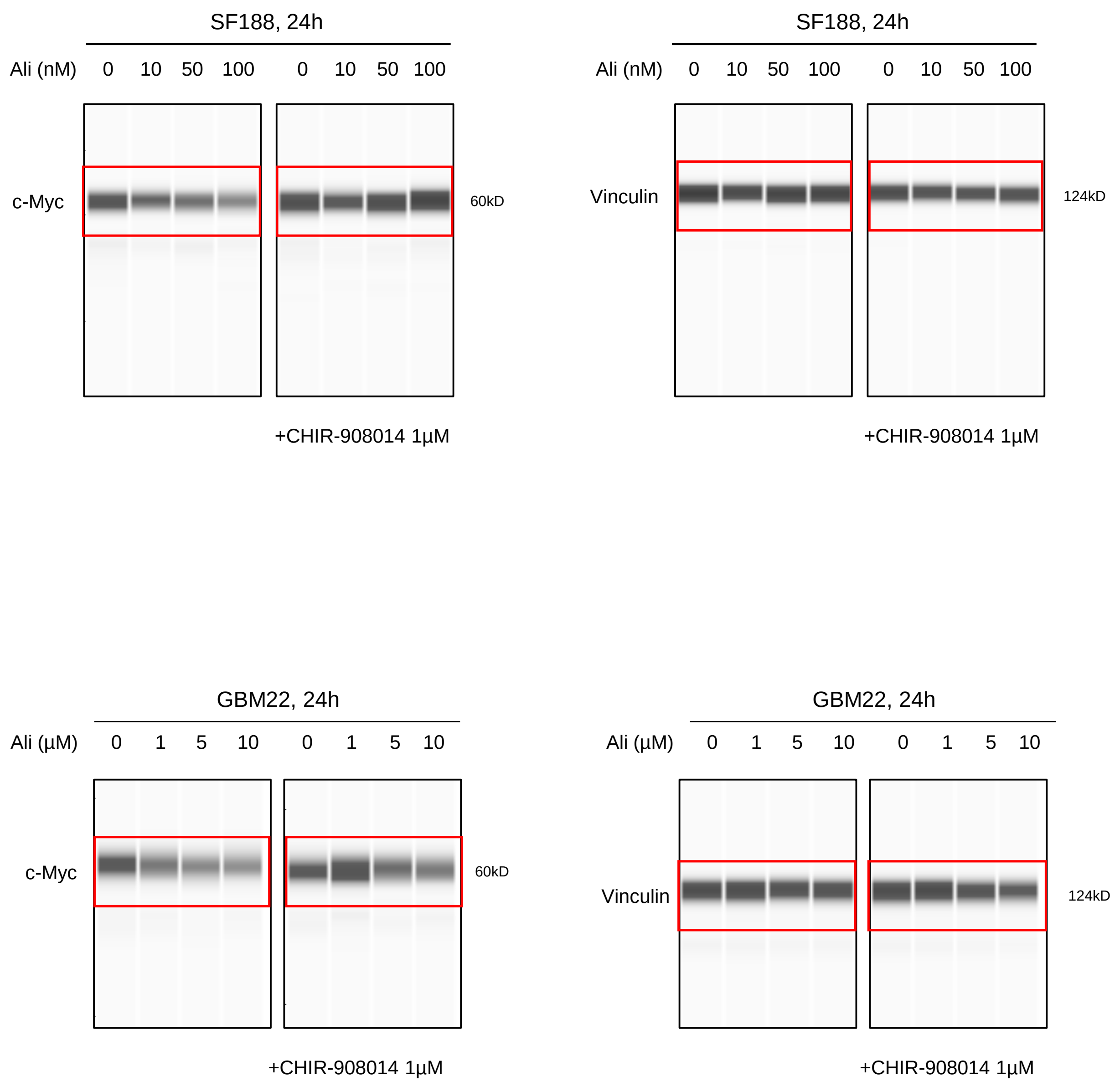
SF188, 24h
Ali (nM) 0 10 50 100 0 10 50 100
c-Myc
60kD
+CHIR-908014 1µM
SF188, 24h
Ali (nM) 0 10 50 100 0 10 50 100
Vinculin
124kD
+CHIR-908014 1µM
GBM22, 24h
Ali (µM) 0 1 5 10 0 1 5 10
c-Myc
60kD
+CHIR-908014 1µM
GBM22, 24h
Ali (µM) 0 1 5 10 0 1 5 10
Vinculin
124kD
+CHIR-908014 1µM

Figure S4b

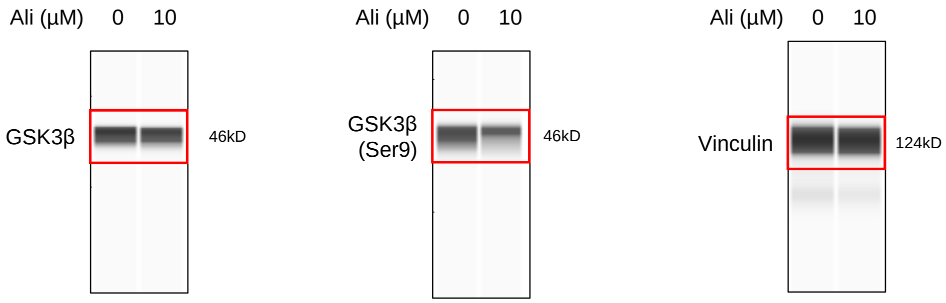
Ali (µM) 0 10
GSK3β
46kD
Ali (µM) 0 10
GSK3β (Ser9)
46kD
Ali (µM) 0 10
Vinculin
124kD

Figure S4c

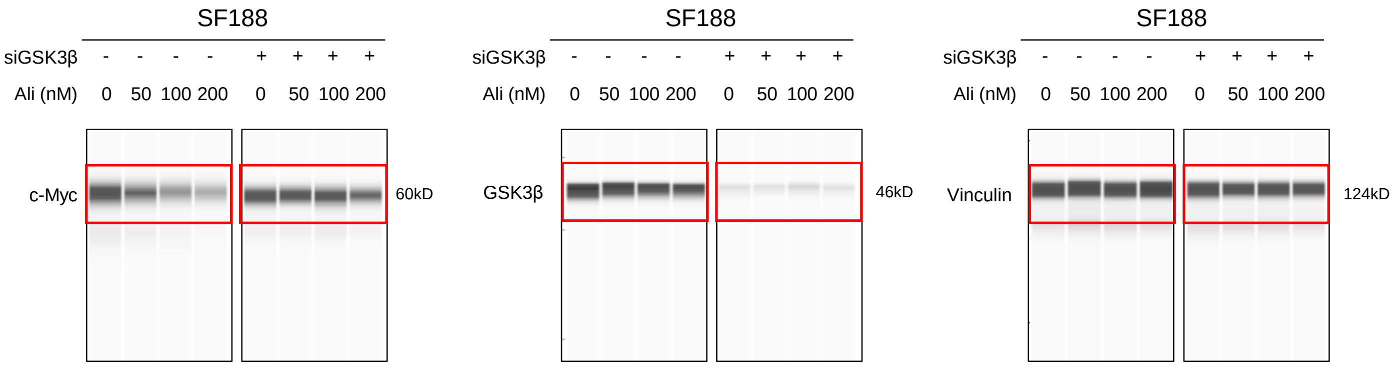

SF188
siGSK3β - - - - + + + +
Ali (nM) 0 50 100 200 0 50 100 200
c-Myc
60kD
SF188
siGSK3β - - - - + + + +
Ali (nM) 0 50 100 200 0 50 100 200
GSK3β
46kD
SF188
siGSK3β - - - - + + + +
Ali (nM) 0 50 100 200 0 50 100 200
Vinculin
124kD

Figure S4e

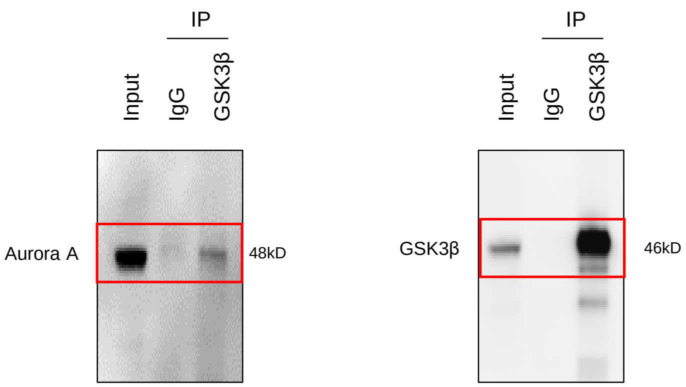
IP
Input
IgG
GSK3β
Aurora A
48kD
IP
Input
IgG
GSK3β
GSK3β
46kD

Figure S5a

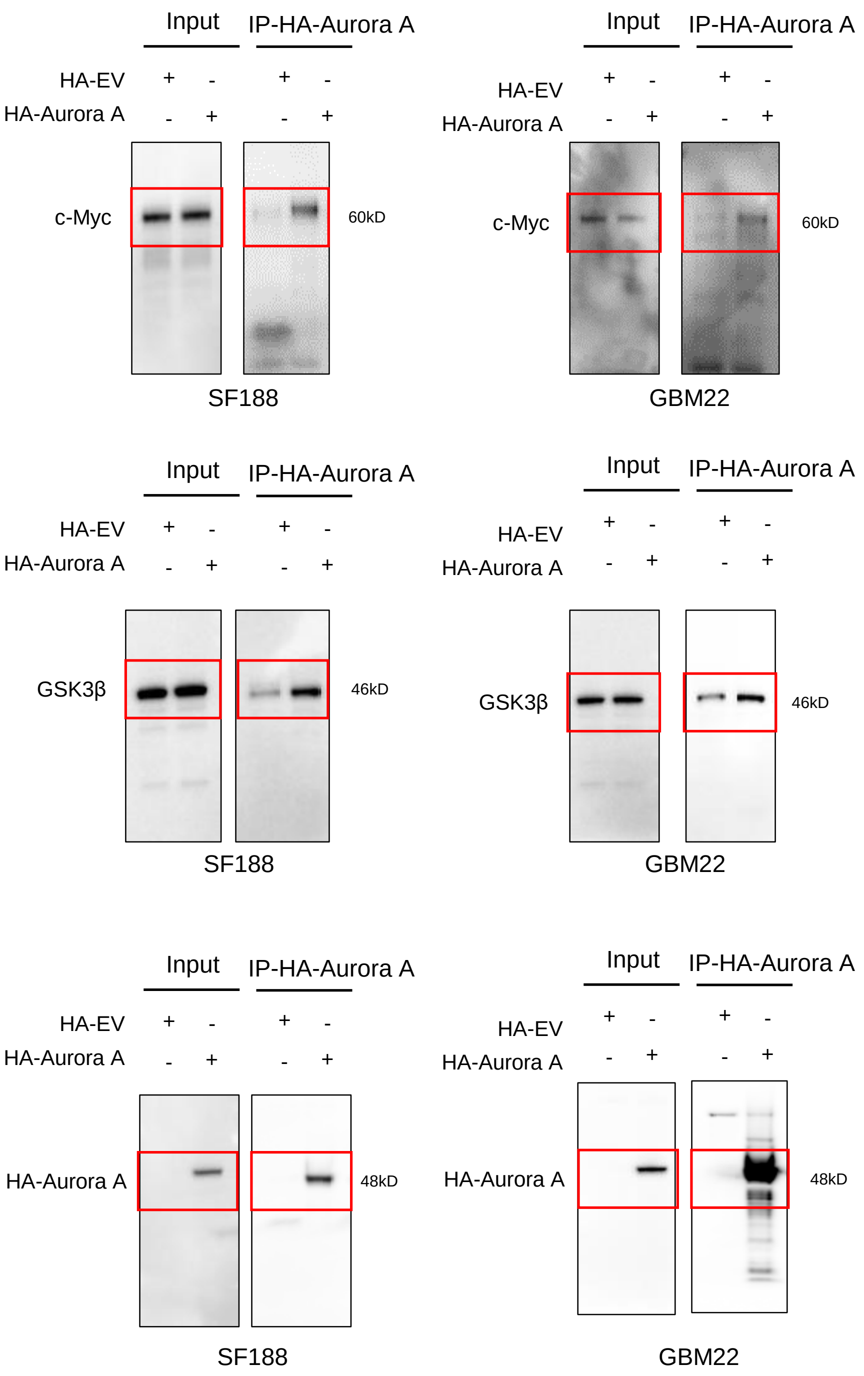

Input
IP-HA-Aurora A
HA-EV
HA-Aurora A
c-Myc
60kD
SF188
GBM22
GSK3β
46kD
48kD

Figure S5b

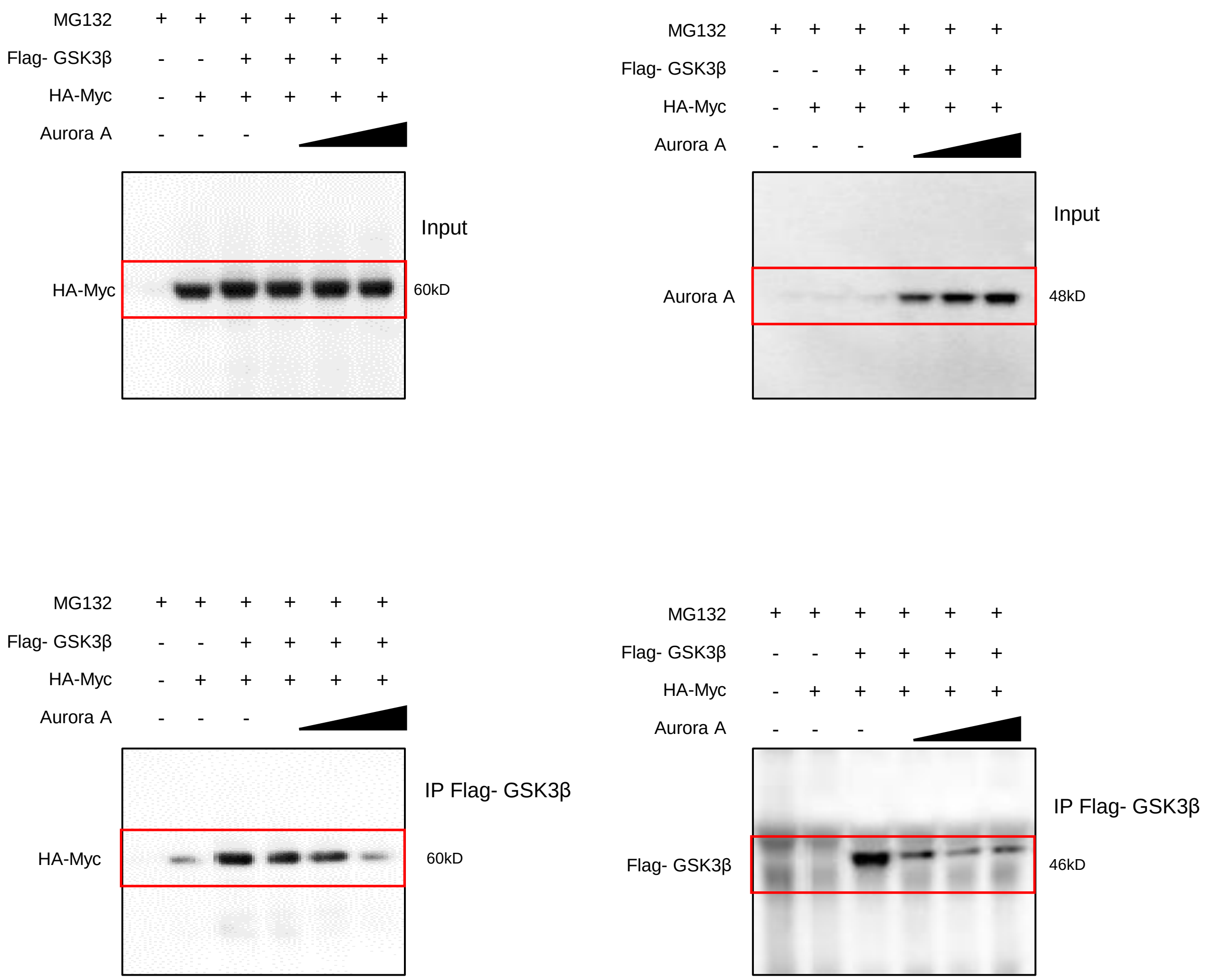
MG132 + + + + + +
Flag- GSK3β - - + + + +
HA-Myc - + + + + +
Aurora A - - -
HA-Myc
Input
60kD
MG132 + + + + + +
Flag- GSK3β - - + + + +
HA-Myc - + + + + +
Aurora A - - -
Aurora A
Input
48kD
MG132 + + + + + +
Flag- GSK3β - - + + + +
HA-Myc - + + + + +
Aurora A - - -
HA-Myc
IP Flag- GSK3β
60kD
MG132 + + + + + +
Flag- GSK3β - - + + + +
HA-Myc - + + + + +
Aurora A - - -
Flag- GSK3β
IP Flag- GSK3β
46kD

Figure S5c

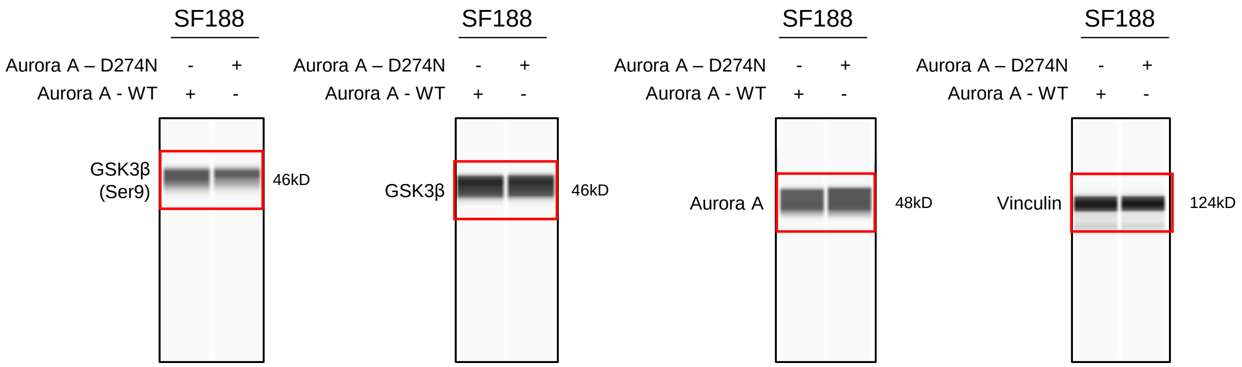

SF188
Aurora A – D274N - +
Aurora A - WT + -
GSK3β (Ser9)
46kD
SF188
Aurora A – D274N - +
Aurora A - WT + -
GSK3β
46kD
SF188
Aurora A – D274N - +
Aurora A - WT + -
Aurora A
48kD
SF188
Aurora A – D274N - +
Aurora A - WT + -
Vinculin
124kD

Figure S6d

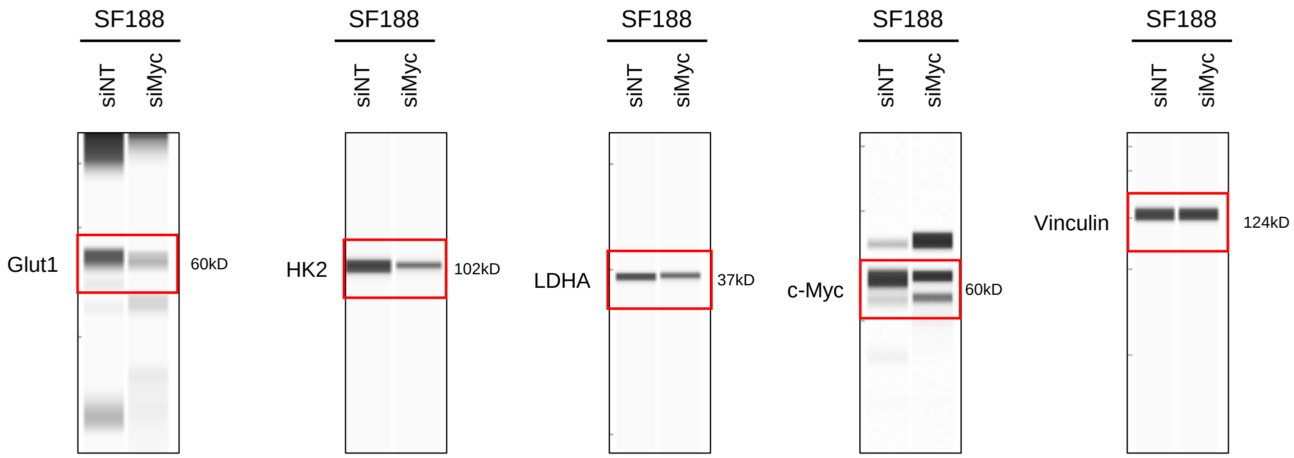
SF188
siNT
siMyc
Glut1
60kD
SF188
siNT
siMyc
HK2
102kD
SF188
siNT
siMyc
LDHA
37kD
SF188
siNT
siMyc
c-Myc
60kD
SF188
siNT
siMyc
Vinculin
124kD

Figure S6e

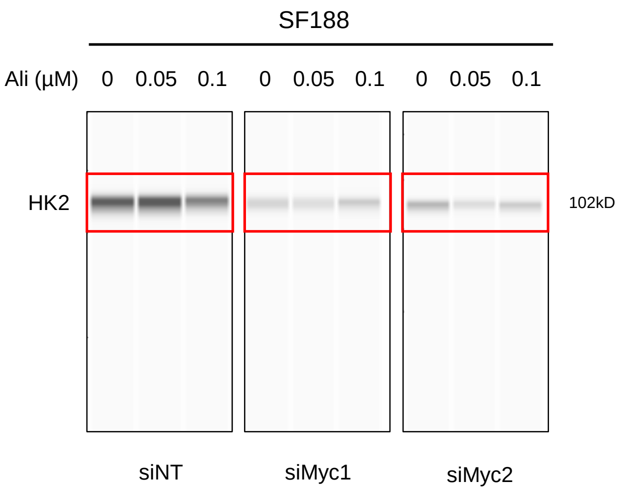
SF188
Ali (µM) 0 0.05 0.1 0 0.05 0.1 0 0.05 0.1
HK2
102kD
siNT
siMyc1
siMyc2

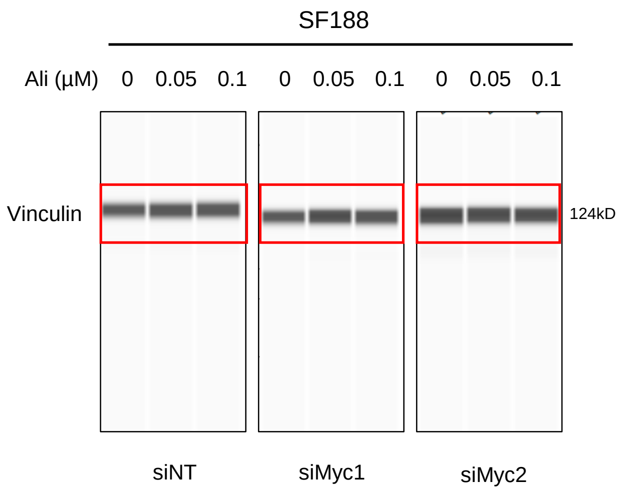
SF188
Ali (µM) 0 0.05 0.1 0 0.05 0.1 0 0.05 0.1
Vinculin
124kD
siNT
siMyc1
siMyc2

Figure S6g

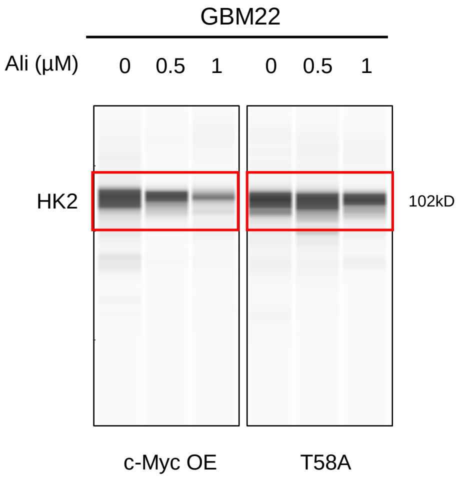
GBM22
Ali (µM)
0
0.5
1
0
0.5
1
HK2
102kD
c-Myc OE
T58A

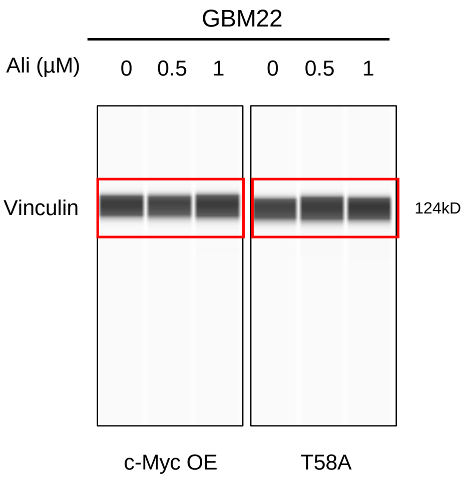
GBM22
Ali (µM)
0
0.5
1
0
0.5
1
Vinculin
124kD
c-Myc OE
T58A

Figure S9a

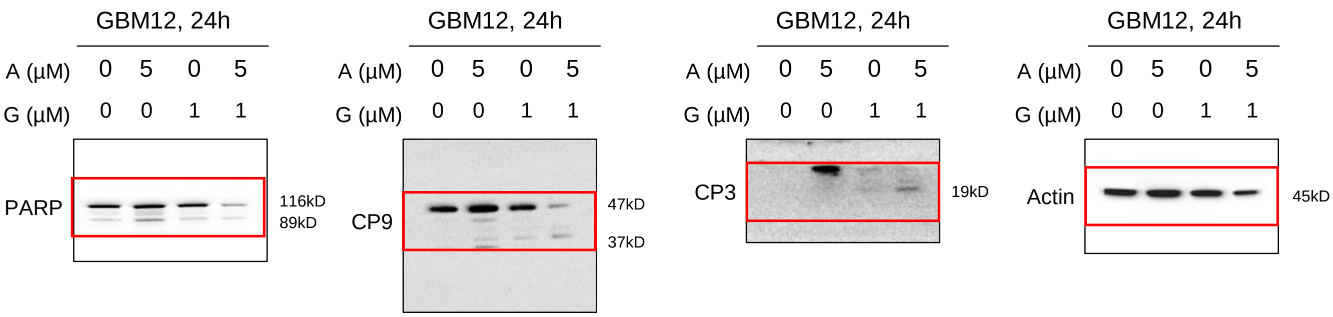
GBM12, 24h
A (μM) 0 5 0 5
G (μM) 0 0 1 1
PARP
116kD
89kD
GBM12, 24h
A (μM) 0 5 0 5
G (μM) 0 0 1 1
CP9
47kD
37kD
GBM12, 24h
A (μM) 0 5 0 5
G (μM) 0 0 1 1
CP3
19kD
GBM12, 24h
A (μM) 0 5 0 5
G (μM) 0 0 1 1
Actin
45kD

Figure S9b

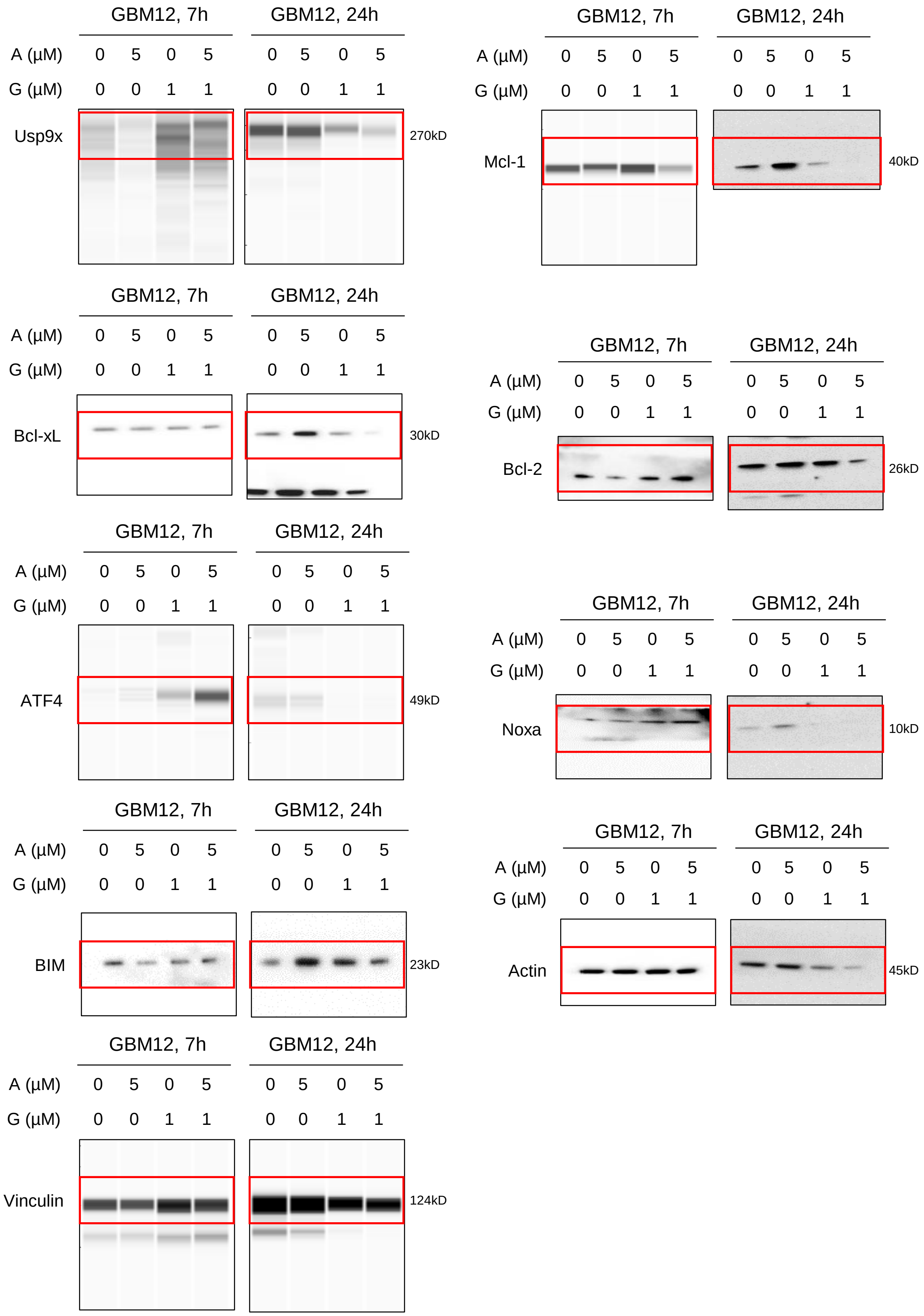
GBM12, 7h
GBM12, 24h
A (µM) 0 5 0 5 0 5 0 5
G (µM) 0 0 1 1 0 0 1 1
Usp9x
270kD
Mcl-1
40kD
Bcl-xL
30kD
Bcl-2
26kD
ATF4
49kD
Noxa
10kD
BIM
23kD
Actin
45kD
Vinculin
124kD

Figure S9b

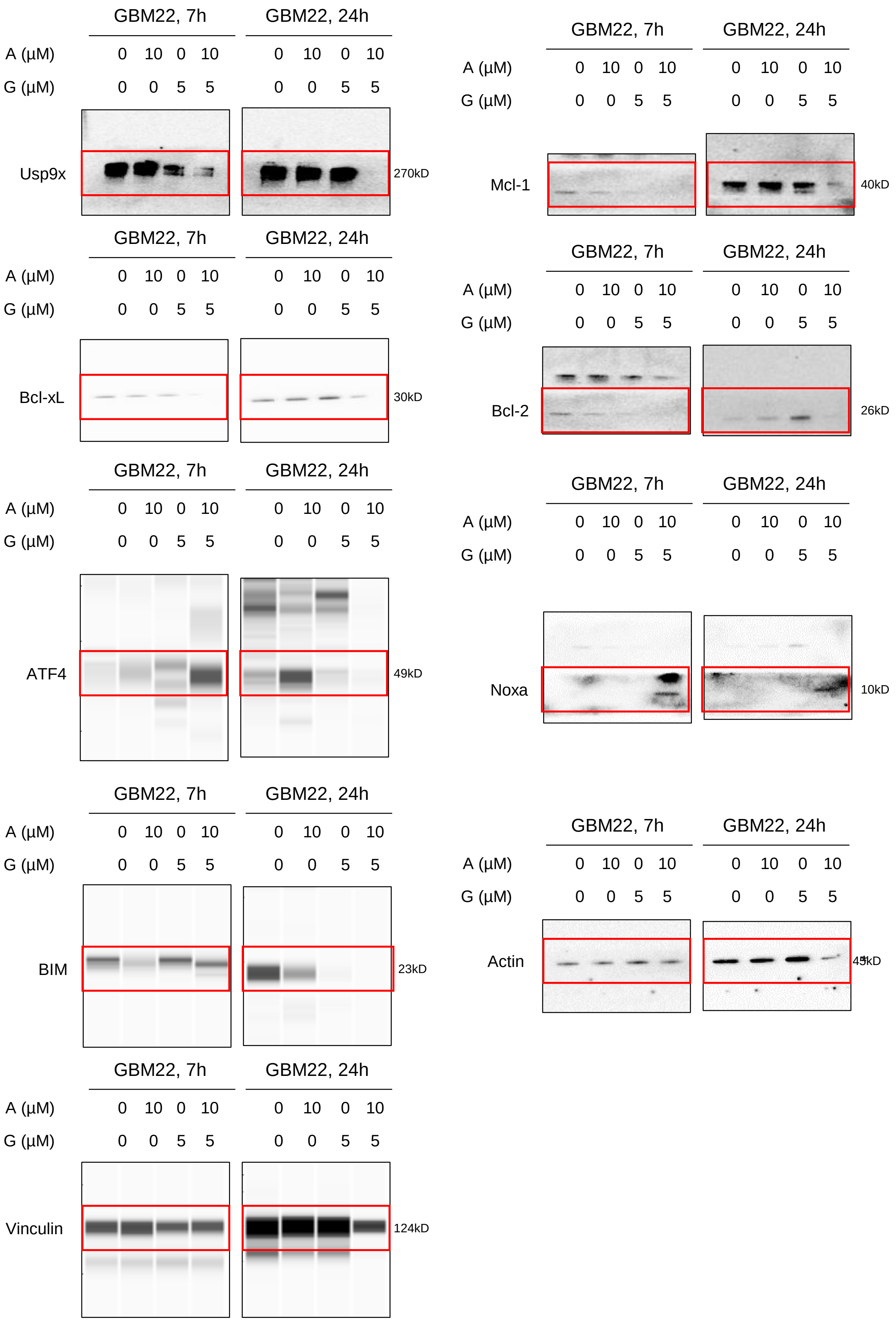
GBM22, 7h
GBM22, 24h
A (µM)
0 10 0 10
G (µM)
0 0 5 5
Usp9x
270kD
Mcl-1
40kD
Bcl-xL
30kD
Bcl-2
26kD
ATF4
49kD
Noxa
10kD
BIM
23kD
Vinculin
124kD
Actin
45kD

Figure S11c

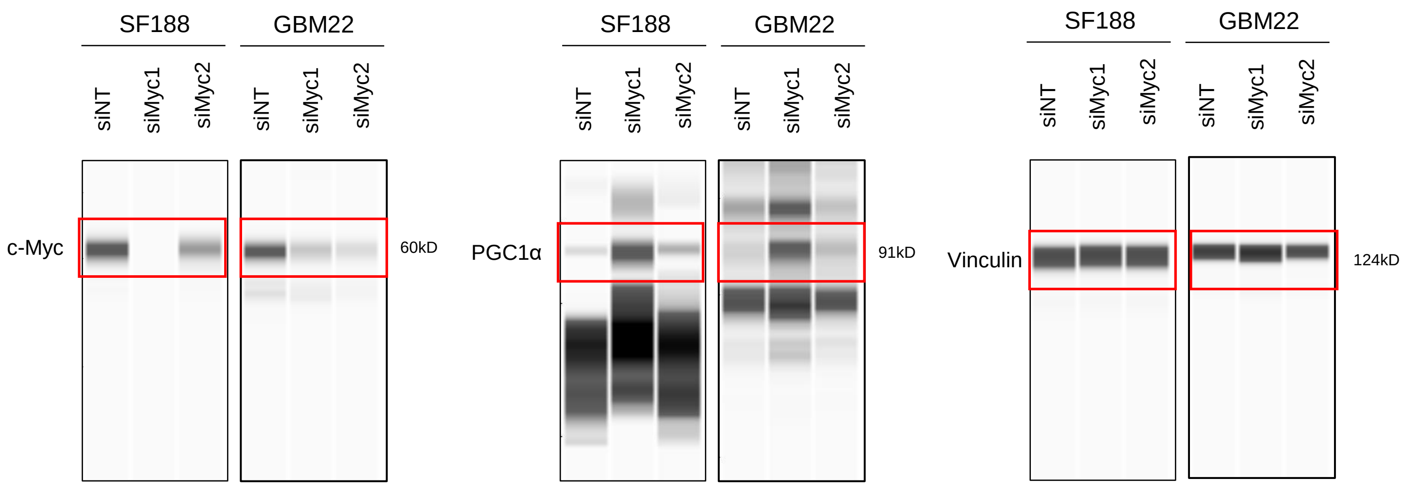

SF188
GBM22
siNT
siMyc1
siMyc2
siNT
siMyc1
siMyc2
c-Myc
60kD
SF188
GBM22
siNT
siMyc1
siMyc2
siNT
siMyc1
siMyc2
PGC1α
91kD
SF188
GBM22
siNT
siMyc1
siMyc2
siNT
siMyc1
siMyc2
Vinculin
124kD

Figure S11d

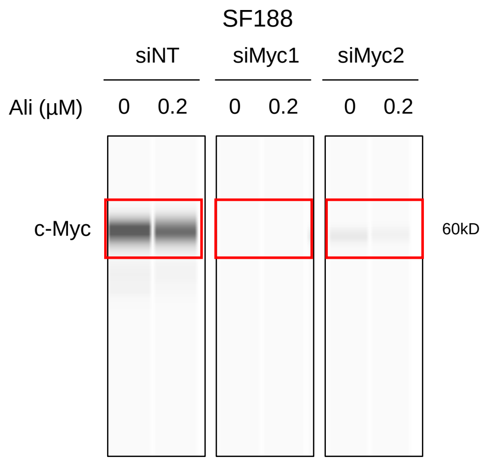
SF188
siNT
siMyc1
siMyc2
Ali (µM)
0
0.2
0
0.2
0
0.2
c-Myc
60kD

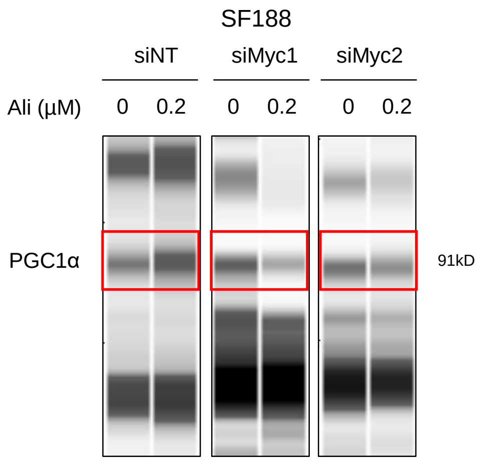
SF188
siNT
siMyc1
siMyc2
Ali (µM)
0
0.2
0
0.2
0
0.2
PGC1α
91kD

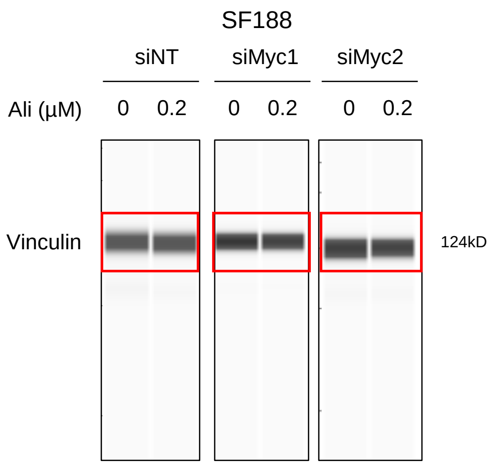
SF188
siNT
siMyc1
siMyc2
Ali (µM)
0
0.2
0
0.2
0
0.2
Vinculin
124kD

Figure S11e

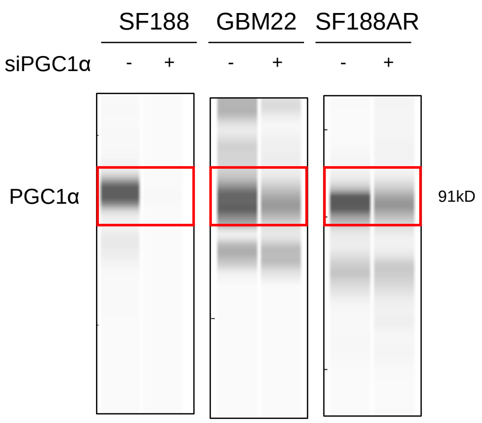
SF188
GBM22
SF188AR
siPGC1α
-
+
-
+
-
+
PGC1α
91kD
SF188
GBM22
SF188AR
siPGC1α
-
+
-
+
-
+
Vinculin
124kD
